# Best-of-Both-Worlds Predictive Approach to Dissociative Chemisorption on Metals

**DOI:** 10.1021/acs.jpclett.3c02972

**Published:** 2024-01-03

**Authors:** Andrew D. Powell, Nick Gerrits, Theophile Tchakoua, Mark F. Somers, Heriberto F. Busnengo, Jörg Meyer, Geert-Jan Kroes, Katharina Doblhoff-Dier

**Affiliations:** † Leiden Institute of Chemistry, Gorlaeus Laboratories, Leiden University, 2300 RA Leiden, The Netherlands; ‡ Instituto de Física Rosario (IFIR), CONICET-UNR, 2000 Rosario, Argentina; § Facultad de Ciencias Exatas, Ingeniería y Agrimensura, UNR, 2000 Rosario, Argentina

## Abstract

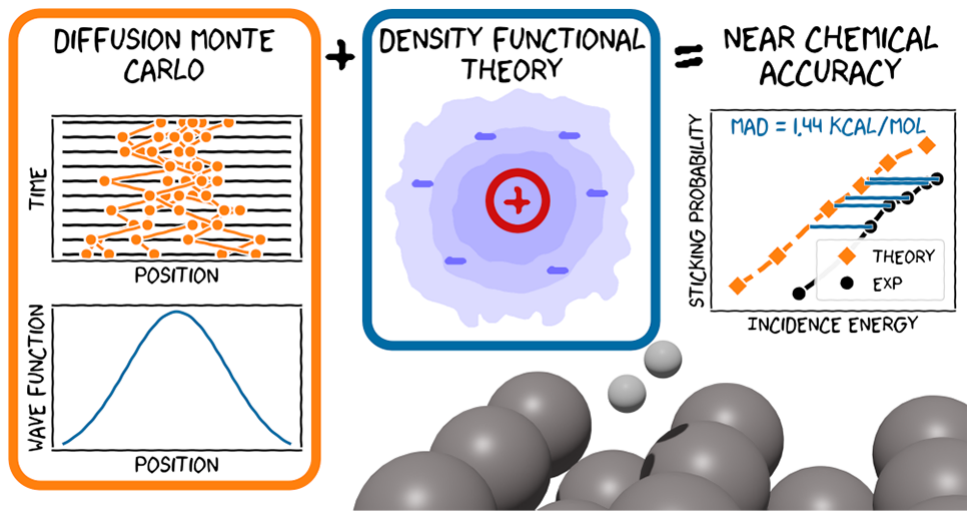

Predictive capability, accuracy, and affordability are essential features of a theory that is capable of describing dissociative chemisorption on a metal surface. This type of reaction is important for heterogeneous catalysis. Here we present an approach in which we use diffusion Monte Carlo (DMC) to pin the minimum barrier height and construct a density functional that reproduces this value. This predictive approach allows the construction of a potential energy surface at the cost of density functional theory while retaining near DMC accuracy. Scrutinizing effects of energy dissipation and quantum tunneling, dynamics calculations suggest the approach to be of near chemical accuracy, reproducing molecular beam sticking experiments for the showcase H_2_ + Al(110) system to ∼1.4 kcal/mol.

Barriers to dissociative chemisorption on transition metals control the rates of important heterogeneously catalyzed reactions.^1,2^ Because the production of the majority of chemicals involves heterogeneous catalysis at some stage,^3^ such barriers are obviously of practical importance. However, the accurate theoretical description of barrier heights (*E*_b_) for such systems presents a formidable intellectual challenge. Unlike for gas phase reactions,^4,5^ a first-principles method capable of computing barriers for reactions on metal surfaces with “chemical accuracy” (errors of ≤1 kcal/mol) is not yet available. For example, embedded correlated wave function theory, i.e., embedded CASPT2,^6,7^ yields only a semiquantitative description of O_2_ reacting on the surface of a simple metal [Al(111)].^7^ For H_2_ on Cu(111),^8^ embedded CASPT2 fails,^5^ possibly because the wave function theory used^8^ scales too unfavorably with the number of electrons to enable calculations on transition metals. Another first-principles method, diffusion Monte Carlo (DMC), gives near chemical accuracy for the H_2_ + Cu(111) system, reproducing the minimum *E*_b_ for this system to within 1.6 kcal/mol.^9^ Calculations on the BH76 database for gas phase reactions^10−12^ likewise demonstrate the high accuracy (errors of ∼1.2 kcal/mol) of DMC^13,14^ for reaction barriers. Because of its favorable scaling with system size,^13,14^ DMC can be applied to systems in which molecules react with transition metal surfaces.^9^ However, DMC is too computationally costly to compute an entire potential energy surface (PES). That is unfortunate because barrier heights are not observables. Their validation requires calculations with an appropriate dynamical method and model to enable comparisons with dissociative chemisorption probabilities measured in molecular beam experiments.^12^ This will usually require a PES and thus an affordable electronic structure method.

Density functional theory (DFT), the workhorse electronic structure method in computational heterogeneous catalysis, faces formidable challenges in treating barriers for dissociative chemisorption on metals. For gas phase reaction barriers, admixing exact exchange in the best functional (ωB97M-V) tested on the BH206 database reduced the mean absolute error to 1.7 kcal/mol.^4^ However, if one attempts to implement exact exchange for molecule–metal interactions, one runs into conflicting requirements on the range dependence. For fundamental reasons, gas phase systems require maximum exact exchange at long range, and indeed, the ωB97M-V density functional (DF) obeyed this condition.^15^ However, exact exchange must be screened at long distances within a metal.^16^ To the best of our knowledge, a hybrid DF meeting both requirements does not yet exist. Recent hybrid DFT calculations effectively using long-range screening^17^ achieved good agreement with semiempirical reference barriers,^5^ but these calculations erroneously used zero-point energy (zpe) corrections and surface atom relaxation in the presence of the molecule.^17^ For the worst case CH_4_ + Ni(111) system in their small database consisting of five systems, the errors made amounted to −2.8 kcal/mol due to the zpe correction^18^ and −2.3 kcal/mol due to allowing surface atom relaxation for the transition state calculation,^19^ yielding a total error of −5.1 kcal/mol. A problem for testing new DFs or for training new semiempirical DFs is that a representative database of dissociation barriers on metals is not yet available.^5^

At present, state-of-the-art chemically accurate dissociative chemisorption barriers are available for few (i.e., 14^5^) systems, which are characterized by limited charge transfer from the metal to the molecule.^20^ These barriers had to be obtained using a semiempirical DFT approach^12,21^ that requires well-documented experimental data.^5,12^ Tests employing a database with reference barriers for this limited class of systems show that the standard DFs used in surface science, i.e., DFs using semilocal exchange, yield errors in *E*_b_ of ≥2.4 kcal/mol.^5^

To go beyond the current state of the art, we need a fully predictive, as opposed to semiempirical, electronic structure approach that also works for systems with considerable charge transfer.^20^ In such systems, in which the molecule usually has a high affinity for electrons (making these systems potentially relevant to sustainable chemistry, e.g., oxygen-containing molecules), electronically non-adiabatic effects like electron–hole pair (ehp) excitation are likely to strongly affect the reaction dynamics.^22^ The accuracy of theories for dealing with these non-adiabatic effects in dynamics calculations on reactive scattering has not yet been established.^22−24^ Tuning a semiempirical DF in an attempt to compensate for errors introduced by an inaccurate non-adiabatic approach would likely result in serious errors in the reaction barrier.

A predictive approach is also needed for systems for which experiments are not available, are not well-documented, or yield conflicting results.^12^ Finally, a much more accurate approach than what is now available is needed if the field of computational surface reaction dynamics is ever to match the level of detail in the characterization of reaction mechanisms now available for gas phase reactions.^25^

How then should we proceed in view of the challenges described above? To obtain a predictive approach to dissociative chemisorption on metals that fulfills both requirements, of accuracy and affordability, we use the high observed accuracy of DMC for the minimum barrier height of H_2_ on Cu(111) and of gas phase reactions. We also use the finding that DFT is quite accurate for the variation of *E*_b_ with the system geometry. This is demonstrated by the success achieved with the previously mentioned semiempirical DFT method.^5,12^ Specifically, it was possible to reproduce measured sticking probability curves over large ranges of incidence energies (*E*_i_) by adjusting only one parameter in the semiempirical DF, with this parameter mainly affecting the minimum barrier height and therefore the threshold of the sticking curve. The fact that also the shape (width or, conversely, slope) of the curve was already well reproduced for 14 systems^5,12^ must^26^ mean that, in general, DFT is accurate for the variation of the barrier height with geometry, and of the PES in the vicinity of the transition state, once the minimum barrier height is pinned. This suggests an approach in which quantum Monte Carlo (QMC) (a family of accurate methods including DMC^13^) and DFT (with much lower computational costs) are fused. We call this approach the QMC-based DFT approach (QMC-DFT). In its simplest version, we construct a tunable DF [a quantum Monte Carlo-based density functional (QMC-DF)]. Instead of adjusting a parameter in this DF semiempirically as done earlier,^12,21^ we now adjust it so that the DF reproduces the DMC energy at a point near the transition state. The new method is therefore predictive and based on first principles. We also then rely on the ability of DFT to obtain the variation of the barrier height with the geometry right. According to the approximate but informative hole model,^26^ we should then be able to reproduce the sticking probability with a suitably chosen dynamical model and method.^12^ The hole model states that the dissociative chemisorption probability of a diatomic molecule equals the fraction of geometries (*X*, *Y*, cos θ, ϕ) (Figure 1A–C) for which the molecule’s energy exceeds *E*_b_(*X*, *Y*, cos θ, ϕ) in the associated two-dimensional potential (Figure 1D,E).

**Figure 1:**
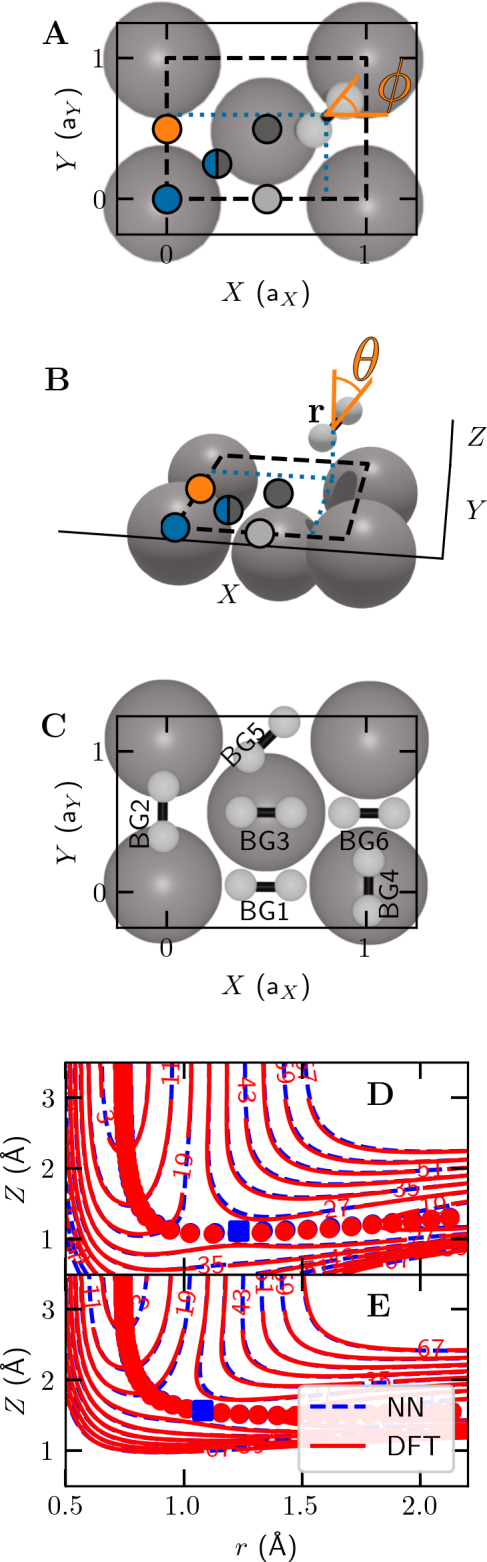
H_2_ + Al(110) system and PES for H_2_ being parallel to the surface. (A) Top view illustrating the (110) surface unit cell, the *X* and *Y* coordinates of the center of mass, and azimuthal orientation angle ϕ of H_2_. Colored solid circles represent the top (blue), long-bridge (light gray), short-bridge (orange), hollow (dark gray), and C sites (blue and gray). (B) Side view illustrating molecule–surface distance *Z*, H–H distance *r*, and polar orientation angle θ of H_2_. (C) Top view illustrating the six barrier geometries. (D) Elbow plot of the PES for the BG1 geometry (Table 1). Red (blue) circles indicate the DFT (neural network fitted) minimum energy path, and the red (blue) square indicates the position of the barrier. (E) Like panel D, but for BG2.

Here we demonstrate and present dynamics results for the simplest variant of our QMC-DFT approach. We apply our approach to the H_2_ + Al(110) system as a showcase, because DMC results^27^ and sticking probabilities are available for this system^28,29^ from well-documented molecular beam experiments.^28^

Table 1 compares the DMC energies computed for six barrier geometries [in reduced dimensionality, we call these barrier geometries BG1–BG6 (see Figure 1C)] to the energies computed with our QMC DF (for details, see Computational Methods and the Supporting Information). Overall, the barriers are reproduced rather well. Compared to DMC, the mean signed (absolute) error in the QMC-DFT *E*_b_ is 1.0 (1.6) kcal/mol. The QMC-DF is not so accurate for the *E*_b_ of BG2, which is close to that of BG1. Below we will show that this is not relevant for the case presented here, justifying the use of our straightforward variant of the QMC-DFT approach. A point worth noting from Table 1 is that comparison to DMC shows that the QMC-DF is rather good at describing the variation of the barrier height with geometry for the H_2_ + Al(110) system, the deviations from the DMC values being much smaller than the energy range spanned by the DMC energies of BG1–BG6. This observation was already made for the eight standard DFs compared to DMC for the H_2_ + Al(110) system previously^27^ and gives support to our earlier remark that the success of semiempirical DFT for DC on metals is due to DFT being good at describing the variation of the barrier height with system geometry.

**Table 1:** Geometries BG1–BG6 and Energies Associated with the DMC Barriers, Mean Signed Errors (MSE) and Mean Absolute Errors (MAE) in the QMC-DFT, and PBE Energies Computed for the DMC Barrier Geometries^a^

	site	ϕ (deg)	*r* (Å)	*Z* (Å)	*E*_b_^DMC^	*E*_b_^QMC-DF^	*E*_b_^PBE^
BG1	long bridge	0	1.334	1.118	25.1 ± 0.2	25.4 (0.3)	19.5 (−5.6)
BG2	short bridge	90	1.080	1.568	26.7 ± 0.2	25.1 (−1.6)	19.7 (−7.0)
BG3	hollow	0	1.245	0.615	35.1 ± 0.2	37.6 (2.5)	26.6 (−8.5)
BG4	top	90	1.368	1.564	36.6 ± 0.2	37.8 (1.2)	29.5 (−7.1)
BG5	long bridge	45	1.361	0.786	33.7 ± 0.2	34.9 (1.1)	26.2 (−7.5)
BG6	short bridge	0	1.154	1.175	47.0 ± 0.2	49.6 (2.6)	38.1 (−8.9)
MSE						1.0	–7.4
MAE						1.6	7.4

a DMC barrier geometries, DMC barrier heights *E*_b_^DMC^, QMC-DFT energies *E*_b_^QMC-DF^, and PBE energies *E*_b_^PBE^ are provided. H_2_ is always parallel to the surface (θ = 90°). Differences between the QMC-DFT (PBE) and the DMC energies are provided in parentheses in the column showing *E*_b_^QMC-DF^ (*E*_b_^PBE^). All energies are in kilocalories per mole.

Relative to experiment, the sticking probability (*S*_0_) curve computed with the Born–Oppenheimer static surface (BOSS) approximation, but for a thermally expanded surface as appropriate for the experimental surface temperature (*T*_s_), appears to be shifted to a lower *E*_i_ by 1.51 kcal/mol (Figure 2A and Figure S13). This difference reflects not only errors in the QMC-DFT approach but also errors due to the simplifications of the dynamical model, which we now seek to minimize.

**Figure 2:**
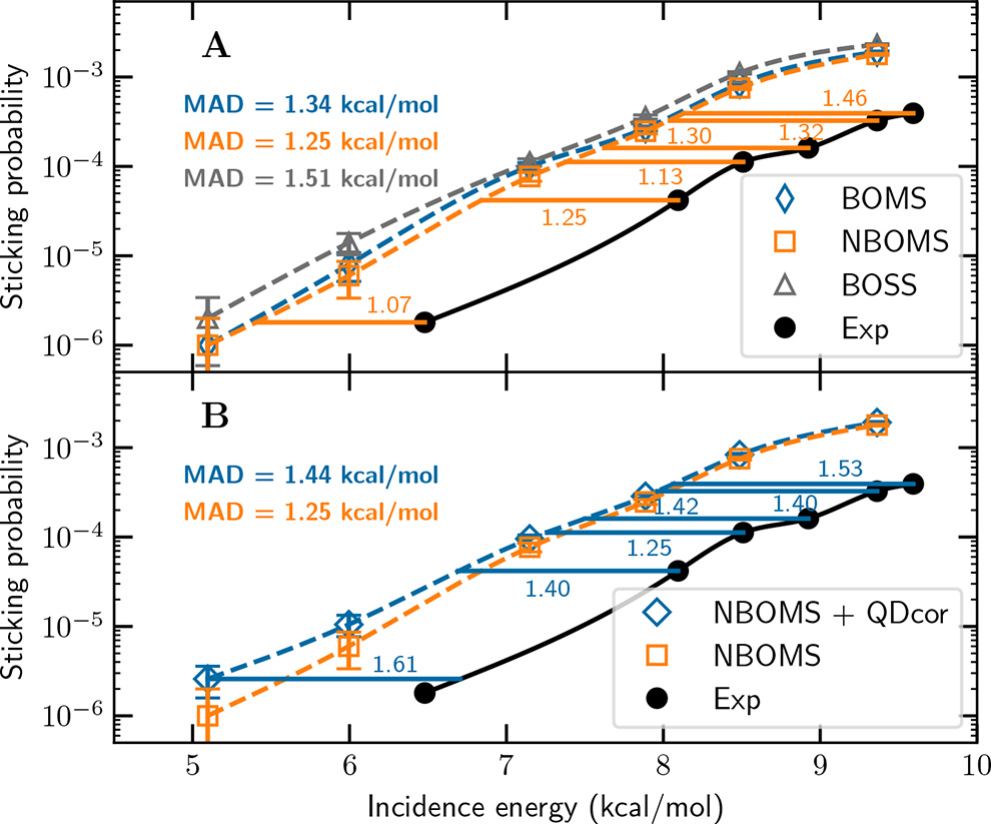
Computed and measured *S*_0_ values for the H_2_ + Al(110) system.^28,29^ (A) *S*_0_ values computed with the BOSS, BOMS, and NBOMS models compared to experimental values. The orange horizontal lines and numbers (kilocalories per mole) indicate the energy distance between the measured *S*_0_ and the *S*_0_ computed with the NBOMS model. (B) *S*_0_ values computed with the NBOMS model, and as computed with the NBOMS model but also corrected for quantum dynamical effects, are compared to experimental values. The blue horizontal lines and numbers (kilocalories per mole) indicate the energy distance between the measured *S*_0_ and the *S*_0_ computed with the NBOMS model corrected for nuclear quantum effects.

Introducing surface atom motion [doing quasi-classical trajectory (QCT) calculations with the Born–Oppenheimer moving surface (BOMS) model^12^] improves the dynamical model. The resulting reduction of the *S*_0_ leads to better agreement with experiment by effectively moving the whole computed *S*_0_ curve to a higher *E*_i_ by ≈0.2 kcal/mol (Figure 2A and Figure S14). The reduction is consistent with an activated dissociative chemisorption mechanism at a low *T*_s_ dominated by mechanical rather than electronic coupling^30,31^ and energy dissipation to surface atoms through surface atom recoil.^31^ As a result, the shift between the computed and measured *S*_0_ curves is reduced to 1.34 kcal/mol (Figure 2A and Figure S15).

Also introducing ehp excitation improves the dynamical model further. [The resulting model has been called the non-Born–Oppenheimer moving surface (NBOMS) model.^12^] Using the local density friction approximation (LDFA)^32^ to model ehp excitation in molecular-dynamics-with-electronic-friction calculations further reduces the computed *S*_0_ (Figure 2A). A similar effect of ehp excitation on sticking has been found in calculations on H_2_ reacting on Cu and Ag surfaces, where the reduction in *S*_0_ was attributed to the dissipation of energy to the electrons of the metal.^32−34^ The reduction moves the computed *S*_0_ curve to an even higher *E*_i_ by an additional 0.10 kcal/mol (Figure 2A and Figure S16). Adding ehp excitation to the model thus leads to further agreement with experiment, the computed *S*_0_ curve being shifted relative to the experiment by 1.25 kcal/mol (Figure 2A).

Finally, a correction for nuclear quantum effects on the motion of H_2_ is added. For computational reasons, nuclear quantum effects cannot be included for a moving surface or when ehp excitation is included while modeling motion in all molecular degrees of freedom exactly. Instead, we simply assume the nuclear quantum corrections to be independent of the effects of surface atom motion and ehp excitation. Then, its effect on the NBOMS sticking probability can be estimated from the difference between quantum dynamical and QCT sticking probabilities computed with the BOSS model.^35^ Using a straightforward correction procedure (adding the mentioned difference between the quantum dynamical and QCT sticking probabilities, procedure A in the Supporting Information) moves the sticking probability to a lower *E*_i_ by ≈0.18 kcal/mol. Consequently, the corrected *S*_0_ curve is shifted relative to the experiment by 1.44 kcal/mol (Figure 2B). The comparison between the QMC-DFT and experimental *S*_0_ therefore suggests that the minimum barrier height computed with DMC for the H_2_ + Al(110) system is accurate to within ∼1.5 kcal/mol.

A few points are worth emphasizing with regard to panels A and B of Figure 2. The first point is relevant to the accuracy with which the QMC-DF reproduces the DMC energies of BG3–BG6. The errors in BG3 and BG6 may appear rather large (2.5 and 2.6 kcal/mol, respectively). Figure 2B suggests that the computed quantum corrected sticking probability is not sensitive to such discrepancies over the range of *E*_i_ for which experimental results are available for validation: the computed sticking curve appears to be shifted relative to the interpolated experimental curve by a reasonably constant energy shift, ranging between 1.25 and 1.63 kcal/mol. In this particular case, this may well be because the DMC energies of BG3 and BG6 are higher than that of BG1 by ≥10 kcal/mol. The second point is that according to Table 1, a dynamics calculation like the quantum corrected one now presented in Figure 2B but based on the PBE DF would have been of essentially no predictive value for the H_2_ + Al(110) system. The PBE DF underestimates the DMC energy of BG1 (by ∼6 kcal/mol), and the QMC-DF sticking curve is shifted to lower energies relative to the experimental one by approximately −1.5 kcal/mol. One would then expect the sticking curve computed on the basis of a PBE PES to be shifted relative to experiment by approximately −7.5 kcal/mol. This is yet another illustration that standard GGA DFs cannot be expected to allow accurate predictions for sticking curves for DC on metal surfaces.^12^ In contrast, our results suggest that parametrizing a DF on the basis of the DMC transition state energy, as done here, allows predictions for DC on metal surfaces of near chemical accuracy. Furthermore, calculations on the H_2_ + Cu(111) system using PESs calculated with different DFs and different minimum barrier heights^21^ suggest that analogous calculations on the H_2_ + Al(110) system with the PBE and the QMC DFs should yield qualitatively different results for rotationally and diffractive inelastic scattering^36^ and very different predictions for experiments on vibrationally inelastic scattering.^37^ The third and final point is that the effects of allowing surface atom motion and ehp excitation, on one hand, and tunneling motion, on the other, are small and tend to cancel each other. As one can see from panels A and B of Figure 2, the MADs computed with the BOSS model and the BOMS model corrected for tunneling are 1.51 and 1.44 kcal/mol, respectively, yielding a very similar conclusion regarding the quality of QMC-DFT for the H_2_ + Al(110) system.

Our calculations yield interesting details of the reaction dynamics, as investigated with the BOMS model. Reacting molecules originally aimed at specific high-symmetry sites (Figure 3A) tend to react at these sites (Figure 3B). This justifies the analysis of the reactivity in terms of site-specific reaction probabilities (Figure 3C). Interestingly, the site-specific reaction probability is highest at the long-bridge site (BG1) even though BG2 has the lowest *E*_b_ with the QMC-DF (Table 1). This surprising finding can be explained as follows. At low *E*_i_ values, the reaction probability is dominated by molecules that are initially in the *v* = 1 vibrationally excited state (see Figure 3D and Figure S18). Polanyi’s rules^38,39^ dictate that vibrationally excited diatomic molecules react more efficiently at geometries with “later barriers”, where the H_2_ bond is more elongated. BG1 at the long-bridge site shows a barrier that is considerably lower than that of BG2 at the short-bridge site (Table 1 and Figure 1D,E), explaining the larger reaction probability at the long-bridge site.

**Figure 3:**
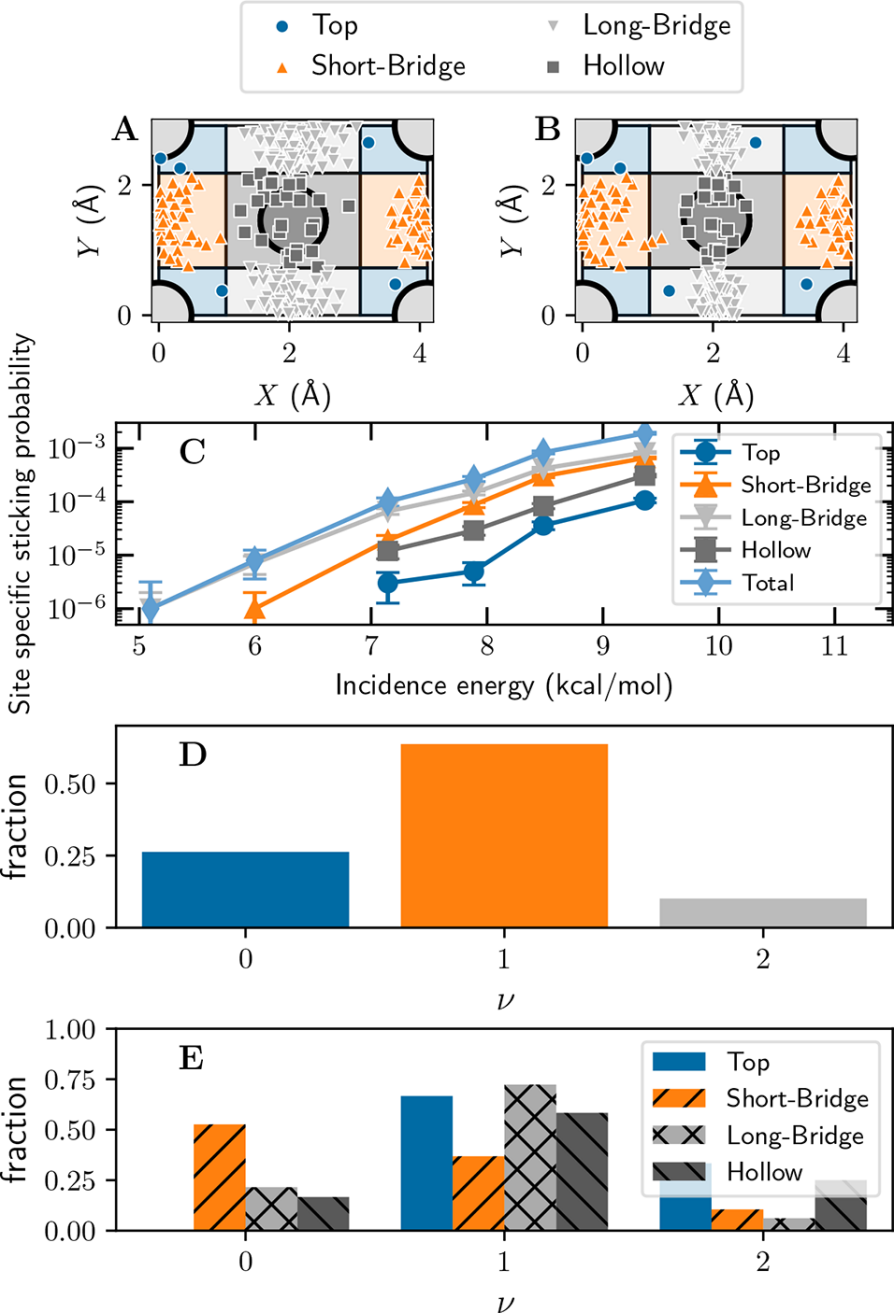
Detailed dynamics results. (A) Impact sites [initial (*X*, *Y*)] of the reactive trajectories computed for an *E*_i_ of 7.2 kcal/mol. Light gray triangles indicate impacts on the long-bridge site, orange triangles impacts on the short-bridge site, dark gray squares impacts on the hollow site, and blue circles impacts on the top site. (B) Like panel A but showing (*X*, *Y*) at the time of reaction. (C) Total and site-specific reaction probabilities computed with the BOMS model as a function of *E*_i_. (D) Fractions initially incident in the *v* = 0, 1, and 2 vibrational states of the molecules that react at an *E*_i_ of 7.2 kcal/mol. (E) Fractions of the molecules initially incident in the *v* = 0, 1, and 2 vibrational states of the molecules that react at a specific impact site at an *E*_i_ of 7.2 kcal/mol.

Building further on this analysis allows us to estimate the effect that the underestimation of the value of *E*_b_ at the BG2 short-bridge geometry has on our results. To this end, we subtract site-specific sticking probability *S*_0_^SB^ at the short-bridge site [BG2 (see Figure 3C)] from total reaction probability *S*_0_ and add the *S*_0_^SB^ curve evaluated for a 1.6 kcal/mol lower *E*_i_ to make up for the overly low fitted QMC-DF barrier at BG2 (see Table 1). Performing this procedure using the BOMS model moves the computed *S*_0_ curve up, somewhat improving agreement with experiment, but by only ∼0.11 kcal/mol (Figure S17). This small change strongly suggests that the inaccurate description of BG2 has a minor effect on the results presented here. Therefore, it justifies our focus on our current simple implementation of the QMC-DFT approach, in which we simply fit a QMC-DF to the energy of the BG1 transition state.

Our comparison between theory and experiment, which uses dynamics calculations based on our implementation of the QMC-DFT approach, suggests an accuracy of 1.5 kcal/mol in the minimum barrier height obtained with DMC for the H_2_ + Al(110) system. This finding is consistent with the accuracy obtained earlier with DMC for the *E*_b_ of the benchmark dissociative chemisorption system [H_2_ + Cu(111) (1.6 kcal/mol^9^)] and with the accuracy of DMC established for gas phase *E*_b_ (1.2 kcal/mol for the BH76 database^10−12^). The available evidence suggests that when using DMC, dynamics calculations based on QMC-DFT are capable of reproducing measured probabilities for dissociative chemisorption on metal surfaces with near chemical accuracy (errors of ∼1.5 kcal/mol).

The accuracy of the QMC-DFT approach can be improved in a systematic way. In the QMC component, the main challenge is to reduce the error due to the fixed-node approximation of DMC.^13,14,40^ Fixed-node errors can be reduced by starting a DMC calculation from a multideterminant wave function,^41,42^ which can in principle be generated with extensions of DFT.^43,44^ Starting a DMC calculation from a multideterminant wave function has already been shown to provide an improved *E*_b_ for gas phase reactions.^45^ With appropriate developments, it may be possible to replace DMC in the future with a potentially more accurate QMC method that avoids the fixed-node approximation by addressing the underlying sign problem in a different way.^46^ The combination of DMC with machine learning methods may in the future also allow applications to larger systems.^47^ In the DFT component, a challenge may be to develop DFs that can fit the minimum *E*_b_ for systems affected by charge transfer, but this can likely be done using screened hybrid functionals.^20^ Ascending the rungs on Jacob’s ladder of DFs might also further improve the DF’s ability to reproduce the variation of *E*_b_ with system geometry beyond that already achieved using DFs with semilocal exchange here and elsewhere.^12,27^ If the DF remains inaccurate for this variation, one can partition the PES into the molecule–surface interaction and the potential describing the solid.^48^ Then, in the calculation of the molecule–surface interaction, one can make the parameter in the QMC-DF dependent on *X*, *Y*, and ϕ using symmetry-adapted functions.^49,50^ This has been done earlier for potential expansion functions.^50^ In the future, it will probably be possible to derive a true DMC-quality PES by adding a high-dimensional neural network (HDNN) PES based on the difference between, say, 1000 DMC energies and a QMC-DFT PES as obtained here, in the spirit of the Δ-machine learning approach recently used to obtain a CCSD(T) level PES for MD simulations of liquid water.^51^ We expect that with such systematic improvements the QMC-DFT approach can ultimately attain chemical accuracy (errors of ≤1 kcal/mol) in the description of experiments of dissociative chemisorption on metals.

Our success with QMC-DFT suggests the following approach to achieve further substantial progress with modeling of dissociative chemisorption on metals. It will obviously be important to obtain DMC values of the minimum *E*_b_, as used here, especially for systems prone to charge transfer that are hard or impossible to treat with a semiempirical approach. At first, it will remain important to use these data to design a QMC-DF and perform dynamics calculations comparing to experimental data for further validation of the approach, as done here. However, from the start, the DMC data obtained with this validation and other DMC *E*_b_ data can be collected in databases to eventually develop a representative database with the minimum *E*_b_ for dissociative chemisorption on metals, taking a data science approach to extend existing databases.^5^ Such a database can then be used to assess the performance of new electronic structure methods, including those of new DFs that would attempt to solve the conundrum associated with the range dependence of exact exchange for molecule–metal surface systems. For this, one could make the fraction of exact exchange depend simultaneously on the environment of both electrons involved, using the kinetic energy density analogously as in made-simple meta-GGA DFs.^52^ Strategies for developing such DFs could include the use of machine learning DFs, as has already been used for gas phase systems.^53^

Here we have applied a novel, predictive, and computationally efficient electronic structure approach for dissociative chemisorption on surfaces, called QMC-DFT, to the H_2_ + Al(110) system taken as a showcase. We chose this system because sticking probabilities are available for it from well-documented molecular beam experiments, which allowed validation. We have derived a QMC DF by fitting an appropriate DF expression to the energy of the lowest DMC barrier found for the system. We have next used the QMC-DF to construct a HDNN PES. By simulating reaction probabilities and comparing with experiment using a hierarchy of dynamics models and correcting for quantum effects, we have demonstrated that the tested QMC-DFT approach exhibits near chemical accuracy (error of ≈1.4 kcal/mol) for sticking of H_2_ on Al(110). The QMC-DFT approach can be systematically improved and can be applied to and is likely as accurate for DC of molecules on transition metal surfaces. The success of the approach suggests a road map using data science and machine learning to achieve further substantial progress in modeling dissociative chemisorption on metals.

## Computational Methods

To demonstrate the simplest variant of our approach, we proceed as follows. First, we construct a QMC-DF for the H_2_ + Al(110) system by optimizing a parameter in this DF to make the DFT and the QMC minimum barrier heights match. Employing the QMC-DF in direct dynamics calculations would be computationally too costly due to the low reaction probabilities of H_2_ on Al(110). We therefore use the QMC-DF to perform DFT calculations for ∼36 000 geometries and fit a HDNN PES to these data,^54,55^ which were computed for the surface temperature (*T*_s_) used in the experiments we model (220 K^28,29^). This PES allows us to perform statistically relevant molecular dynamics calculations at an increasing level of sophistication. By additionally applying quantum corrections,^35^ we can then reliably demonstrate the accuracy of the QMC-DFT approach for the H_2_ + Al(110) system.

Our choice of the QMC-DF
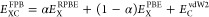
1is a weighted average of RPBE^56^ and PBE^57^ semilocal exchange with the vdW-DF2 nonlocal correlation functional added to approximately describe the dispersion energy.^58^ Tuning the parameter α in eq 1 effectively varies the exchange-enhancement factor of the functional for large gradients of density.^56^ We choose α = 0.71 to reproduce the DMC energy at the barrier geometry with the lowest DMC energy (BG1). Further details are provided in the Supporting Information.

QMC-DFT energies have been computed for the H_2_ + Al(110) system at the experimental surface temperature (*T*_s_ = 220 K) as described in the Supporting Information. A HDNN fit to these data yields an accurate description of both the DMC energies for BG1–BG6 as adjusted for *T*_s_ and the QMC-DFT energies (see Table S7 and the elbow plots in panels D and E of Figure 1 showing two-dimensional cuts through the HDNN PES compared to the raw DFT data).

Using the computed HDNN PES, sticking probabilities have been computed with the QCT method using a hierarchy of models, i.e., the BOSS, the BOMS, and the NBOMS models that have been described in a recent review.^12^ Quantum corrections have been applied to QCT results acquired with the NBOMS model on the basis of quantum dynamics results obtained earlier with the BOSS model for the H_2_ + Al(110) system.^35^ We have investigated the effect of allowing surface atom motion because the mass ratio of H_2_ to Al is more favorable to energy transfer than that of H_2_ to Cu^59^ in the much investigated H_2_ + Cu(111) benchmark system (see, e.g., ref (60) and also Section S1 of the Supporting Information). We have investigated the additional effect of ehp excitation with the NBOMS model because the value of the charge transfer energy (i.e., the work function of the surface minus the electron affinity of the molecule) is lower for the H_2_ + Al(110) system (7.4 eV^20,61^) than for the H_2_ + Cu(111) system (8.1 eV^20^), for which the effect of ehp excitation has been investigated earlier (see, e.g., ref (33)). The reason is that lower charge transfer energies have been found to correlate with greater electronically non-adiabatic effects.^22^ Finally, we have investigated the importance of tunneling because the measured maximum sticking probability for the H_2_ + Al(110) system (≈0.4 × 10^–4^)^29^ is lower than the maximum value measured for the H_2_ + Cu(111) system (≈0.1^62^) by >2 orders of magnitude. Relative effects of the transfer of energy to surface atom motion and related to ehp excitation are also likely to become more important for small *S*_0_ values, which is another reason to investigate the effect of these dissipation channels on a comparison of theory versus experiment for the H_2_ + Al(110) system. Details of the methods used are presented in the Supporting Information.

## Supporting Information Available

General computational setup for the H_2_ + Al(110) system (Section S1), QMC calculations and results (Section S2), quantum Monte Carlo-based density functional (Section S3), setup for the QMC-DFT calculations (Section S4), high-dimensional neural network potential (Section S5), quasi-classical trajectory calculations (Section S6), molecular dynamics with electronic friction calculations (Section S7), quantum corrections to the NBOMS sticking probabilities (Section S8), importance of the use of the correct velocity distributions (Section S9), fractions of fitted QMC-DFT data exhibiting specific fitting errors for different DFT energy ranges (Figure S1), comparison of DFT data and HDNN fitted data for potential elbows for BG1–BG6 (Figure S2), accuracy of the HDNN fit in the van der Waals region (Figure S3), accuracy of fitted electronic and mechanical coupling to surface atom motion (Figure S4), electronic friction coefficient of the hydrogen atom as a function of *r*_s_ (Figure S5), electronic friction coefficients for H_2_ + Al(110) and Cu(111), and N_2_ + Ru(0001) (Figure S6), energy conservation in MDEF calculations with Ermak and Buckholz integration (Figure S7), energy conservation in MDEF calculations with Grønbech-Jensen and Farago integration (Figure S8), comparison of quantum dynamical and QCT initial state-specific reaction probabilities (Figure S9), comparison of QCT sticking probabilities computed with PESs fitted with the corrugation reducing procedure and the HDNN method (Figure S10), comparison of QCT sticking probabilities computed with the NBOMS model and with the same model but with corrections for quantum effects with one another and with experimental results (Figure S11), comparison of QCT sticking probabilities computed assuming different velocity distributions with one another and with experimental results (Figure S12), comparison of QCT sticking probabilities computed with the BOSS model and measured in experiments (Figure S13), comparison of QCT sticking probabilities computed with the BOSS model and with the BOMS model with one another (Figure S14), comparison of QCT sticking probabilities computed with the BOMS model with measured sticking probabilities (Figure S15), comparison of QCT sticking probabilities computed with the BOMS model and with the NBOMS model with one another (Figure S16), comparison of QCT sticking probabilities computed with the NBOMS model with and without corrections for the too low QMC DFT barrier of BG2 with measured sticking probabilities (Figure S17), computed fractions of reacting molecules initially incident in different vibrational states (Figure S18), computed fractions of reacting molecules initially incident on a specific surface site and in different vibrational states (Figure S19), QMC-DFT energies at DMC barrier geometries for *T*_s_ values of 0 and 220 K (Table S1), hyperparameters of the HDNN PES (Tables S2–S5), information about the training of the HDNN PES (Table S6), single-point energies for DMC barrier geometries computed with DMC, QMC-DFT, the HDNN PES, and the CRP PES for a *T*_s_ of 220 K (Table S7), and saddle point energies computed with QMC-DFT, the HDNN PES, and the CRP PES for a *T*_s_ of 220 K (Table S8) (PDF)
Transparent Peer Review report available (PDF)
