# Supplementary material for: Best-of-Both-Worlds Predictive Approach to Dissociative Chemisorption on Metals
Source: J Phys Chem Lett. 2024 Jan 3;15(1):307–15. doi: 10.1021/acs.jpclett.3c02972 (PMC10788952; doi:10.1021/acs.jpclett.3c02972)
Supplement: Supplementary file 1 — jz3c02972_si_001.pdf [file jz3c02972_si_001.pdf]

Supporting information to: Best-of-both-worlds Predictive Approach to Dissociative Chemisorption on Metals

*Andrew D. Powell[1] †, Nick Gerrits[1] †, Theophile Tchakoua[1] †‡, Mark F. Somers[1], Heriberto F. Busnengo[2,3], Jörg Meyer[1], Geert-Jan Kroes[1] *, Katharina Doblhoff-Dier[1] **

[1]Leiden Institute of Chemistry, Gorlaeus Laboratories, Leiden University, 2300 RA Leiden, The Netherlands.

[2]Instituto de Física Rosario (IFIR), CONICET-UNR, 2000 Rosario, Argentina.

[3]Facultad de Ciencias Exatas, Ingeniería y Agrimensura, UNR, 2000 Rosario, Argentina.

† These authors have contributed equally to this work.

‡: Present address: Debye Institute for Nanomaterials Science, Universiteitsweg 99, Utrecht University, 3584 CG Utrecht, The Netherlands.

S1. General computational set-up for H_2 + Al(110)

The (110) unit cell of Al(110) is depicted in Fig.1A. It is rectangular in shape, with a long side (along *X*) and a short side (along *Y*). The molecular coordinates of H_2 relative to Al(110) are shown in Figs.1A and 1B. The center-of-mass motion of H_2 is represented by the coordinates *X* and *Y* for motion along the surface, and by the distance *Z* of the molecule to the surface (i.e., the top layer of the Al(110) surface). Furthermore *r* is the distance between the H-atoms and the orientation of the molecule is specified by the polar and azimuthal angles θ and φ, where the former is measured relative to the surface normal and the latter with respect to the *X*-axis.

Our quantum Monte-Carlo based density functional theory (QMC-DFT) calculations are based on the results of diffusion Monte-Carlo (DMC) calculations performed for six minimum barrier geometries (BG1-BG6 in Fig.1C) computed with the PBE functional [1] of DFT. These DMC calculations have been described in detail in Ref. [2]. To put the calculations presented in the main paper and below in context, a brief summary is supplied in Section S2 below.

In QMC-DFT as we use it here a parameter in a density functional (DF) is adjusted to achieve, as much as possible, agreement of the DFT calculations with a DMC calculations for a single crucial geometry. QMC-DFT bears a superficial resemblance with specific reaction parameter DFT (SRP-DFT) for molecules interacting with metal surfaces [3-5]. In SRP-DFT a parameter is adjusted in a DF to achieve agreement between measured sticking probabilities (here: interchangeable with dissociative chemisorption probabilities) and sticking probabilities computed in a dynamics calculation on the basis of the SRP density functional. However, QMC-DFT is fully *predictive*, whereas SRP-DFT is a *semi-empirical* theory through the way the DF was always constructed in previous applications to molecules reacting on metal surfaces [5]. In Section S3 we describe how the adjustable parameter in the QMC density functional (QMC-DF) was adjusted to reproduce the DMC energies for BG1 closely.

In Section S4 we describe some details of the QMC-DFT calculations. This includes a description of the basic model of the system as it will also be described in the dynamics

calculations. Here, the Al(110) surface is modeled by a slab of 10 Al layers, employing a (3x3) surface unit cell to avoid interactions between H_2 and its periodic images in the plane wave DFT calculations becoming important.

In Section S5 we describe how the QMC-DFT data were used to construct a high-dimensional neural network potential (HDNNP) in which the H_2 molecule interacts with an Al(110) surface at the surface temperature (T_s) of 220 K used in the experiments we compare to[6]. Here, the Al atoms in the upper 4 layers, with which H_2 interacts most directly, are allowed to move. With the way in which the Al(110) surface is modeled (also using a (3x3) surface unit cell with 9 atoms per layer), the potential energy surface (PES) constructed in this way depends on 6 (for H_2) + 36x3 (for the surface) = 114 degrees of freedom (DOFs).

This work describes dynamics calculations using three models. In the simplest model, the Born-Oppenheimer static surface (BOSS) model [5], the Born-Oppenheimer approximation is made and the static surface approximation is made, with the surface atoms held in their ideal lattice positions (here for a 220 K surface). In the Born-Oppenheimer moving surface (BOMS) model (Ref.[5] and references therein), the BO approximation is retained, but the surface atoms are allowed to move in order to be able to model the effect of surface atom motion at a specified T_s (here the experimental value, T_s = 220 K [6]) on the sticking probability (S_0). Keeping the surface atoms fixed is usually a very good approximation for molecules sticking to cold transition metal surfaces [5]. However, according to the Baule model, the mass ratio of H_2 (mass 2) and Al (mass 27) is more conducive to energy transfer than the mass ratio of, for instance, H_2 and Cu (mass 64). For this reason, and because the measured sticking probabilies are as small as 2 x 10^{-6}, we also perform BOMS calculations on H_2 + Al(110). The quasi-classical trajectory (QCT) method used for the BOMS calculations (and, effectively, the BOSS calculations) is described in Section S6. BOSS and BOMS calculations are performed for the same initial conditions regarding the motion of H_2 in order to isolate, as much as possible, the effect of allowing surface motion on the sticking.

In the third model, the non-Born-Oppenheimer moving surface (NBOMS) model (Ref.[5] and references therein) we additionally model the effect of electron-hole pair (ehp) excitation, using the local density friction approximation (LDFA) [7]. The molecular dynamics with electronic friction (MDEF) method used is described in Section S7. BOMS and NBOMS calculations are performed for the same initial conditions regarding the motion of H_2 and of the surface atoms in order to isolate, as much as possible, the effect of allowing ehp excitation on the sticking.

Finally, previous quantum dynamics (QD) and QCT calculation on H_2 + Al(110) within the BOSS model [8], which used a differently fitted PES, have been used here to make an estimate of the size of quantum corrections to the sticking probabilities computed with the NBOMS model. For this reason a very brief description of this differently fitted PES and of the QD and QCT calculations performed with it is provided in Section S8.

S2. QMC calculations and results

The QMC-DF described below has been fitted to fixed-node quantum Monte-Carlo (QMC) calculations described in detail elsewhere [2], and we will only give a summary here.

To start the QMC calculations, calculations were performed with the general purpose PBE [1] functional of DFT [9-10]. First, two transition state geometries (BG1 and BG2, see Fig.1C) and four barrier geometries in reduced dimensionality (BG3-BG6, see Fig.1C) were obtained with PBE-DFT calculations. The Al(110) surface was described using the PBE lattice constant and PBE interlayer distances, for reasons detailed in Ref.[2]. The calculations used the VASP 5.3.5 code [11-12] to compute DFT energies for single points and the dimer method [13-16] to determine barrier geometries of the molecule relative to the surface, as depicted in Fig.1C and presented in Table 1. Details of these calculations have been provided in Ref.[2].

Next the QMC energies were computed in three different stages [2]. First, a PBE DFT calculation was performed for each molecular BG relative to the Al(110) slab, as previously computed with PBE-DFT with the PBE Al(110) slab. However, with a view to the QMC

calculations to follow the new PBE-DFT calculations were done for Al(110) slab geometries corresponding to experimental measurements of the Al lattice constant and the interlayer distances between layers 1 and 2 (d_{12}) and between layers 2 and 3 (d_{13}) [17], as detailed in Ref.[2]. These calculations were performed with the Quantum Espresso package [18]. This provided a Kohn-Sham determinant for each BG. The resulting Kohn-Sham wave function was multiplied with a Jastrow function containing up to three-body terms, the coefficients of which were optimized in a variational Monte-Carlo (VMC) calculation [19-20] in which the objective function to be minimized was the energy expectation value. The resulting wave function was used as trial wave function in a diffusion Monte-Carlo (DMC) calculation [21-23], in which the optimization was performed [2] by propagating the wave function according to the imaginary time Schrödinger equation [24]. Pseudo-potentials were treated using the T-move scheme [25]. Single particle finite size errors were mitigated by using a twist averaging procedure described in detail in Ref.[2]. Many-particle finite size errors were mitigated by extrapolating to infinite system size using supercell sizes of (2x2) surface atoms with one H_2 molecule attached on top and bottom and another slab with (4x4) surface atoms with four H_2 molecules attached on top and bottom [2]. The VMC and the DMC calculations were performed using the CASINO package [26-27]; for full details of the calculations, see Ref.[2]. The DMC energies obtained for the six BGs are provided in Table 1. BG1 corresponds to the lowest energy DMC transition state (TS), with an energy of 25.1 kcal/mol. The energy of BG2 (26.7 kcal/mol) is higher by only 1.6 kcal/mol. The energies of these BGs are lowest because these geometries allow the H-atoms to move to their preferred chemisorption sites (the top sites). The energies of BG3-BG6 are much higher, varying between 35.1 and 47.0 kcal/mol. As discussed in an exhaustive error analysis presented in Ref. [2] and the Supporting Information to Ref.[2], the statistical error in the DMC barrier heights is estimated to be 0.2 kcal/mol, while the systematic error in the DMC barrier heights is expected to fall well below 1 kcal/mol.

S3. The quantum Monte-Carlo based density functional (QMC-DF)

The QMC-DF [3, 5] was chosen according to

$$E_{XC}^{FPB} = \alpha E_X^{RPBE} + (1-\alpha) E_X^{PBE} + E_C^{vdW2} \qquad \text{S1.}$$

Here, E_X^{RPBE} is the exchange part of the RPBE DF [28], E_X^{PBE} is the exchange part of the PBE DF [1], and E_C^{vdW2} is the non local vdW-DF2 Chalmers-Rutgers correlation functional allowing an approximate description of the attractive Van der Waals dispersion interaction [29]. One reason that we chose to use the vdW-DF2 correlation functional is that, compared to DMC, the use of the RPBE-vdW-DF2 functional leads to an overestimate of the TS energy, whereas the use of the RPBE [28] or the RPBE-vdW-DF1 [28, 30] functional does not [2]. Also, the use of the vdW-DF2 correlation functional yields an approximate description of the Van der Waals interaction (see also below), whereas the use of the GGA PBE correlation functional (which is also incorporated in the RPBE functional [28]) does not.

To obtain a good description of the barrier height corresponding to BG1, in Eq.S1 the choice $\alpha = 0.71$ was made. Table 1 compares the QMC-DFT energies computed at the six DMC barrier geometries with the DMC values. With the choice made, the DMC energy of the TS at the BG1 geometry is overestimated by 0.3 kcal/mol, but the QMC-DF underestimates the DMC energy at the BG2 geometry by 1.6 kcal/mol. The mean signed error (MSE) in the computed barrier height is 1.0 kcal/mol when averaging over all six barrier geometries, and the DMC energies for the geometries BG3-BG6 are overestimated by 1.2 - 2.6 kcal/mol (see Table 1). Originally we expected that the net effect of the "fitting error" made when replacing a hypothetical DMC potential energy surface (PES) with the present QMC-DFT PES would be that using the QMC-DFT PES would lead to an overestimate of the sticking probability. We made that assumption because we expected the computed sticking probability to depend most strongly on the lowest two barriers, of which one is underestimated by 1.6 kcal/mol. However, as

we will show below and discuss briefly in the main manuscript, the fitting error in the BG2 barrier height has little effect on the computed sticking probability for dynamical reasons.

The mean absolute error (MAE) in the QMC-DFT barrier height is 1.6 kcal/mol. We note that the actual barriers are much larger (between 25.1 and 47.0 kcal/mol with DMC), making the relative errors in the barrier heights rather small. The low value obtained for the MAE underlines our previous finding that DFT is quite good at reproducing the variation of barrier heights with geometry [2], as might also have been deduced from the previous success of SRP-DFT. Tuning the absolute value of the barrier height to obtain good agreement with QMC calculations should then result in low MAEs characterizing the agreement between QMC-DFT and the first principles theory, as observed here.

S4. Set up for the QMC-DFT calculations

The QMC-DFT calculations on which the potential energy surface discussed below was based have been performed with the VASP 5.3.5 code [11-12].

We first set up a 10-layer slab modeling the Al(110) surface at 0 K. Here, the dimensions of the surface unit cell in the ***x*** and ***y*** directions were taken according to the crystal lattice constant computed with the QMC-DF. The interlayer distances in the Z direction normal to the surface were relaxed and thereby computed with the QMC-DF as well. A full account of how the 0 K Al(110) surface was modeled and set up is provided in Section S1 of the Supporting Information (SI) to Ref.[8].

The input parameters to the QMC-DFT calculations of the molecule-surface interaction energies were based on convergence tests on DFT energies computed for the QMC BG1 and BG2 geometries of the molecule relative to the surface. A full account of these tests and of the input parameters used in the QMC-DFT calculations is given in Section S2 of the SI to Ref.[8]. Based on these convergence tests, we expect the QMC-DFT calculations of the molecule-surface

interaction energy to be converged to within better than 0.5 kcal/mol. The QMC-DFT barrier heights shown in Table 1 are for the 0 K set-up of the Al(110) surface.

The experiments were done for a surface temperature (T_s) of 220 K[6]. It is therefore appropriate to adjust the dimensions of the slab accordingly. The adjustment was done on the basis of the QMC-DFT values of the Al crystal lattice constant and the distances between the top layers of the Al(110) slab, and on experimental information of how these parameters change with temperature. A full description of how this was done is given in Section S3 of the SI to Ref.[8]. In the QMC-DFT calculation of the interaction energy between the molecule and the 220 K slab model of the Al(110) surface the same DFT input parameters were used as derived for the 0 K model of the slab.

Using the 220 K slab leads to slightly different QMC-DFT barrier heights for the DMC barrier geometries as obtained using the 0 K QMC-DFT slab. The 220 K QMC-DFT values are compared with the 0 K QMC-DFT values in Table S1. As can be seen, the 220 K barrier heights are lower by up to 0.3 kcal/mol. The energy differences tabulated in Table S1 can be used to estimate how the DMC values would change upon the thermal distortion of the Al(110) surface going from 0 K (as used in the DMC calculations) to the experimental surface temperature of 220 K.

S5. The high-dimensional neural network potential (HDNNP)

Here we discuss the fitting of the QMC-DFT data for the H_2 + Al(110) system, for the case that the surface atoms are allowed to move. While we used 10 Al layers to compute the molecule-surface interaction (as discussed in Section 4 above), only the atoms in the four upper layers were allowed to move (see e.g. Section 4 above). As we used a (3 x 3) surface unit cell in the DFT calculations [8] as well as in the fit, this means that the effect of the motion in 4 x 9 = 36 Al atoms is modeled in the HDNNP. Taking into account that the motion of 2 H atoms is

modeled in addition, below we describe the development of a 114D PES, i.e., a PES with 114 degrees of freedom.

To represent the H_2-Al(110) interaction through a potential energy surface, QMC-DFT data have been fitted to a HDNNP using the method of Behler and Parinello [31]. This method may be viewed as a second-generation machine-learning method for fitting potentials (MLP) [32-33]. In these methods, as detailed below, the total energy is written as a sum of atomic contributions (atomic neural networks, ANNs), which describe the local environment. This is done with the use of atom-centered symmetry functions (ACSFs) [31, 34], through which translational and permutation symmetry are enforced. To achieve this, only one type of ANN is used for each chemical element [32-33]. The ACSFs may be viewed as "structural fingerprints of the local environments [32]. Furthermore, each ANN is represented by a feed-forward neural network [33]. In previous applications of the method to fit HDNNP's the method was shown to be accurate for the description of the barriers in the PES and the overall description of the PES, including the electronic and mechanical couplings that are important to the effect of surface atom motion on reaction [35-36]. The method has also been shown to yield an accurate description of a metal surface's vibrational properties (i.e., its phonon band structure and density of states) [35]. Below, Section S5.1 describes the analytical form of the neural network, Section S5.2 describes how the HDNNP was trained, and Section S5.3 describes how the HDNNP compares with the QMC-DFT data it was fitted to.

S5.1 Analytical form of the HDNNP

In the HDNNP description of the H_2 + Al(110) PES, the energy of the system was written as a sum of atomic contributions according to

$$V = \sum_{i}^{N_{atom}} V_i \qquad \text{S2.}$$

Here, N_{atom} is the number of atoms in the system (i.e., 92, there are 90 Al atoms, and 2 H atoms). V_i is the energy contribution from atom i in its specific environment from an ANN. For each atom type (i.e., atom of a specific chemical element), there is a separate ANN describing the contribution to the total potential of the atom of that type. In the method of Behler and Parinello [31], the ANN is defined in terms of predefined descriptors. In each ANN, the local environment of the atom is defined by a cut-off function as follows:

$$f_c(R_{ij}) = 0.5\left[\cos\left(\frac{\pi R_{ij}}{R_c}\right)+1\right], \text{ if } R_{ij} \leq R_c \qquad \text{S3a}$$

$$f_c(R_{ij}) = 0, \text{ if } R_{ij} > R_c \qquad \text{S3b.}$$

Above, R_{ij} is the distance of the atom for which the ANN is defined (atom *i*) to the atom *j* in its environment. For the cut-off radius R_c a value of 13.5 bohr was selected in this work.

As already alluded to above, use is made of atom-centered symmetry functions to enforce the appropriate symmetries. To achieve this, the coordinates of the atoms are transformed to radial and angular ACSFs. The radial ACSFs used in this work are written as

$$G_{rad,i} = \sum_{j\in R_c} e^{-\eta\left(R_{ij}-R_s\right)^2} \times f_c(R_{ij}) \qquad \text{S4.}$$

Here, η and R_s are called hyperparameters (parameters that control the machine learning process, but that are not derived by training), which define the shape of a Gaussian sphere around the central atom [32]. The hyperparameters defining the radial ACSFs used in this work are presented in Tables S2 and S3. A total of 33 symmetry functions is used to describe the pair interactions, i.e., 6 for the H-H interactions, 10 for the H-Al interactions, 10 for the Al-H interactions, and 7 for the Al-Al interactions. Here, the atom mentioned first is the reference atom for which the

ANN is defined, and the second atom is in its immediate environment, i.e., within the cutoff radius of it. Symmetry functions for identical pairs are taken the same. The value of R_s is taken as 0 bohr.

The angular ACSFs used in this work are written as

$$G_{ang,j} = 2^{1-\zeta} \sum_{j,k \neq i} \left(1+\lambda \cos\theta_{ijk}\right)^{\zeta} e^{-\eta\left(R_{ij}^2+R_{ik}^2+R_{jk}^2\right)} f_c(R_{ij}) f_c(R_{ik}) f_c(R_{jk}) \qquad \text{S5.}$$

Here, θ_{ijk} is the angle between the lines connecting atoms i and j and atoms i and k, and ζ, λ, and η are the hyperparameters. The hyperparameters ζ and λ defining the angular ACSFs used in this work are presented in Table S4 for the H-atom ANN and in Table S5 for the Al-atom ANN. The η hyperparameters are taken as zero in the angular symmetry functions (meaning that we could have omitted the exponential term on the right hand side (rhs) of Eq.S5). We have used 11 symmetry functions for each of the triples H-H-Al, H-Al-Al, Al-H-H, and Al-H-Al, and 12 symmetry functions for Al-Al-Al. Identical parameters were used for the H-H-Al and the Al-H-H triples. Identical parameters were also used for the H-Al-Al and Al-H-Al triples.

Finally, each ANN is represented by a feed-forward neural network [33] consisting of 2 hidden layers with 20 nodes each. We used hyperbolic tangent activation functions, and no bias weights.

S5.2 Training the network

In the construction of the neural network, the RuNNer package [34, 37-38] was used. The atomic configurations used to train the neural network for H_2 + Al(110) have been sampled from the dynamically relevant region of the PES in different stages (see also Table S6). In stage 1, emphasis was put on determining the dependence of the PES on the molecular coordinates, and the surface was therefore held fixed at the structure derived from DFT from experiment. More

specifically, we have used the 220 K Al(110) slab as determined from DFT relaxation calculations with appropriate scaling according to experimental information on lattice expansion and on how interlayer distances change with temperature (see also Section S4 above). About 1000 points were picked with "gas phase geometries", i.e., sampling the X, Y, θ, and ϕ geometries at random, while selecting R and Z at random within the ranges indicated in Table S6. About 4000 points were picked with "solid geometries", i.e., sampling the X, Y, θ, and ϕ geometries at random, while selecting R and Z at random closer to the surface, again within the ranges indicated in Table S6.

At stage 2, approximately 15,000 points with distorted surface geometries were added, with approximately 3000 points at gas phase geometries and 12,000 points at solid geometries. The molecular geometries were sampled as in stage 1. However, now also random displacements were carried out of the surface atoms in the upper 4 layers. The surface Al atoms were thermally distorted from their equilibrium positions according to random Gaussian distributions. For each atom the standard deviation is taken as

$$\delta_\mu = \sqrt{\frac{kT_s}{m_{Al}}} \Big/ \omega_\mu \quad , \ \mu = \mathrm{x, y, z} \qquad \text{(S6)}$$

where m_{Al} is the mass of a surface atom, and ω_μ is the frequency of motion in one of the three Cartesian displacement directions of the atom, as labeled by μ. In doing so, displacements were sampled not only for the experimental value of T_s (220 K), but also for higher values, up to 900 K. The frequencies ω_μ used were taken from QMC-DFT calculations on the Al(110) slab and were in the range 18.9-26.1 meV.

At stage 3, classical trajectories were run with the LAMMPS code [39-40]. The RuNNer package [34, 37-38] was used in prediction mode, to signal if the HDNNP might be of insufficient accuracy to describe the system for a specific configuration that is encountered in a trajectory. If

such an "extrapolation error" is made, an additional DFT calculation is performed for the relevant geometry, which is then added to the training set. This is done in sub-stages. In total, about 16,000 points were added to the training set at this stage, sampling both additional molecular geometries and distortions of the surface.

A criterion used throughout for whether or not the point should be included in the reference set was that the potential energy of the full system (relative to H_2 in the gas phase in its rovibrational ground state and the surface in its equilibrium configuration) should not exceed 10 eV. In total, 35,853 structures (i.e., geometries + corresponding energies and forces) were added to the reference data set. Of these data 90% were used for training, and 10% for testing.

S5.3 Comparison of the HDNNP to DFT data

To judge the quality of the neural network fit of the QMC-DFT data, we first computed root-mean-squared errors (RSMEs) for the training set (32,327 data points) and the test set (3526 data points), comparing to directly calculated QMC-DFT data. The values obtained for the RSMEs for the training set (28.8 meV) and the test set (33.2 meV) show that the HDNNP obtained describes the QMC-DFT data with better than chemical accuracy (1 kcal/mol ≈ 43 meV) in the region of coordinate space that is relevant to the sticking of H_2 on Al(110). Furthermore, Fig.S1A shows that, as one might then expect, a large fraction (about 90 %) of the data have fitting errors smaller than 1 kcal/mol associated with them. This is even more true for DFT reference energies < 50 kcal/mol, of which the overwhelming part of the data are described within chemical accuracy by the HDNNP (see Fig.S1B).

Another measure of the accuracy of the HDNNP fit is how accurately the fit reproduces the QMC-DFT barrier heights computed for the DMC barrier geometries BG1-BG6. We find that the QMC-DFT barrier heights are reproduced quite well, with a MSE of -0.4 kcal/mol, and a MAE of 0.7 kcal/mol (see Table S7). Obviously, it is also of interest how well the HDNNP reproduces the DMC barrier heights on which the fit of the QMC-DF was based. To determine

this, we first corrected the DMC barrier heights for thermal effects in an approximate way, assuming that their temperature dependence should be equal to the temperature dependence of the QMC-DFT values

$$E_b^{DMC}(220\ \mathrm{K}) = E_b^{DMC}(0\ \mathrm{K}) + E_b^{QMC-DFT}(220\ \mathrm{K}) - E_b^{QMC-DFT}(0\ \mathrm{K}) \qquad \text{(S7).}$$

In Eq.S7, $E_b^{DMC}(220\ \mathrm{K})$ (see Table A4) is the DMC barrier height for a hypothetical metal surface at 220 K in which the Al atoms occupy their equilibrium positions, and $E_b^{DMC}(0\ \mathrm{K})$ is the barrier height as computed with DMC in Ref.[2] (see Table 1). The QMC-DFT barrier heights in Eq.S7 may be found in Table S1. The HDNNP also reproduces the DMC barrier heights estimated for 220 K well, with a MSE of 0.6 kcal/mol and a MAE of 1.3 kcal/mol. The HDNNP reproduces the DMC energy of the TS (the BG1 energy), but underestimates the similarly low energy of BG2 by 1.9 kcal/mol. Even though the MSE is positive (by 0.6 kcal/mol), one might suspect that the too low energy of geometry BG2 may lead to too low values of the computed sticking probabilities. Here, the main problem comes from the fitting of the QMC-DF to the DMC data and not from fitting a HDNNP to the QMC-DFT data. As Table S7 (and also Table 1) shows, the QMC-DF that we derived reproduces the DMC energy of the DMC-TS (i.e., of BG1) quite well, but leads to an underestimate of the energy of BG2. However, we note again that we will show in the manuscript and below that the underestimation of the BG2 barrier height hardly leads to an overestimation of the sticking probabilities, for dynamical reasons. Finally, through chance the HDNNP is actually a better fit to the DMC barrier heights estimated for 220 K than the "fits" of the QMC-DFT 0K barrier heights to the DMC 0K barrier heights: for the latter case the discrepancies may be characterized by a MSE of 1.0 and a MAE of 1.6 kcal/mol (see Table 1).

Another view of the quality of the HDNNP fit to the QMC-DFT data may be obtained from elbow plots of the data. Elbow plots of the HDNNP and of the raw QMC-DFT data are shown in Fig.S2. As can be seen the HDNNP is a faithful representation of the QMC-DFT data in the

dynamically relevant regions of Z and r for the six geometries indicated. The HDNNP also gives a good description of the QMC-DFT data in the region of the van der Waals well depth, as can be seen for the TS1 geometry in Fig.A4. The van der Waals minimum of our HDNNP, 39.5 meV is in good agreement with the value (40.4 meV) determined by experiments on selective adsorption of H_2 on Al(110) [41]. This result is consistent with the observation that the vdW-DF2 DF (in which the correlation DF we use is however combined with a different exchange DF [29]) yields a good description of the van der Waals well for H_2 + Cu(111) [42].

Another important measure of the quality of the HDNNP fit is how well it describes the couplings between the translational motion of the molecule and the motion of the surface atoms. Couplings that are relevant to how surface atom motion affects the dissociative chemisorption of a molecule on a surface [5, 43-44] are the so-called mechanical coupling α and the electronic coupling β. The definitions of these couplings take into account that, to a good approximation, the distance of the barrier to the surface is given by

$$Z_b(Q) = Z_b(0) + \alpha Q \qquad \text{(S8)}$$

while the barrier height is given by

$$E_b(Q) = E_b(0) - \beta Q \qquad \text{(S9)}$$

where Q is a surface atom displacement coordinate. Figure S4 shows these linear relationships reflected in both the QMC-DFT data and the HDNNP. This is shown for the variation of the barrier height (Fig.S4A) and the barrier location (Fig.S4B) with the displacement of the nearest neighbor top layer surface atom to which one of the H-atoms dissociate in the BG1 barrier geometry. Here, $Q > 0$ corresponds to the surface atom coming out of the surface. The α and β parameters determined from the HDNNP fit ($\alpha = 0.43$ and $\beta = 7.0$ kcal mol^{-1} Å^{-1}) are in good

agreement with the parameters determined from direct linear fits to the QMC-DFT data (α = 0.42 and β = 6.6 kcal mol^{-1} Å^{-1}).

Finally, a good criterion for the accuracy of the fit is whether it ultimately yields the same results for the quantity of interest as another accurate fit, when used in dynamics calculations. Below, in Section S8.2, we will show that this is the case.

S6. Quasi-classical trajectory calculations

S6.1 Equilibration of the Al(110) surface at the experimental surface temperature

The experimental surface temperature ($T_s = 220$ K) is taken into account by expanding the lattice constant computed with DFT by 0.67% on the basis of experimental measurements [17] regarding bulk expansion of Al. The interlayer distances between the upper layers of the Al(110) slab were taken according to DFT calculations and to how interlayer distances change according to experiments [17]. A full description of how this was done has been given in Section 3 of the Supporting Information of Ref.[8].

Next the Al(110) surface was equilibrated at 220 K by explicitly simulating surface atom motion as described in detail in Section 4 of the Supporting Information of Ref.[36]. Briefly, in the BOMS and NBOMS calculations the initial positions and velocities of the surface atoms are taken from the Boltzmann distribution for ($T_s = 220$ K), using frequencies in the range of 0.4-0.6 kcal/mol (18.9 - 26.1 meV). Fifty surfaces were randomly initialized in this way. Starting from these initial configurations these surfaces have been equilibrated for 1 ps, using a 1 fs time step in NVE simulations (constant number of particles N, volume V, and total energy E) and the LAMMPS code [39-40], making checks that the total energy remained at the value corresponding to the temperature to be imposed. Subsequently, the fifty NVE simulations have been continued an additional ps. Each time step a snapshot has been stored, yielding a total of 50,000 snapshots. For each trajectory, a random snapshot is taken to specify the initial conditions of the surface atoms.

In the BOSS calculations, the Al atoms occupied their ideal lattice positions in an Al(110) surface with its dimensions adapted to the experimental surface temperature of 220 K. In the BOSS calculations motion occurs in the six molecular degrees of freedom, while in the BOMS and NBOMS calculations the motion is modeled in 6 + 3 x 36 = 114 degrees of freedom.

S6.2 Sampling of initial conditions

In the simulation of supersonic molecular beam experiments on sticking of H_2 on metal surfaces it is highly important to take the properties of the molecular H_2 beams into account: Sticking probability curves (i.e., the sticking probability vs. the average incidence energy) measured at different labs may be shifted along the incidence energy axis relative to another by more than 1 kcal/mol due to differences in the beams employed at these labs [3, 5, 45]. It is therefore vital to give a correct description of the pure H_2 molecular beams that were used to obtain the sticking probabilities presented in figure 9 of Ref. [6] for H_2 + Al(110), to which we compare.

Fortunately, such a description is available. A molecular beam can be defined through the nozzle temperature T_n (which governs the rovibrational state distribution of H_2 in the beam) and the flux weighted velocity distribution of the H_2 molecules. The latter distribution can be characterized by the stream velocity v_0 and the velocity width parameter α [3, 46-47]. The flux weighted velocity distribution is given by

$$f(v;T_n)dv = Cv^3 \exp\left[-\left(v-v_0\right)^2/\alpha^2\right]dv \qquad \text{(S10)}$$

A crucial point is that the v_0 and α parameters of Eq.S10 are available from information presented in the PhD thesis [48] of one of the authors of Ref.[6]. Here it is of crucial importance that the thesis also presented the experimental H_2 + Al(110) results we compare to: Figure 5.39 of the thesis [48] is the above mentioned figure 9 of Ref.[49]. In particular, this thesis presented time-of-

flight (TOF) distributions for T_n = 1100, 1400, and 1700 K (Fig.4.4 of the thesis) and a plot of the speed ratio of the beam vs. average incidence energy (Fig.4.8 of the thesis). From these data, v_0 and α parameters could be derived as described in the Supporting Information to Ref. [3] for the values of T_n stated above as well as for other values, including T_n = 1120, 1330, and 1580 K. The relevant values of these parameters can be found in Table 1 of Ref.[8]. They are identical to the values presented in Tables S5 and S6 of Ref.[3].

Another crucial point is that the validity of the parameters extracted from the thesis of Berger has been established [3] independently in calculations on H_2 and D_2 on Cu(111), for which the thesis of Berger [48] also presented results. Specifically, in Ref. [3] a semi-empirical specific reaction parameter density functional (SRP-DF) was fitted to sticking coefficients of D_2 on Cu(111) for which results were available from another group [50]. This was possible because the v_0, α and T_n parameters describing the D_2 + Cu(111) experiments of Michelsen et al. [50] were available directly from one of the authors and could be used in the simulations [3]. Calculations using the SRP-DF and these parameters reproduced the D_2 + Cu(111) experiments [50] with chemical accuracy [3]. Subsequently, calculations using the same DF, and the parameters presented in Berger's thesis describing the pure beam H_2 + Cu(111) sticking experiments presented in the thesis (Fig.5.9 thereof) as well as in Ref. [49] (figure 3 thereof) also described these sticking experiments with chemical accuracy. It is very unlikely that this could have been done with the wrong parameters describing pure H_2 beams in the H_2 + Cu(111) experiments of Refs. [48-49]: Sticking probabilities for pure beams of H_2 on Cu(111) measured by Rettner et al. [51] were shifted to higher average energies by 1-2 kcal/mol, and could only be described with chemical accuracy if the appropriate molecular beam parameters describing these experiments using much narrower beams (in velocity) were used in the simulation of these experiments [3].

The calculation of the sticking probability then proceeds as follows. In the simulation of a molecular beam sticking experiment, for each trajectory the velocity towards the surface is generated in a random fashion according to the velocity distribution of Eq.S10, and the v_0 and α parameters describing that distribution. Likewise, the initial vibrational state associated with the

molecule, and the rotational state are selected according to the Boltzmann distribution with the vibrational temperature equal to the value of T_n, and the rotational temperature equal to 0.8 T_n, using the appropriate value of the nozzle temperature describing the experiment, as is normally done in simulating molecular beam experiments using pure H_2 beams [3, 5, 8, 45]. In sampling the velocity, a maximum is set on the velocity that corresponds to an initial translational energy of 5 eV. In sampling the initial vibrational quantum number v and the initial rotational quantum number j, maximum values of v_{max} = 5 and j_{max} = 50 are imposed in all simulations. The other coordinates and (angular) velocities of the molecule are sampled as described in Refs.[8, 52], except that here we also randomly sample the coordinates and the velocities of the surface atoms as described above in Section S6.1. The procedure we use corresponds to the full Monte-Carlo (FMC) averaging procedure described in Ref.[8]. This procedure constitutes the most efficient way to simulate a molecular beam sticking experiment with quasi-classical mechanics, as the Monte-Carlo integration is performed over all variables at once.

S6.3 Classical trajectory simulations

The LAMPPS code [39-40] was used to perform quasi-classical trajectory (QCT) calculations [53-54] in NVE simulations. In these calculations a time step of 0.2 fs was employed. In order to compute sticking coefficients 10^6 trajectories were run for each simulated molecular beam experiment (see Section S6.2 above). In each trajectory the molecule starts at Z=8 A, i.e., at 8 Å from the surface. Trajectories are considered as resulting in reaction when the H-H distance becomes greater than 1.8 Å (this value may be compared to the maximum H-H distance found for the six barrier geometries in Table 1, which is 1.37 Å for the top site). Trajectories are considered as resulting in scattering when the molecule-surface distance exceeds 8 Å and the molecule's velocity points away from the surface. Trajectories are ended if the total propagation time exceeds 5 ps, but in practice this never happened. In view of the high minimum barrier in the HDNN PES (24.5 kcal/mol, see Table S7), a trajectory was only computed if the initial total

kinetic and internal potential energy of the molecule (translational + rotational + vibrational) exceeded 1.04 eV ≈ 24 kcal/mol, to save on computation time. This value may be compared to the lowest barrier height found for the HDNN fit of the QMC-DFT data at 220 K, which was 24.8 kcal/mol (see Table S1). If not computed due to the total molecular energy not exceeding this threshold, the trajectory was labeled as "not reacted". Elaborate checks were performed to ensure that this did not affect the accuracy of the computed sticking probabilities (i.e., that none of the "non-computed trajectories" would have resulted in reaction).

S7. Molecular dynamics with electronic friction (MDEF) calculations

S7.1 Calculation of the friction coefficients with the local density friction approximation (LDFA)

In MDEF we numerically integrate the classical generalized Langevin equation for the molecular degrees of freedom $\boldsymbol{q} = (\boldsymbol{q}^1, \boldsymbol{q}^2)$ according to

$$m\frac{d^2q^i}{dt^2} = -\frac{dV(\boldsymbol{q},\boldsymbol{Q})}{dq^i} + \eta(r_s(\boldsymbol{q}^i))\frac{dq^i}{dt} + F_{rand}(\eta(r_s(\boldsymbol{q}^i)),T_s) \qquad \text{(S11)}$$

The electronic friction coefficients η are evaluated in the local density friction approximation (LDFA) [7] as described in detail in Ref.[55], using the non-spin-polarized local density approximation for the underlying atom-in-jellium model. In the above equation, q^i is a Cartesian coordinate of H-atom i, and $\boldsymbol{q}$ is the vector of the Cartesian coordinates of the two H-atoms. Furthermore, $\boldsymbol{Q}$ is the vector of the surface atom coordinates, and V is the potential energy surface. As is usual in MDEF calculations on molecules scattering from metal surfaces, the electronic friction only acts on the atoms making up the incident molecule [56].

The atomic friction coefficient in Eq.S11 depend on the so-called Wigner-Seitz radius r_s at the location of the atom, which is related to the electronic density ρ_0 at the location of the atom through

$$\left[r_s(\boldsymbol{q}^i)\right] = \left[\sqrt[3]{\frac{4}{3}\pi\rho_0(\boldsymbol{q}^i)}\right]^{-1} \qquad \text{(S12).}$$

For H atoms, the dependence of the atomic friction coefficients on r_s can be fit quite well to the expression

$$\eta(r_s) = Ar_s^B \exp(-Cr_s) \qquad \text{(S13).}$$

In Eq.S13, $A = 0.6469\,\hbar/a_0^{B+2}$, B = 0.3062, and C = 0.5676 a_0^{-1}. The quality of the fit is shown in Fig.S5.

The calculation of the friction coefficient in the LDFA requires the calculation of the electronic density ρ_0 as a function of the metal atom coordinates in the absence of the molecule [7]. This electron density is related to r_s through Eq.S12, which is used to compute η with Eq. S13. The resulting friction coefficients are fitted with a frozen-surface neural network approach [57] as in previous work [58-60]. Using the friction coefficients for H_2 interacting with a mobile metal surface should be a good approximation at low surface temperatures (as the one used here, 220 K) at which the surface is not very distorted [61-62].

To put the values obtained for the friction coefficient for H_2 + Al(110) in perspective the friction coefficients η_{ZZ} and η_{rr} for molecular motion in Z and r, respectively, are compared to values calculated for H_2 + Cu(111) [58] and N_2 + Ru(0001) [59] in the upper and lower panels of Fig.S6. These molecular friction coefficients can be obtained from the LDFA value of η for the corresponding atom as described in for instance Ref.[58]. As can be seen from Fig.S6, when plotted

as a function of the reaction coordinate the molecular friction coefficients for H_2 + Al(110) are quite similar to those for H_2 + Cu(111), but much smaller than those found for N_2 + Ru(0001).

S7.2 Integration of the MDEF equations of motion

In our previous work for N_2 on Ru(0001) employing LDFA-based MDEF using a HD-NNP PES [60], we had implemented and used the MD propagation algorithm suggested by Ermak and Buckholz (EB) [63] for the (stochastic) integration of the Langevin equation, which represents a well-established and hence very common choice. In that work we had also considered the energy exchanged with electron-hole pairs during each MD time step, $\Omega(t)$. This can be added to the "conventional" NVE total energy $E_{\mathrm{tot}}(t) = E_{\mathrm{kin}}(t) + E_{\mathrm{pot}}(t)$, whereby $E_{\mathrm{pot}}(t)$ describes the contribution from the Born-Oppenheimer PES. Together both yield an effective conserved quantity $E_{\mathrm{eff}}(t) = E_{\mathrm{tot}}(t) + \Omega(t)$, which can be monitored during the stochastic MD simulations based on the Langevin equation (Eq. S11) to detect whether the chosen MD time step is too large.

Using the implementation from Ref. [60] for H_2 on Al(110), at an electronic temperature T_{el} = 0 K we obtain a numerical conservation of $E_{\mathrm{eff}}(t)$ that is of the same quality as $E_{\mathrm{tot}}(t)$ alone in MD simulations without electronic friction and the same integration settings. At T_{el} = 0 K the random force in Eq.S11 vanishes entirely, but as demonstrated recently by Kandratsenka and coworkers for the dynamics of H_2 on metal surfaces [64], this force must not be neglected for $T_{\mathrm{el}} >$ 0 K. Unfortunately, using the same computational settings as before at T_{el} = 220 K, we find that the EB integrator fails to conserve $E_{\mathrm{eff}}(t)$, with deviations occurring of about ±50 meV for both scattered and reacted trajectories (see Fig.S7 for the results of a reacted trajectory). We also note that the trajectories computed for time steps of 0.01 and 0.02 fs clearly appear to be subject not just to oscillations in $E_{\mathrm{eff}}(t)$, but also to a drift in $E_{\mathrm{eff}}(t)$, which is highly undesirable (Fig.S7).

It is not surprising that we had not noted a similar problem in our previous work on N_2 + Ru(0001) [60]. While friction coefficients obtained within the LDFA are at most a factor of two

higher for N_2 on Ru(0001) than for H_2 on Al(110) the random force scales inversely with the mass. That means that even though the electronic temperature was higher by about a factor 2.5 in our calculations on N_2 + Ru(0001), a significant dependence of the average energy loss due to ehp excitation ($\Omega(t)$) on the integration algorithm used did not arise in the calculations of Ref.[60].

In view of the much lighter molecular mass in the system addressed here and of the accuracy requirements on our results, the integrator recently devised by Grønbech-Jensen and Farago (GJF) [65-66] is a much better choice for H_2 + Al(110). Unlike the EB integrator, the GJF integrator is in velocity-Verlet form, which makes it much easier to obtain $E_{\mathrm{eff}}(t)$, both conceptually and in practice. We modified the GJF implementation recently added to the LAMMPS code [67] to use our LDFA-based electronic friction coefficients. For comparable MDEF trajectories our implementation conserves $E_{\mathrm{eff}}(t)$ to within fluctuations of a few meV at T_{el} = 220 K, with the same quality as $E_{\mathrm{tot}}(t)$ alone in both scattered and reacted MD trajectories without electronic friction. This can be seen from Fig.S8, which gives results for a reacted trajectory propagated from the same initial conditions as the trajectory shown in Fig.S7. The fluctuations in $E_{\mathrm{eff}}(t)$ are much smaller in Fig.S8 than in Fig.S7 (note the order of magnitude difference of the scale on the y-axis in the figures), and no energy drifts are visible in the results of the new integrator.

S7.3 MDEF simulations and the sampling of the initial conditions

In the MDEF simulations performed in the framework of the NBOMS model the initial conditions are identical to those of the QCT calculations performed within the BOMS model. This was done on purpose, so that differences between the outcomes of the BOMS-QCT calculations and the MDEF calculations can be attributed as much as possible to the effect of ehp excitation. The same is true for the QCT calculations performed with the surface held static using the BOSS model; here the initial conditions are likewise the same as in the BOMS QCT calculations, except that the surface atoms are at all times held in their ideal lattice positions.

The only difference between the BOMS-QCT and the MDEF calculations is that the latter use a smaller time step (0.1 fs instead of 0.2 fs).

S8. Quantum corrections to the NBOMS sticking probabilities

In the main paper quantum corrections to the sticking probabilities computed with the NBOMS model have been presented. For a full understanding of how this was done, we briefly summarize the approach taken. In Ref.[8], QCT and QD calculations on H_2 + Al(110) were done with the BOSS model, which were based on DFT calculations for this system with the surface at 200 K in exactly the same way as described in Section 4 above. The same QMC-DF as described in Section S3 was used.

Both the QCT and the QD calculations of Ref.[8] used a different fit of the QMC-DFT calculations than the HDNNP fit used here. This was done for two reasons. First of all, QD calculations require the PES to be available on a large grid of points, with the details of the grid as well as its size depending on the input parameters to the QD calculations. Because the HDNNP is more expensive to evaluate per point than the method described below this can be cumbersome, especially at the stage where convergence tests are done for the QD calculations. Secondly, while QCT calculations need the PES to be available for the regions of the PES accessed by the dynamics, in QD the PES needs to be available on a direct product grid of points, i.e., also in regions of the PES that are not accessible classically. This puts much higher demands on the HDNNP method, as neural network methods tend to perform badly at extrapolation and the HDNNP fit performed here depended on corrections made with dynamics calculations at the later stage of making the fit. PESs for dissociative chemisorption of H_2 on metal surfaces can be accurately described with the corrugation reducing procedure (CRP) [68], and this method was used in Ref.[8]. A brief description of the application of this method, and of how the accuracy of the fit compares to the HDNNP fit for H_2 interacting with the ideal Al(110) surface at 220 K, is presented in Section S8.1.

As already stated, the CRP PES was used in both QCT and QD calculations to compute sticking probabilities in Ref.[8]. Because the calculation of sticking probabilities requires averaging over many initial rovibrational states of H_2 and over a large range of incidence energies for an accurate comparison to the available experiments, Monte-Carlo averaging was done over the initial rovibrational states in both the QD and the QCT calculations. In Section 8.2 we provide a very brief summary of how this was done, and illustrate the accuracy of the method for the QCT calculations. In this Section we will also show that the QCT BOSS results obtained with the CRP PES and with the HDNNP are very similar. This is proof of the accuracy of both fitting methods for H_2 interacting with the ideal, static Al(110) surface held at 220 K. It also provides justification for the two ways in which we have added a quantum correction to the sticking probabilities computed with the NBOMS method, which are described in Section 8.3.

S8.1 CRP fit of the PES for H_2 interacting with an ideal static Al(110) surface at 220 K

The CRP is essentially a method for arriving at an accurate fit of the six-dimensional PES to data for the interaction of a diatomic molecule interacting with a static ideal surface. This is achieved by removing most of the corrugation and the anisotropy from the problem by describing these features in an approximate manner by fitting atom-surface potentials an subtracting these from the DFT data, leaving a much smoother six-dimensional function to be interpolated. The method was first described in Ref. [68] and its application to H_2 + Al(110) is fully described in Ref.[8]. Here we focus on the accuracy of the method and the comparison of the CRP fit to the HDNNP fit.

CRP energies are compared with QMC-DFT energies, and also with HDNNP and DMC energies, where all are computed for the DMC geometries that have been used as anchor points (see Section S2 and Table 1), in Table S7. Relative to the QMC-DFT data for the BG1-BG6 DMC geometries, the CRP fit displays a MSE of 0.3 kcal/mol, and because the CRP barrier heights systematically overestimate the QMC-DFT barrier heights, the MAE is likewise 0.3

kcal/mol (see Table S7). As discussed in Section S5, relative to the QMC-DFT data the HDNNP energies for the DMC barrier geometries exhibited a negative MSE, of -0.4 kcal/mol, and a MSE of 0.7 kcal/mol. From this point of view, the CRP fit is somewhat more accurate than the HDNNP fit of the QMC-DFT data. However, we note that both methods (rather) accurately describe the energy of the BG1 DMC geometry, which is shown to be most important in the main paper. Comparing the CRP with the HDNNP energies for the six DMC barrier geometries we find a MSE of -0.6 kcal/mol and a MAE of 0.6 kcal/mol, showing systematically lower HDNNP energies at the DMC barrier geometries than CRP energies.

The saddle points (in the reduced dimensionality as specified by the impact point and orientation characterizing BG1-BG6) in the CRP and HDNNP fits, and the saddle points in interpolated QMC-DFT data, do not completely coincide with the DMC geometries BG1-BG6. It is also useful to compare these saddle point energies, as they represent the actual barrier heights associated with the fits. Energies of saddle-points (in two dimensions (r and Z) for each X,Y, θ, and φ) computed with QMC-DFT and the HDNNP and CRP PESs are compared to one another, and to the DMC energies at the DMC barrier geometries, in Table S8. Relative to the QMC-DFT saddle-point energies, the saddle point energy obtained with the CRP fit displays a MSE of 0.2 kcal/mol, and because the CRP systematically overestimates the QMC-DFT saddle point energies, the MAE is likewise 0.2 kcal/mol (see Table S8). The saddle point energies obtained with the CRP are therefore somewhat too high, but only by a small amount. Relative to the QMC-DFT values, the HDNNP saddle point energies exhibit a negative MSE, of -0.3 kcal/mol, and a MAE of 0.7 kcal/mol. However, we note that both methods accurately describe the QMC-DFT saddle point energy of the BG1 geometry and the DMC energy of BG1, which is shown to be most important in the main paper. Comparing the CRP with the HDNNP energies for the six saddle points we find a MSE of -0.6 kcal/mol and a MAE of 0.6 kcal/mol, showing systematically lower HDNNP energies at the saddle points than CRP energies.

In conclusion, we find that both methods rather accurately describe the QMC-DFT energies obtained at the DMC barrier geometries, and the QMC-DFT saddle point energies obtained at the

saddle point geometries of each method. This is especially true for the BG1 geometry, which is shown to be most important for the sticking in the main paper. In the end, what counts most for the relative accuracy of the CRP and the HDNNP fits to the QMC-DFT data is whether their use in dynamics calculations leads to similar sticking probabilities. This point will be addressed below in Section S8.3.

S8.2 QCT and QD dynamics on the CRP PES

To enable quantum corrections to be carried out to the NBOMS sticking probabilities computed here, QD and QCT calculations were carried out [8]. The QD calculations employed the time-dependent wave packet (TDWP) method [69-70]. At energies low enough that entrance resonances affected the scattering of H_2 back to the gas phase, a flux analysis method [71-72] was used to compute sticking probabilities that are not affected by artifacts due to these resonances in the QD calculations, as fully discussed in Ref. [8]. A comparison of initial-state selected dissociative chemisorption probabilities computed for a particular vibrational quantum number v, rotational quantum number j, and magnetic rotational quantum number m_j, is provided in Fig.S9, for a v=0, 1, and 2 state. As can be seen the QD initial-state selected dissociative chemisorption probabilities tend to be lower than the QCT probabilities at the lowest incidence energies, which can be attributed to tunneling (Ref.[8], see also figure 4 thereof).

Because the velocity distributions used in the experiments tend to emphasize contributions to sticking at lower energies in Fig.S9 (see also figure 8 of Ref.[8]), the tunneling contribution to the sticking can be important. For this reason we have evaluated the difference between QD and QCT sticking probabilities, which involves taking the average over initial-state selected reaction probabilities such as the ones displayed in Fig.S9. As already noted above, for this a procedure for Monte-Carlo averaging over initial rovibrational states was used, called partial Monte-Carlo (PMC) averaging. In this procedure, 35 ($v, j, m_j > 0$) states where selected with equal likelihood among 319 states that were available after determining the maximum numbers needed of v and j

for convergence. As discussed in detail in Ref.[8], in the calculation of the sticking probabilities the results for these states were weighted according to their Boltzmann weights at the relevant nozzle temperature. However, picking these states with probabilities not depending on the initial rovibrational states is important to ensure that the most reactive states (with high v, which carry a high reactivity but a low Boltzmann weight) are represented. As Fig.S10 shows the PMC sticking probabilities are in excellent agreement with the most accurately calculated QCT sticking probabilities computed in Ref.[8], which were computed with a procedure called no Monte-Carlo averaging (NMC). In the NMC procedure, 500,000 trajectories were run for all 319 rovibrational states for a grid of incidence energies, and then averaged over all these states and the velocity distribution. As Fig.S10 also shows, both the PMC and the NMC QCT sticking probabilities computed with the CRP PES and within the BOSS model are in excellent agreement with the QCT sticking probabilities calculated with the FMC procedure mentioned in Section 6 (see also Ref.[8]) using the HDNNP and the same dynamical (BOSS) model. This shows that, when used in dynamics calculations within the same dynamical model, the CRP and the HDNNP fits lead to similar computed sticking probabilities, which is additional proof of the accuracy of both fitting methods, as had already been stated in Section S5.3.

8.3 Quantum corrections to the sticking probabilities computed with the NBOMS model

We now turn to the topic of how sticking probabilities computed with the NBOMS model, which takes into account the effects of phonon motion and of ehp excitation, can be approximately corrected for quantum effects on the basis of QD results that are available only for the BOSS model. Below we will use the symbol S_0 for sticking probabilities. Additionally we will describe the dynamical method, the procedure of averaging over rovibrational states, the dynamical model, and the fitting procedure used for the PES with 4 subsequent indices occurring in the superscript to the symbol for the sticking probability. Here, for the first index we use Q for QD, and C for classical (= quasi-classical). For the second index we use N for NMC, P for PMC,

and F for FMC to describe the procedure for averaging over rovibrational states. For the third index we use B for the BOSS model and N for the NBOMS model. For the fourth index we use C for the CRP method and H for the HDNN method for fitting a PES.

For the QCT sticking probabilities computed here with the FMC averaging procedure, the NBOMS model, and the HDNNP it is rigorously true that

$$S_0^{CFNH} = S_0^{CNBC} + (S_0^{CFNH} - S_0^{CNBC}) \qquad \text{(S14).}$$

To obtain Eq.S14 we have simply added and subtracted the sticking probability computed with the QCT method, the NMC averaging procedure, the BOSS model, and the CRP PES to and from the S_0^{CFNH}, respectively, to regain S_0^{CFNH}. We now rewrite the term on the right hand site using

$$\Delta = (S_0^{CFNH} - S_0^{CNBC}) \qquad \text{(S15),}$$

after which substitution in Eq.S14 yields

$$S_0^{CFNH} = S_0^{CNBC} + \Delta \qquad \text{(S16).}$$

From a physics point of view, the Δ term corrects the S_0^{CNBC} computed with the BOSS model for the effects of phonons and of ehp excitation. Through using S_0^{CFNH} this is done with a different averaging procedure over states and with a different potential, but in the above equations any effects arising from the different averaging procedure and potential are simply cancelled by the subtraction of S_0^{CNBC} to obtain Δ.

To apply a quantum correction to the sticking probabilities computed with the NBOMS model on the basis of the QD sticking probabilities obtained with the PMC averaging procedure, we now make two approximations. The first approximation is to replace S_0^{CNBC} on the rhs of

Eq.S16 with S_0^{QNBC}, i.e., with QD sticking probabilities computed for the BOSS model, with full summation over the rovibrational states (NMC) and using the CRP PES:

$$S_0^{QFNH} \approx S_0^{QNBC} + \Delta \qquad \text{(S17).}$$

The substitution made amounts to assuming that (i) all quantum effects are captured already with the BOSS model (which assumption is reasonable if extra quantum effects associated with the phonons and ehp excitations are less important), (ii) the FMC and NMC procedures yield similar results (which is confirmed by QCT results obtained with the FMC and NMC procedures for H_2 + Al(110) being indistinguishable to the eye, see figure S1 of Ref.[8]), and (iii) the CRP and HDNNP procedures yield similar sticking probabilities (which is confirmed by Fig.S10). Hence, the essential assumption here is that the quantum effects are already captured with the BOSS model.

The second approximation made is that, at the level of the BOSS model, the quantum correction can be computed with the PMC averaging procedure, so that

$$S_0^{QNBC} \approx S_0^{CNBC} + (S_0^{QPBC} - S_0^{CPBC}) \qquad \text{(S18).}$$

Equation S18 would hold rigorously (the "≈" could be replaced by the "=" sign) if the QD and QCT sticking probabilities would both have been computed at the NMC level, however, as discussed in Ref.[8] this would have been too expensive for the QD calculations. The use of Eq.S18 will give accurate results if (i) the PMC and NMC procedures yield similar results for the QCT sticking probabilities (as shown in Ref.[8]) and (ii) any errors remaining and resulting from the less accurate PMC procedure cancel between the QD and the QCT sticking probabilities in the term in brackets in Eq.S18, which seems a reasonable assumption to make. Assumption (i) also holds: In Ref. [8] it was shown that the QCT sticking probabilities computed with the PMC procedure differed from the NMC results by at most 16% for the average incidence energy of 6.0

kcal/mol, while the difference was 1.6 % for the value of 5.1 kcal/mol, and decreased monotonically from 7.4 to 1.6 % for values of the incidence energy increasing from 7.1 to 9.4 kcal/mol, as also investigated in the present work.

With the above equations and approximations, it is easy to show that the quantum-corrected NBOMS sticking probabilities can be computed with no additional approximations using

$$S_0^{QNNH} \approx S_0^{CFNH} + (S_0^{QPBC} - S_0^{CPBC}) \qquad \text{(S19).}$$

Using Eq.S19 amounts to obtaining quantum corrected NBOMS sticking probabilities with the HDNNP by adding the difference between QD and QCT sticking probabilities computed using the PMC averaging procedure, the BOSS model, and the CRP PES to NBOMS sticking probabilities computed with the MDEF method of Section 7 and with the HDNNP. We label the corresponding method for carrying out the quantum correction as procedure A.

To assess whether using a different procedure yields dramatically different quantum corrections to the NBOMS results we have also computed quantum corrected NBOMS results using

$$S_0^{QNNH} \approx S_0^{CFNH} + (S_0^{QPBH} - S_0^{CPBH}) \qquad \text{(S20).}$$

Like procedure A, Eq.S20 assumes that the quantum correction to the NBOMS sticking probabilities can be computed at the BOSS level. However, conceptually the quantum correction is now computed with the HDNNP. As the QD results were obtained with the CRP PES, in practice we compute the QD sticking probabilities using

$$S_0^{QPBH} \approx {S_0^{QPBC}}\Big/{S_0^{CPBC}}\, S_0^{CPBH} \qquad \text{(S21),}$$

leading to

$$S_0^{QNNH} \approx S_0^{CFNH} + \left(S_0^{QPBC} \middle/ S_0^{CPBC} \, S_0^{CPBH} - S_0^{CPBH} \right) \quad \text{(S22).}$$

The use of Eq.S22 constitutes procedure B for the quantum correction of the NBOMS sticking probabilities. This procedure assumes, through Eq.S21, that the ratio of the QD and the QCT sticking probabilities computed with the CRP PES is equal to the ratio of these sticking probabilities computed with the HDNNP, which seems likely in view of the agreement noted between QCT-BOSS sticking probabilities computed with the CRP PES and the HDNNP in Fig.S10. The comparison of the outcomes of using procedures A and B shows little difference: with procedure A the quantum correction shifts the sticking curve to lower energies by 0.18 kcal/mol (Fig.2A), while with procedure B the sticking curve is shifted to lower energies by 0.23 kcal/mol (Fig.S11). Most importantly, which way the quantum correction is applied makes little difference, and in the main paper we have preferred to use the simpler method (procedure A).

S9. Importance of the use of correct velocity distributions

As Figure S12 shows, it is important to use the correct velocity distributions in the simulations. Sticking probabilities computed with the much narrower velocity distributions of RMA show misleadingly good agreement with experiment, but are shifted relative to results computed with the correct velocity distributions (i.e., of Berger et al.) by more than 1 kcal/mol.

Supporting references.

1. Perdew, J. P.; Burke, K.; Ernzerhof, M. Generalized gradient approximation made simple. *Phys.Rev.Lett.* **1996**, *77*, 3865-3868.
2. Powell, A. D.; Kroes, G. J.; Doblhoff-Dier, K. Quantum Monte Carlo calculations on dissociative chemisorption of H_2 on Al(110): Minimum barrier heights and their comparison to DFT values. *J.Chem.Phys.* **2020**, *153*, 224701.
3. Díaz, C.; Pijper, E.; Olsen, R. A.; Busnengo, H. F.; Auerbach, D. J.; Kroes, G. J. Chemically accurate simulation of a prototypical surface reaction: H_2 dissociation on Cu(111). *Science* **2009**, *326*, 832-834.
4. Nattino, F.; Migliorini, D.; Kroes, G. J.; Dombrowski, E.; High, E. A.; Killelea, D. R.; Utz, A. L. Chemically accurate simulation of a polyatomic molecule-metal surface reaction. *J.Phys.Chem.Lett.* **2016**, *7*, 2402-2406.
5. Kroes, G. J. Computational approaches to dissociative chemisorption on metals: Towards chemical accuracy. *Phys.Chem.Chem.Phys.* **2021**, *23*, 8962-9048.
6. Berger, H. F.; Rendulic, K. D. An investigation of vibrationally assisted dissociation: the cases H_2/Cu(110) and H_2/Al(110) *Surf.Sci.* **1991**, *253*, 325-333.
7. Juaristi, J. I.; Alducin, M.; Díez Muiño, R.; Busnengo, H. F.; Salin, A. Role of electron-hole pair excitations in the dissociative adsorption of diatomic molecules on metal surfaces. *Phys.Rev.Lett.* **2008**, *100*, 116102.
8. Tchakoua, T.; Powell, A. D.; Gerrits, N.; Somers, M. F.; Doblhoff-Dier, K.; Busnengo, H. F.; Kroes, G. J. Simulating highly activated sticking of H_2 on Al(110): Quantum versus quasi-classical dynamics. *J. Phys. Chem. C* **2023**, *127*, 5395-5407.
9. Hohenberg, P.; Kohn, W. Inhomogeneous Electron Gas. *Phys.Rev.* **1964**, *136*, B864-B871.
10. Kohn, W.; Sham, L. J. Self-Consistent Equations Including Exchange and Correlation. *Phys.Rev.* **1965**, *140*, A1133-A1138.
11. Kresse, G.; Furthmüller, J. Efficient iterative schemes for ab initio total-energy calculations using a plane-wave basis set. *Phys.Rev.B* **1996**, *54*, 11169-11186.
12. Kresse, G.; Joubert, D. From ultrasoft pseudopotentials to the projector augmented-wave method. *Phys.Rev.B* **1999**, *59*, 1758-1775.
13. Xiao, P.; Sheppard, D.; Rogal, J.; Henkelman, G. Solid-state dimer method for calculating solid-solid phase transitions. *J.Chem.Phys.* **2014**, *140*, 174104.
14. Kästner, J.; Sherwood, P. Superlinearly converging dimer method for transition state search. *J.Chem.Phys.* **2008**, *128*, 014106.

15. Heyden, A.; Bell, A. T.; Keil, F. J. Efficient methods for finding transition states in chemical reactions: Comparison of improved dimer method and partitioned rational function optimization method. *J.Chem.Phys.* **2005,** *123*, 224101.
16. Henkelman, G.; Jónsson, H. A dimer method for finding saddle points on high dimensional potential surfaces using only first derivatives. *J.Chem.Phys.* **1999,** *111*, 7010-7022.
17. Göbel, H.; von Blanckenhagen, P. Temperature-dependence of interlayer spacings and mean vibrational amplitudes at the Al(110) surface. *Phys. Rev. B* **1993,** *47*, 2378-2388.
18. Giannozzi, P.; Baroni, S.; Bonini, N.; Calandra, M.; Car, R.; Cavazzoni, C.; Ceresoli, D.; Chiarotti, G. L.; Cococcioni.; Dabo, I., et al. QUANTUM ESPRESSO: a modular and open source software project for quantum simulation of materials. *J.Phys.: Conden. Matter* **2009,** *21*, 395502.
19. Pandharipande, V. R.; Pieper, S. C.; Wiringa, R. B. Variational Monte Carlo calculations of ground states of liquid ^{4}He and ^{3}He drops. *Phys.Rev.B* **1986,** *34*, 4571-4582.
20. McMillan, W. L. Ground state of liquid ^{4}He. *Phys.Rev.* **1965,** *138*, A442-A451.
21. Foulkes, W. M. C.; Mitas, L.; Needs, R. J.; Rajagopal, G. Quantum Monte Carlo simulation of solids. *Rev. Mod. Phys.* **2001,** *73*, 33-83.
22. Kolorenc, J.; Mitas, L. Applications of quantum Monte Carlo methods in the condensed phase. *Rep.Prog.Phys.* **2011,** *74*, 026502.
23. Austin, B. M.; Zubarev, D. Y.; Lester, W. A., Jr. Quantum Monte Carlo and related approaches. *Chem.Rev.* **2012,** *112*, 263-288.
24. Umrigar, C. J.; Toulouse, J.; Filippi, C.; Sorella, S.; Hennig, R. G. Alleviation of the fermion-sign problem by optimization of many-body wave functions. *Phys. Rev. Lett.* **2007,** *98*, 110201.
25. Casula, M. Beyond the locality approximation in the standard diffusion Monte Carlo method. *Phys.Rev.B* **2006,** *74*, 161102.
26. Needs, R. J.; Towler, M. D.; Drummond, N. D.; López Ríos, P. Continuum variational and diffusion quantum Monte Carlo calculations. *J.Phys.:Condens.Matter* **2010,** *22*, 023201.
27. Needs, R. J.; Towler, M. D.; Drummond, N. D.; López Ríos, P.; Trail, J. R. Variational and diffusion quantum Monte Carlo calculations with the CASINO code. *J.Chem.Phys,.* **2020,** *152*, 154106.
28. Hammer, B.; Hansen, L. B.; Nørskov, J. K. Improved adsorption energetics within density-functional theory using revised Perdew-Burke-Ernzerhof Functionals. *Phys.Rev.B.* **1999,** *59*, 7413-7421.
29. Lee, K.; Murray, É. D.; Kong, L. Z.; Lundqvist, B. I.; Langreth, D. C. Higher-accuracy van der Waals density functional. *Phys.Rev.B* **2010,** *82*, 081101.

30. Dion, M.; Rydberg, H.; Schröder, E.; D.C. Langreth; Lundqvist, B. I. van der Waals density functional for general geometries. *Phys.Rev.Lett.* **2004,** *92*, 246401.
31. Behler, J.; Parrinello, M. Generalized neural-network representation of high-dimensional potential-energy surfaces. *Phys.Rev.Lett.* **2007,** *98*, 146401.
32. Kocer, E.; Ko, T. W.; Behler, J. Neural network potentials: A concise overview of methods. *Annu. Rev. Phys. Chem.* **2022,** *73*, 163-186.
33. Behler, J. Four generations of high-dimensional neural network potentials. *Chem.Rev.* **2021,** *121*, 10037-10072.
34. Behler, J. Atom-centered symmetry functions for constructing high-dimensional neural network potentials. *J.Chem.Phys.* **2011,** *134*, 074106.
35. Shakouri, K.; Behler, J.; Meyer, J.; Kroes, G. J. Accurate neural network description of surface phonons in reactive gas-surface dynamics: N_2 + Ru(0001). *J. Phys. Chem. Lett.* **2017,** *8*, 2131-2136.
36. Gerrits, N.; Shakouri, K.; Behler, J.; Kroes, G. J. Accurate probabilities for highly activated reaction of polyatomic molecules on surfaces using a high-dimensional neural network potential: CHD_3 + Cu(111). *J. Phys. Chem. Lett.* **2019,** *10*, 1763-1768.
37. Behler, J. Constructing high-dimensional neural network potentials: A tutorial review. *Int. J. Quantum Chem.* **2015,** *115*, 1032-1050.
38. Behler, J. First principles neural network potentials for reactive simulations of large molecular and condensed systems. *Angew.Chem.Int.Ed.* **2017,** *56*, 12828-12840.
39. Plimpton, S. Fast parallel algoirithms for short-range molecular dynamics. *J. Comput. Phys.* **1995,** *117*, 1-19.
40. Singraber, A.; Behler, J.; Dellago, C. Library-based LAMMPS implementation of high-dimensional neural network potentials. *J. Chem. Theory Comput.* **2019,** *15*, 1827-1840.
41. Andersson, S.; Persson, M.; Harris, J. Physisorption energies: influence of surface structure. *Surf.Sci.* **1996,** *360*, L499-L504.
42. Lee, K.; Kelkkanen, A. K.; Berland, K.; Andersson, S.; Langreth, D. C.; Schröder, E.; Lundqvist, B. I.; Hyldgaard, P. Evaluation of a density functional with account of van der Waals forces using experimental data of H_2 physisorption on Cu(111). *Phys.Rev.B* **2011,** *84*, 193408.
43. Tiwari, A. K.; Nave, S.; Jackson, B. The temperature dependence of methane dissociation on Ni(111) and Pt(111): Mixed-quantum classical studies of the lattice response. *J.Chem.Phys.* **2010,** *132*, 134702.
44. Tiwari, A. K.; Nave, S.; Jackson, B. Methane dissociation on Ni(111): A new understanding of the lattice effect. *Phys.Rev.Lett.* **2009,** *103*, 253201.

45. Kroes, G. J.; Díaz, C. Quantum and classical dynamics of reactive scattering of H_2 from metal surfaces. *Chem.Soc.Rev.* **2016**, *45*, 3658-3700.
46. Michelsen, H. A.; Auerbach, D. J. A critical examination of data on the dissociative adsorption and associative desorption of hydrogen at copper surfaces. *J.Chem.Phys.* **1991**, *94*, 7502-7520.
47. Auerbach, D. J. In *Atomic and Molecular Beam Methods*, Scoles, G., Ed. Oxford University Press: New York/Oxford, 1988; Vol. 1, pp 362-379.
48. Berger, H. F. Über den Einfluß des Quantenzustands auf die dissoziative Adsorption von Wasserstoff. Ph.D. thesis, Technische Universität Graz, Graz, 1992.
49. Berger, H. F.; Leisch, M.; Winkler, A.; Rendulic, K. D. A search for vibrational contributions to the activated adsorption of H_2 on copper. *Chem.Phys.Lett.* **1990**, *175*, 425-428.
50. Michelsen, H. A.; Rettner, C. T.; Auerbach, D. J.; Zare, R. N. Effect of rotation on the translational and vibrational energy dependence of the dissociative adsorption of D_2 on Cu(111). *J.Chem.Phys.* **1993**, *98*, 8294-8307.
51. Rettner, C. T.; Michelsen, H. A.; Auerbach, D. J. Quantum-state-specific dynamics of the dissociative adsorption and associative desorption of H_2 at a Cu(111) surface. *J.Chem.Phys.* **1995**, *102*, 4625-4641.
52. Wijzenbroek, M.; Helstone, D.; Meyer, J.; Kroes, G. J. Dynamics of H_2 dissociation on the close-packed (111) surface of the noblest metal: H_2 + Au(111). *J.Chem.Phys.* **2016**, *145*, 144701.
53. Karplus, M.; Porter, R. N.; Sharma, R. D. Exchange reactions with activation energy. I. Simple barrier potential for (H,H_2). *J.Chem.Phys.* **1965**, *43*, 3259-3287.
54. Porter, R. N.; Raff, L. M. Classical trajectory methods in molecular collisions. In *Dynamics of Molecular Collisions, Part B*, W.H.Miller, Ed. Plenum: New York, 1976; pp 1-52.
55. Gerrits, N.; Juaristi, J. I.; Meyer, J. Electronic friction coefficients from the atom-in-jellium model for Z=1-92. *Phys.Rev.B* **2020**, *102*, 155130.
56. Alducin, M.; Diéz Muiño, R.; Juaristi, J. I. Non-adiabatic effects in elementary reaction processes at metal surfaces. *Prog.Surf.Sci.* **2017**, *92*, 317-340.
57. Meyer, J. Ab initio modeling of energy dissipation during chemical reactions at transition metal surfaces. PhD Dissertation, FU Berlin, Berlin, 2012. http://dx.doi.org/10.17169/refubium-16459 (accessed 2023-12-20).
58. Spiering, P.; Meyer, J. Testing electronic friction models: vibrational de-excitation in scattering of H_2 and D_2 from Cu(111). *J. Phys. Chem. Lett.* **2018**, *9*, 1803-1808.
59. Spiering, P.; Shakouri, K.; Behler, J.; Kroes, G. J.; Meyer, J. Orbital-dependent electronic friction significantly affects the description of reactive scattering of N_2 from Ru(0001). *J. Phys. Chem. Lett.* **2019**, *10*, 2957-2962.

60. Shakouri, K.; Behler, J.; Meyer, J.; Kroes, G. J. Analysis of energy dissipation channels in a benchmark system of activated dissociation: N_2 on Ru(0001). *J. Phys. Chem. C* **2018**, *122*, 23470-23480.
61. Novko, D.; Blanco-Rey, M.; Alducin, M.; Juaristi, J. I. Surface electron density models for accurate ab initio molecular dynamics with electronic friction. *Phys.Rev.B* **2016**, *93*, 245435.
62. Blanco-Rey, M.; Juaristi, J. I.; Díez Muiño, R.; Busnengo, H. F.; Kroes, G. J.; Alducin, M. Electronic friction dominates hydrogen hot-atom relaxation on Pd(100). *Phys.Rev.Lett.* **2014**, *112*, 103203.
63. Ermak, D. L.; Buckholz, H. Numerical integration of the Langevin equation: Monte Carlo simulation. *J.Comp.Phys.* **1980**, *35*, 169-182.
64. Hertl, N.; Martin-Barrios, R.; Galparsoro, O.; Larrégaray, P.; Auerbach, D. J.; Schwarzer, D.; Wodtke, A. M.; Kandratsenka, A. Random Force in Molecular Dynamics with Electronic Friction. *J.Phys.Chem.C* **2021**, *125*, 14468–14473.
65. Grønbech-Jensen, N.; Farago, O. A simple and effective Verlet-type algorithm for simulating Langevin dynamics. *Mol. Phys.* **2013**, *111*, 983–991.
66. Grønbech-Jensen, N.; Hayre, N. R.; Farago, O. Application of the G-JF discrete-time thermostat for fast and accurate molecular simulations. *Comp. Phys. Commun.* **2014**, *185*, 524-527.
67. Grønbech-Jensen, N. Complete set of stochastic Verlet-type thermostats for correct Langevin simulations. *Mol. Phys.* **2020**, *118*, e1662506.
68. Busnengo, H. F.; Salin, A.; Dong, W. Representation of the 6D potential energy surface for a diatomic molecule near a solid surface. *J.Chem.Phys.* **2000**, *112*, 7641-7651.
69. Kosloff, R. Time-dependent quantum-mechanical methods for molecular dynamics. *J.Phys.Chem.* **1988**, *92*, 2087-2100.
70. Pijper, E.; Kroes, G. J.; Olsen, R. A.; Baerends, E. J. Reactive and diffractive scattering of H_2 from Pt(111) studied using a six-dimensional wave packet method. *J. Chem. Phys.* **2002**, *117*, 5885-5898.
71. Neuhauser, D.; Baer, M.; Judson, R. S.; Kouri, D. J. The application of time-dependent wavepacket methods to reactive scattering. *Comput.Phys.Commun.* **1991**, *63*, 460-481.
72. Zhang, D. H.; Zhang, J. Z. H. Full-dimensional time-dependent treatment for diatom-diatom reactions: The H_2 + OH reaction. *J.Chem.Phys.* **1994**, *101*, 1146-1156.

Supporting figures.

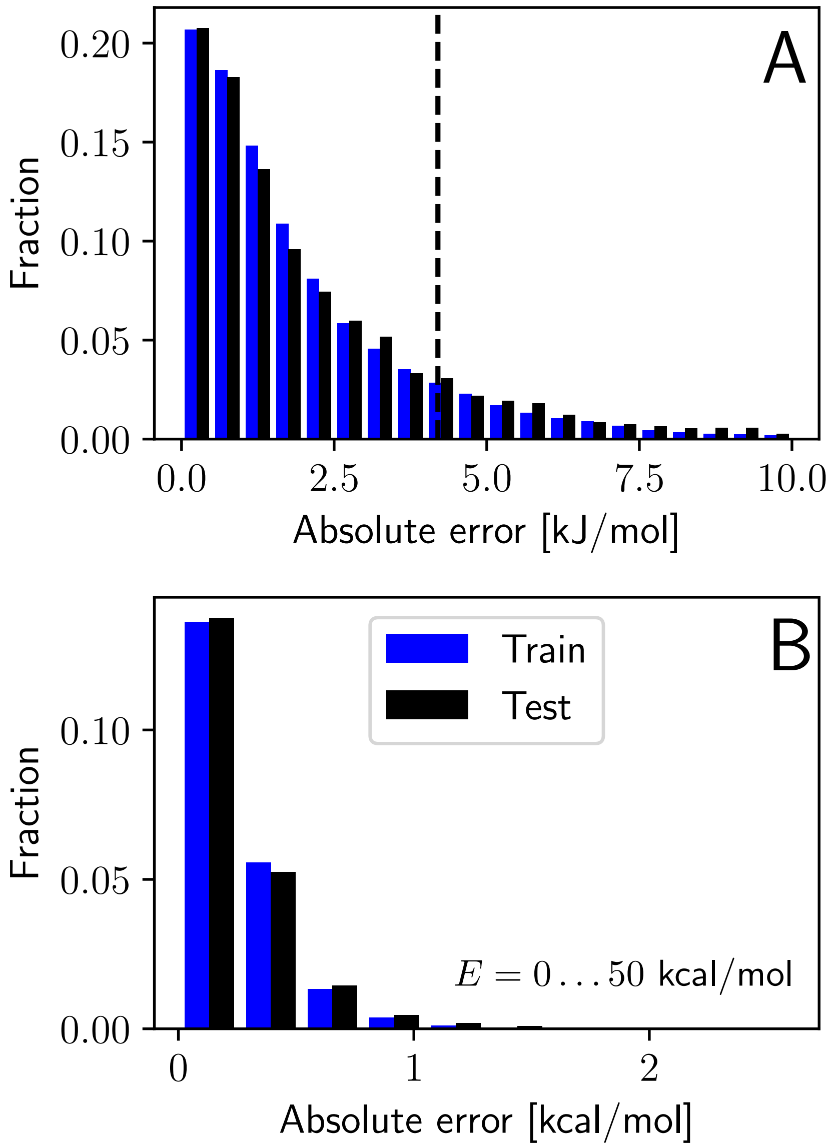

Figure S1. (A) For all QMC-DFT energies in the training and test data sets of the HDNNP for H_2 + Al(110) the fraction of these data exhibiting fitting errors in the specific ranges indicated on the x-axis are shown. The dashed vertical line indicates an error of 1 kcal/mol. (B) For QMC-DFT energies ≤ 50 kcal/mol in the training and tests data set of the HDNNP for H_2 + Al(110) the fraction of these data exhibiting fitting errors in the specific ranges indicated on the x-axis are shown. The zero of energy corresponds to the sum of the energies of H_2 in the gas phase and of the ideal 220 K Al(110) surface.

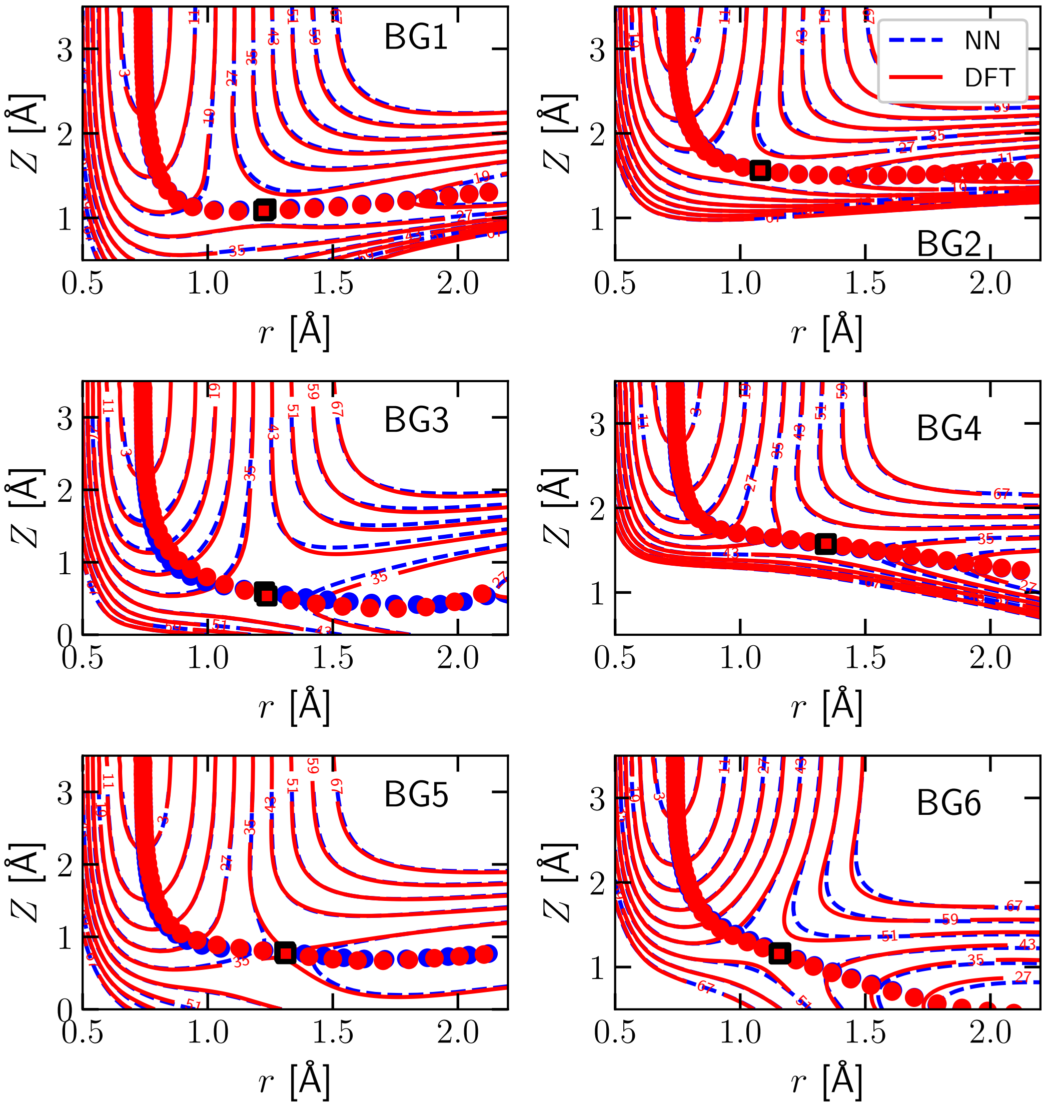

Figure S2. Contour plots (labeled by the energy relative to the minimum energy of the system consisting of H_2 in the gas phase and the Al(110) slab with all Al atoms in their equilibrium positions for the 220 K slab, in kcal/mol) are shown for six barrier geometries (see Fig.1); BG1 (top left), BG2 (top right), BG3 (middle left), BG4 (middle right), BG5 (bottom left), and BG6 (bottom right). Blue dashed lines (red full lines) show the results of the HDNN (raw QMC-DFT data). The big black (small red) square show the location of the barrier according to the HDNN fit (the raw QMC-DFT calculations).

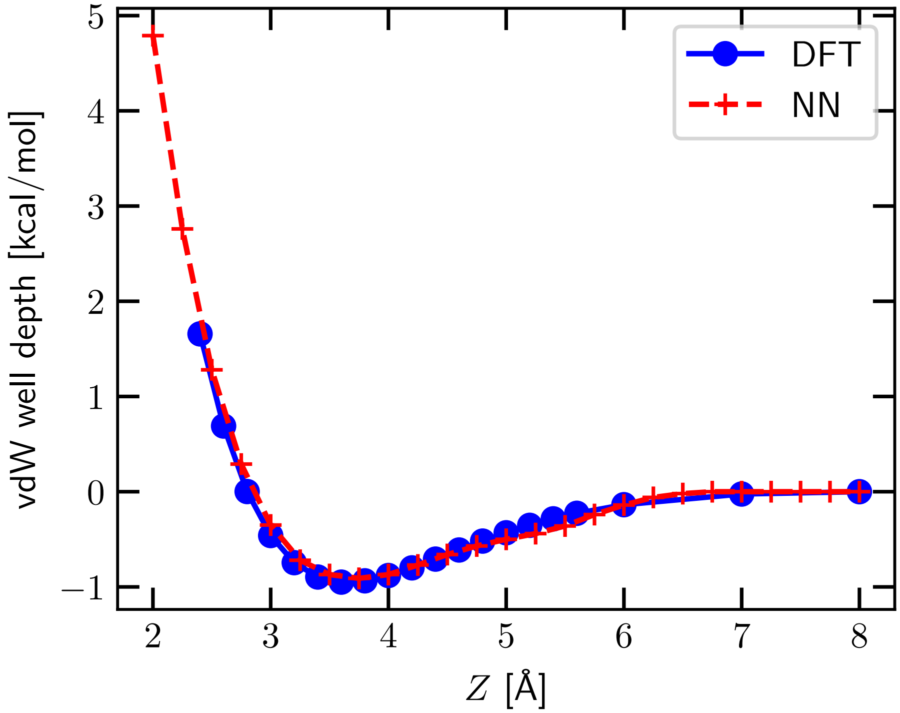

Figure S3. The HDNNP (red crosses, labeled NN), is compared to QMC-DFT data (blue circles) for H_2 parallel to the surface above the long bridge site, with $\phi = 0°$. The value of the H-H distance used in the calculations corresponds to the QMC-DFT value of the equilibrium distance of H_2 in the gas phase (0.741 Å).

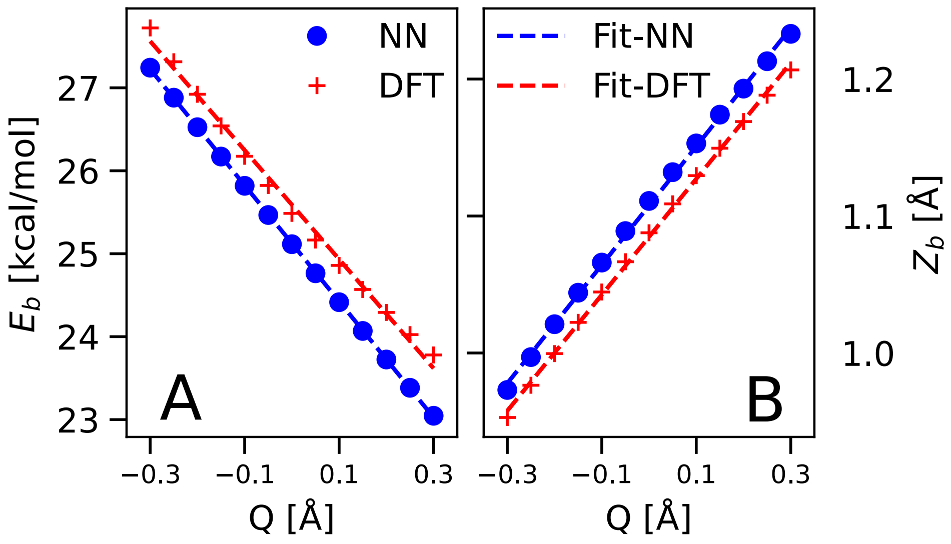

Figure S4. The height of the barrier associated with the BG1 barrier geometry is shown as a function of Q, which is the vertical displacement of one of the nearest neighbor top layer surface atoms to which the H-atoms dissociate (A). The location of the barrier associated with the BG1 barrier geometry is shown as a function of Q (B). Blue circles give results of the HDNN fit, and red crosses indicate raw QMC-DFT data.

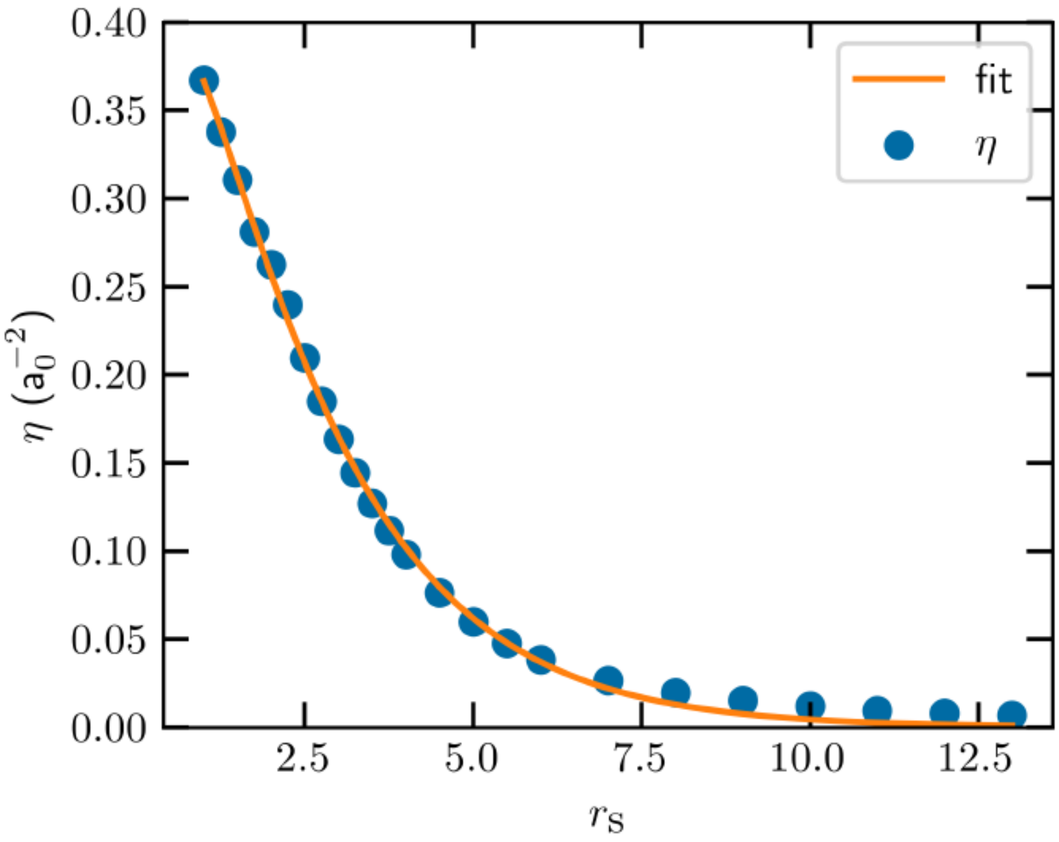

Figure S5. Electronic friction coefficient of a hydrogen atom as a function of r_s. The blue dots show the results from the calculations with the atom-in-jellium model and the orange line the least-square fit described by Eq.S13.

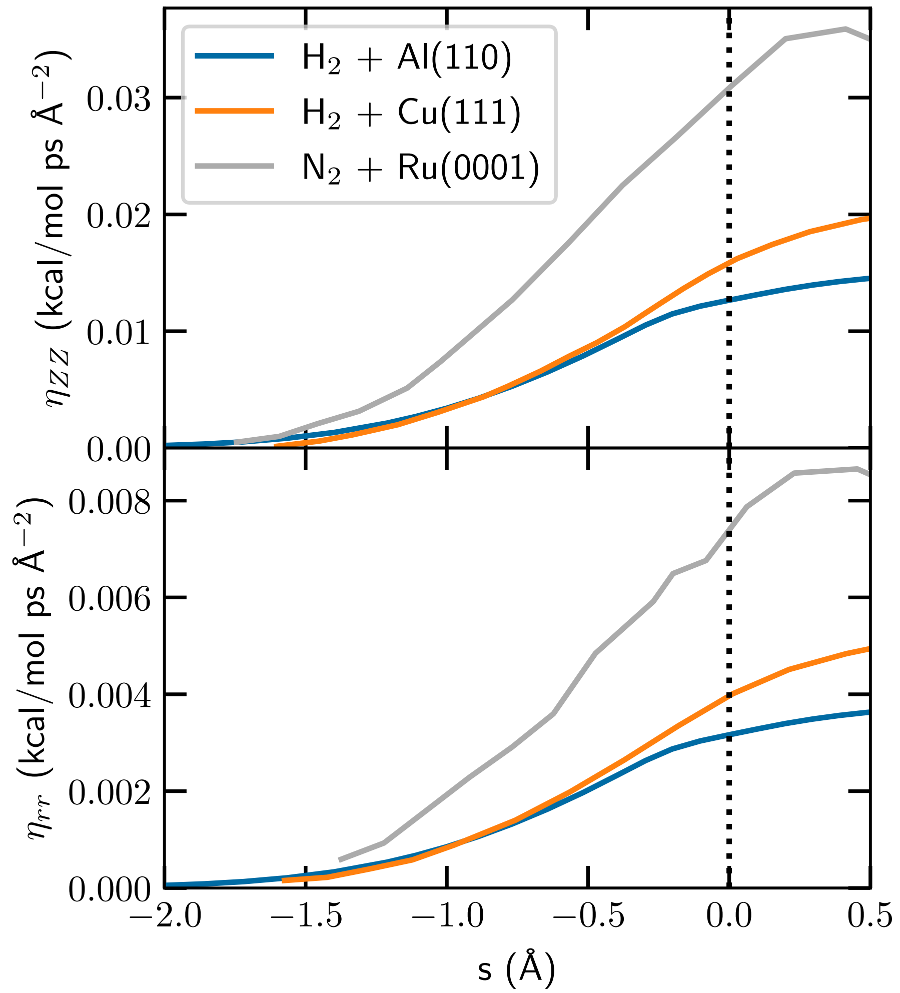

Figure S6. Electronic friction coefficient of H_2 + Al(110) (blue), H_2 + Cu(111) [58] (orange), and N_2 + Ru(0001) [59](grey) along the minimum energy path in the Z (upper panel) and r (lower panel) coordinates. The dashed vertical line at the reaction coordinate value s=0 indicates the position of the transition state.

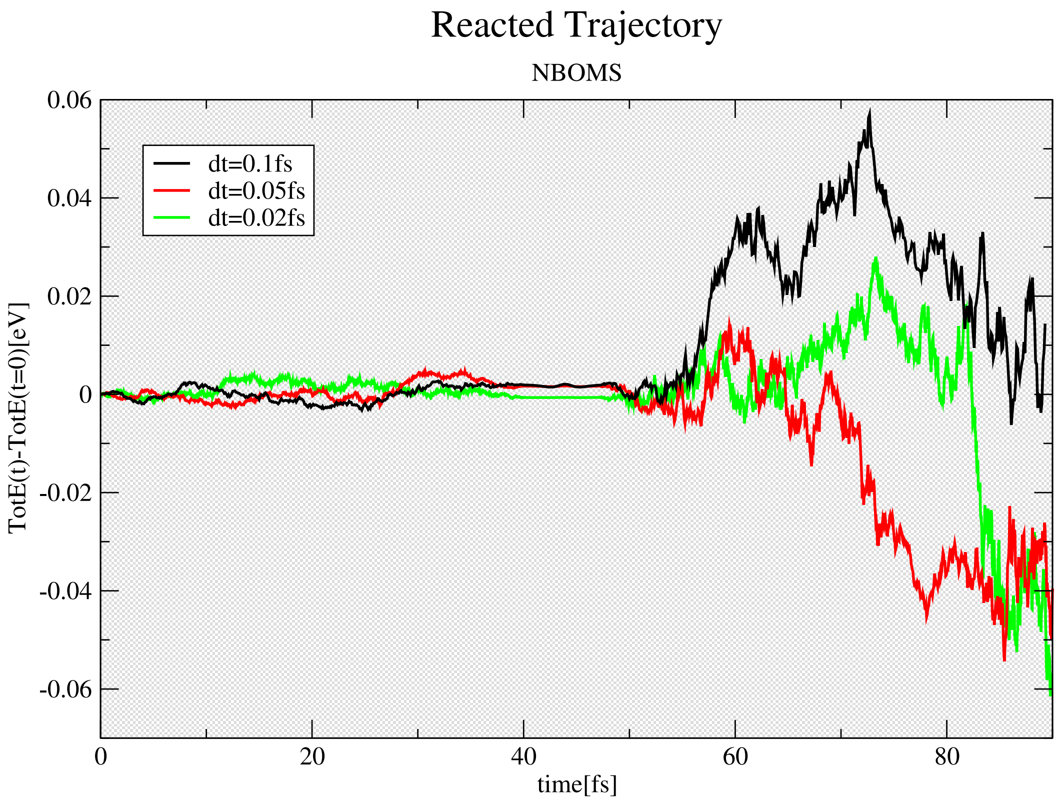

Figure S7. $E_{eff}(t)$, which should be an effectively conserved quantity, is shown as a function of time for a reactive trajectory computed with the MD propagation algorithm due to Ermak and Buckholz (EB) [63], for three different time steps dt.

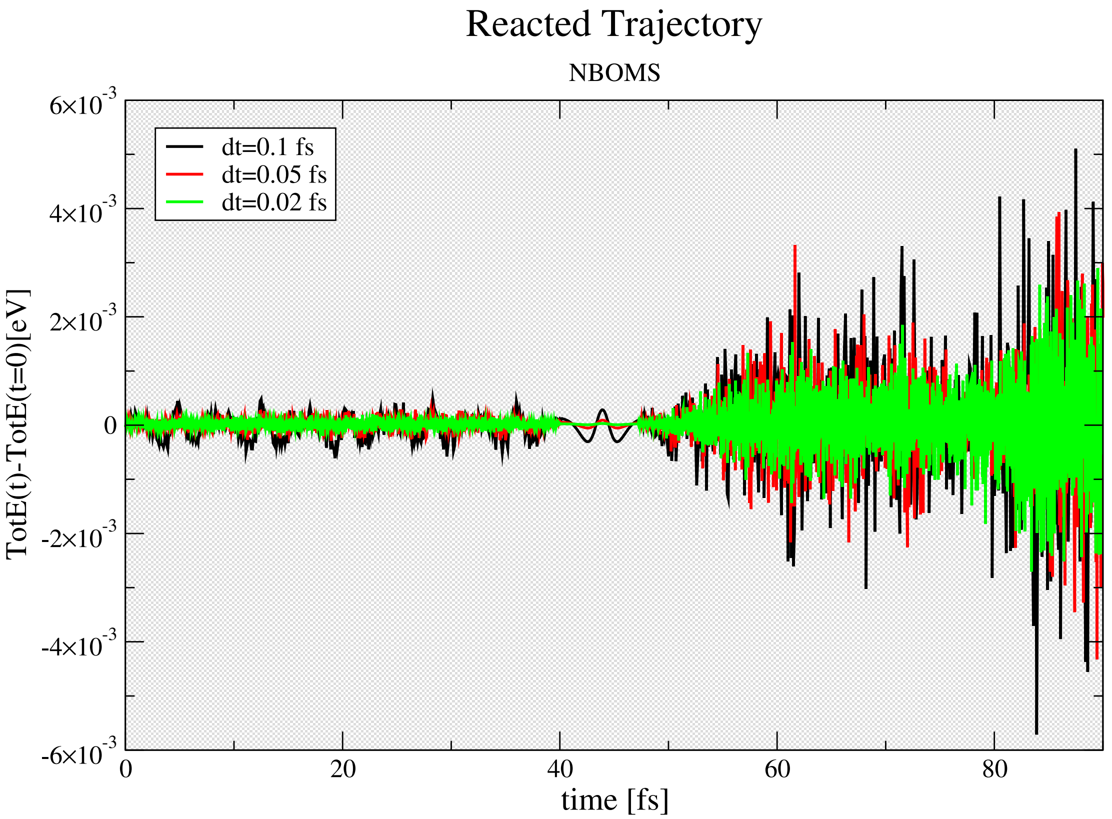

Figure S8. $E_{eff}(t)$, which should be an effectively conserved quantity, is shown as a function of time for a reactive trajectory computed with the MD propagation algorithm the integrator recently devised by Grønbech-Jensen and Farago (GJF) [65-66], for three different time steps dt.

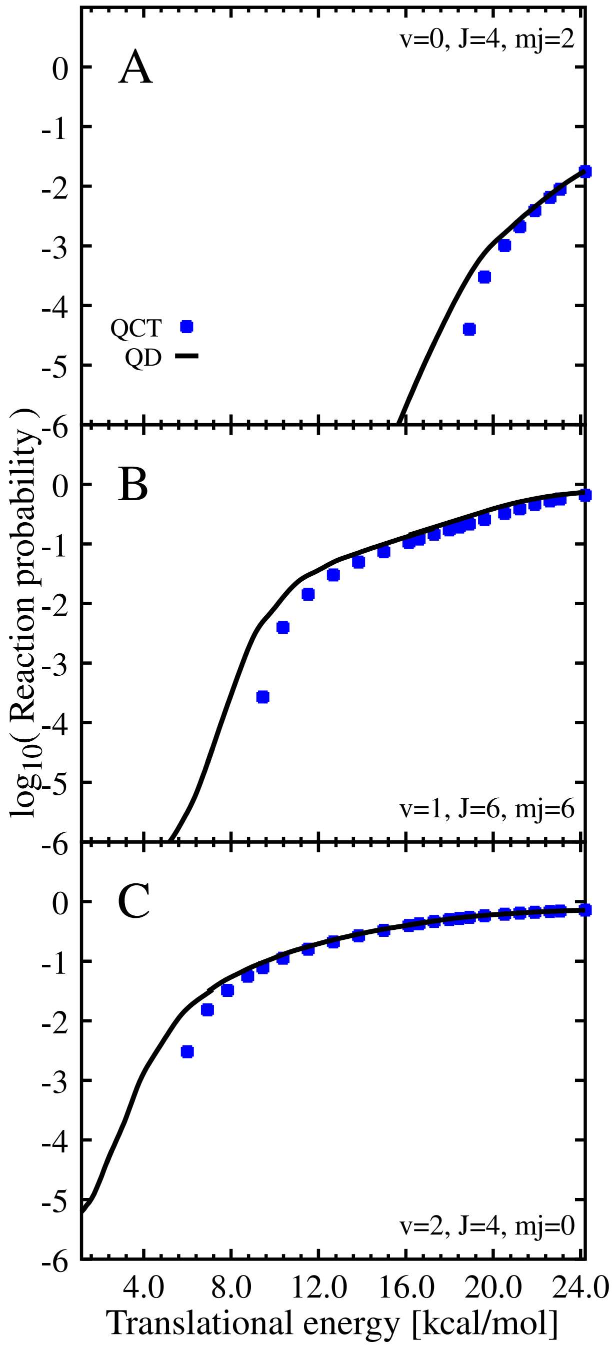

Figure S9. $R_{vjm_j}(E_i)$ computed with QD (black line) and QCT (blue squares) dynamics are compared for three different initial rovibrational states, of which (A) one with $v = 0$, (B) one with $v = 1$, and (C) one with $v = 2$.

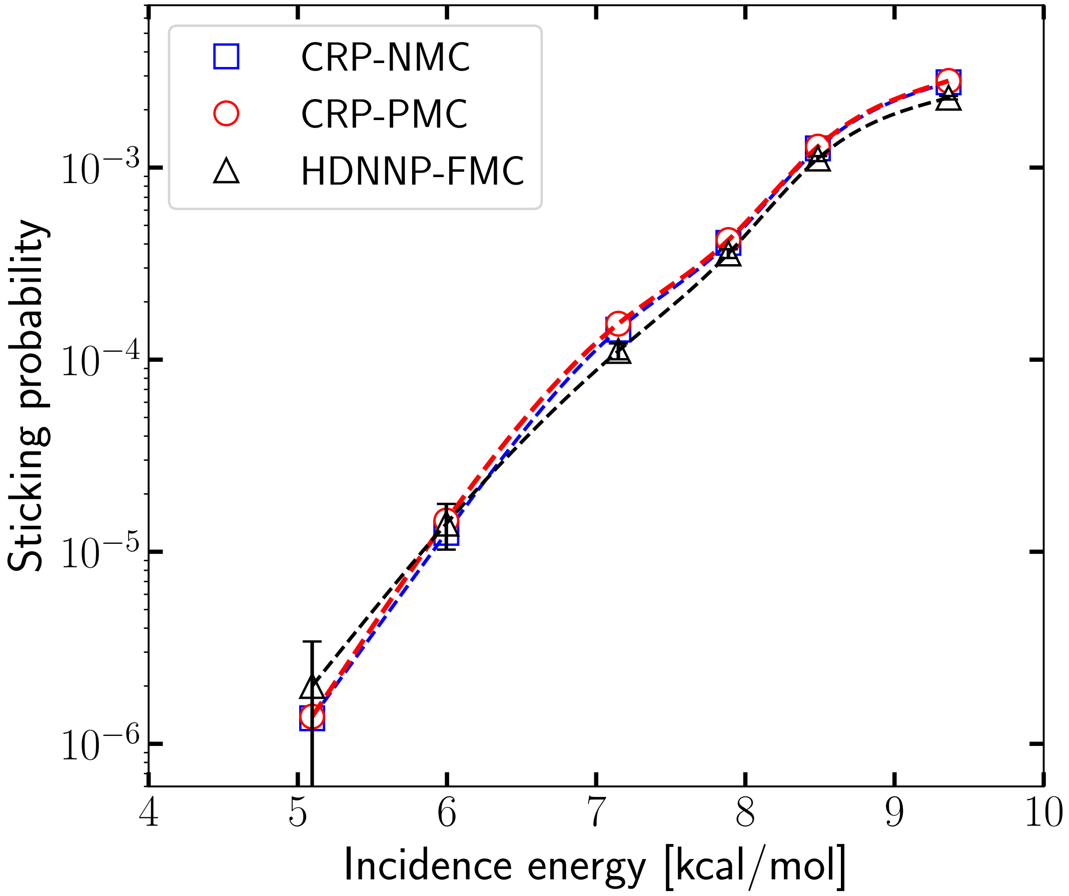

Figure S10. Sticking probabilities computed with the QCT method and the BOSS model and the CRP PES using the NMC and PMC averaging procedures (labeled CRP-NMC and CRP-PMC respectively) are compared to sticking probabilities computed using the same dynamical method and model, but using the FMC averaging procedure and the HDNNP (labeled HDNNP-FMC).

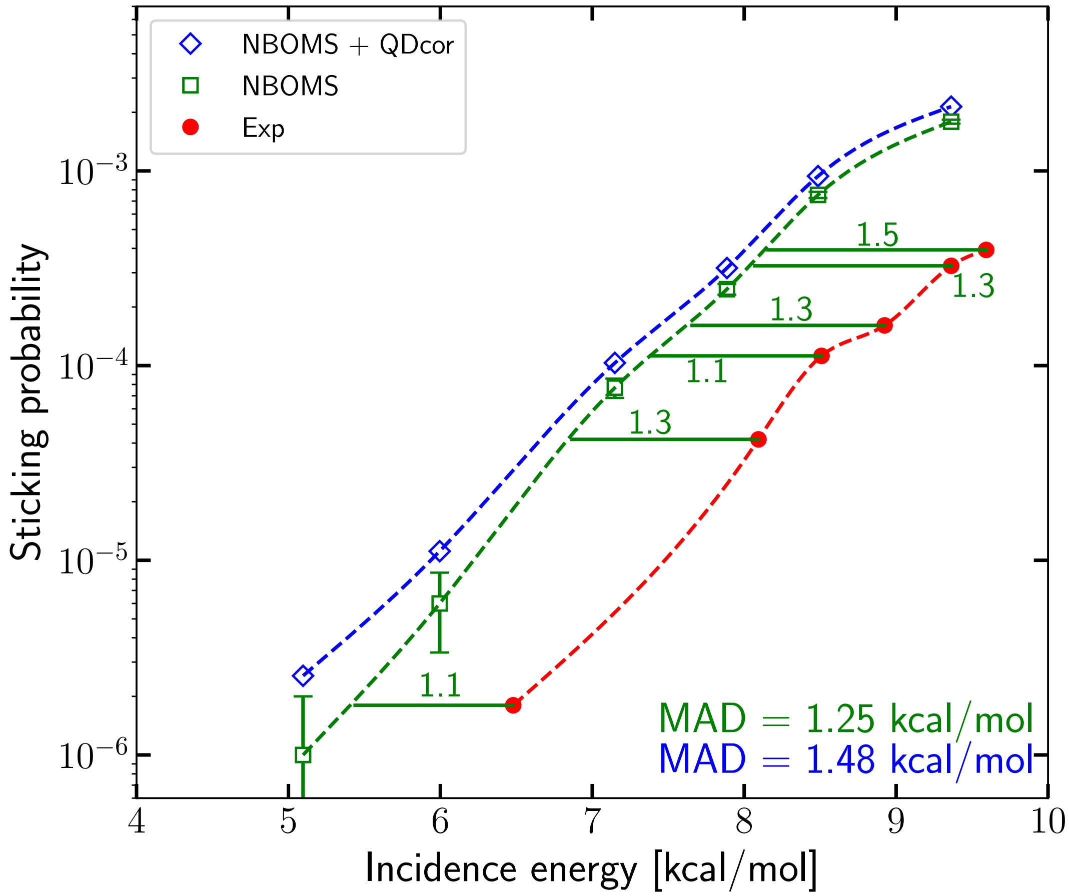

Figure S11. S_0 computed with the NBOMS model, and as computed with the NBOMS model but also corrected for quantum dynamical effects using procedure B of Section S8.3, are compared to experimental values.

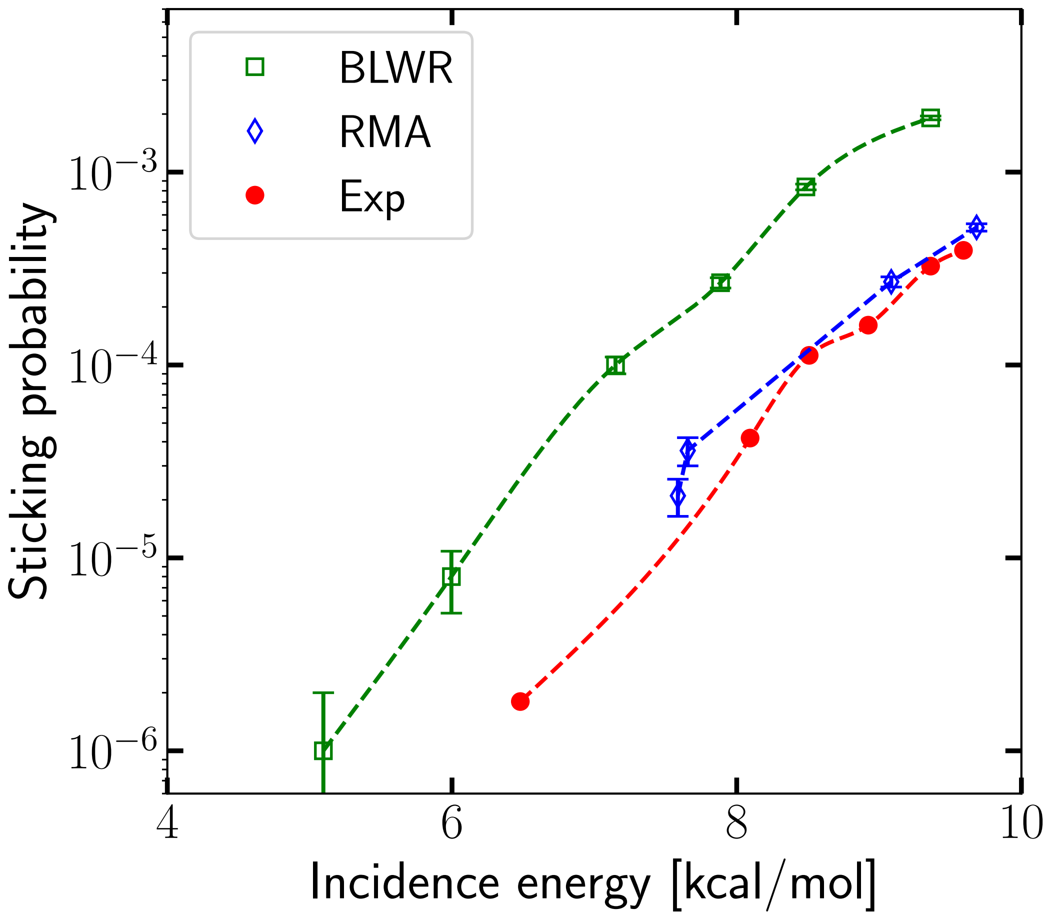

Figure S12. Sticking probabilities computed with the velocity distributions of Berger et al. (BLWR) are compared to sticking probabilities computed with the velocity distributions of Rettner et al. (RMA).

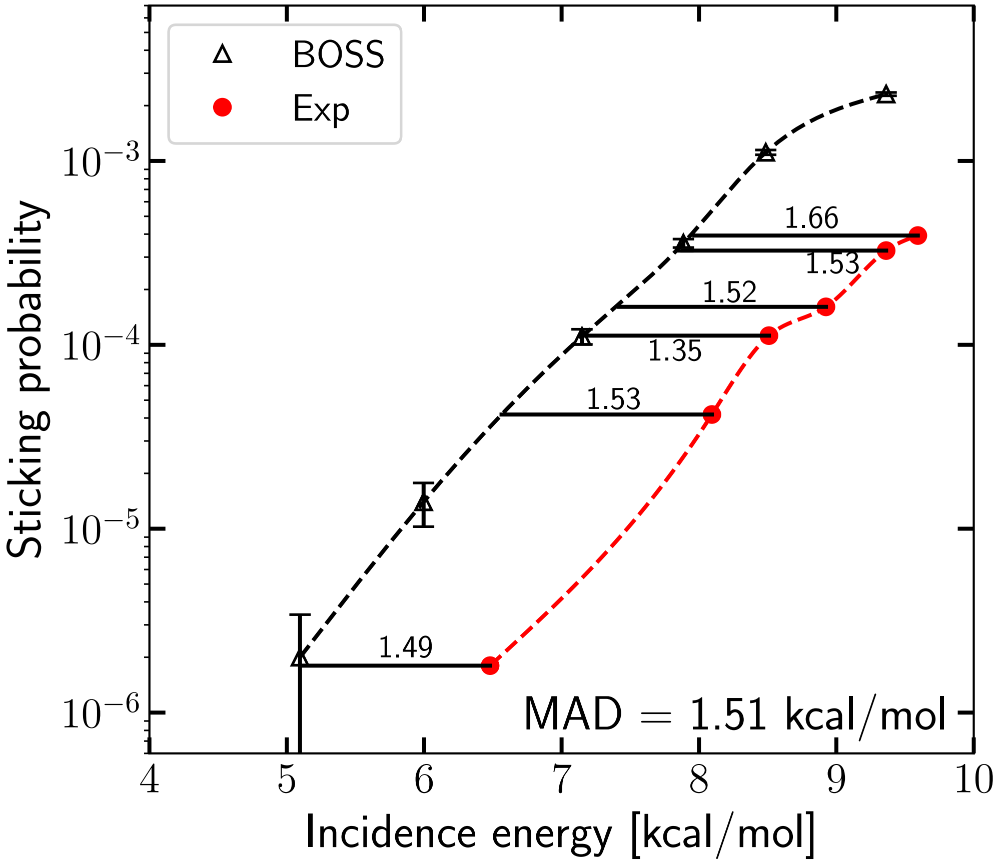

Figure S13. Sticking probabilities computed with the BOSS model using the HDNNP are compared to measured sticking probabilities for H_2+ Al(110) [6,48].

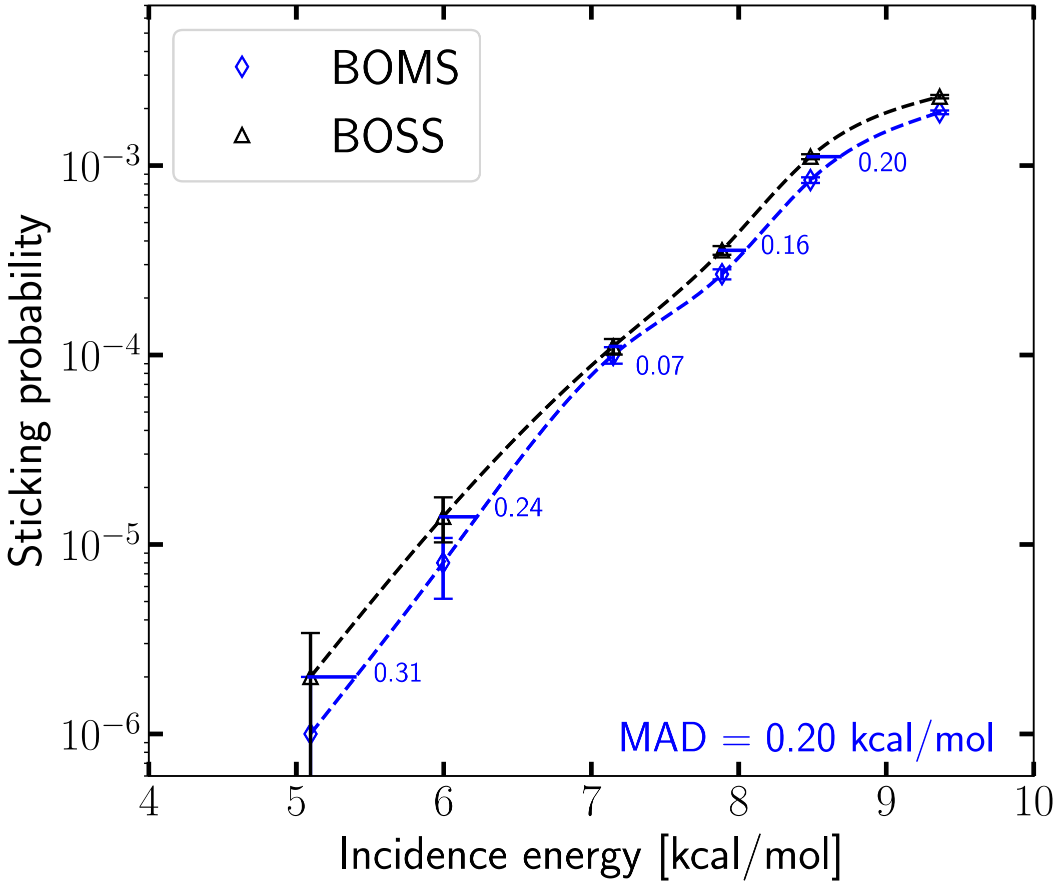

Fig.S14. Sticking probabilities computed for H_2 + Al(110) using the HDNN PES with the BOSS and the BOMS model are compared with one another, showing the effect of allowing surface atom motion on the sticking probability at T_s = 220 K.

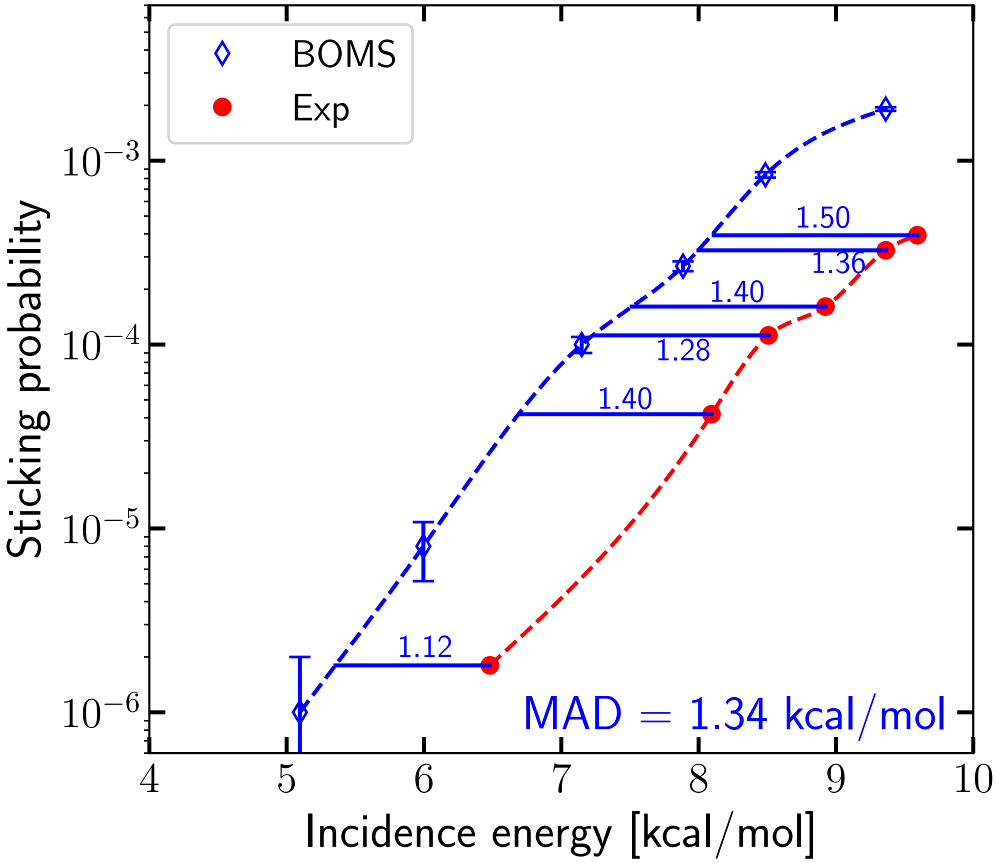

Figure S15. Sticking probabilities computed with the BOMS model and the HDNNP are compared to measured sticking probabilities for H_2+ Al(110) [6,48].

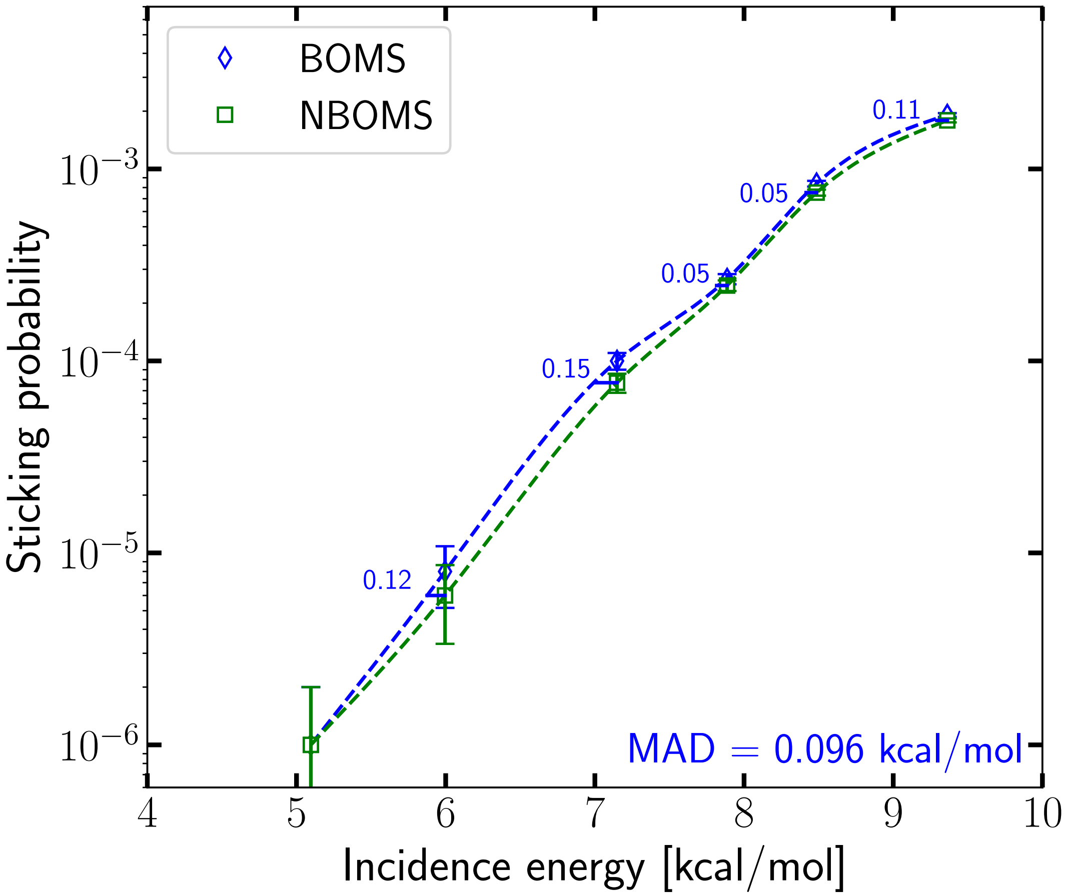

Figure S16. Sticking probabilities computed for H_2 + Al(110) using the HDNN PES with the BOMS and the NBOMS model are compared with one another, showing the effect of allowing electron hole pair excitation on the sticking probability at T_s = 220 K.

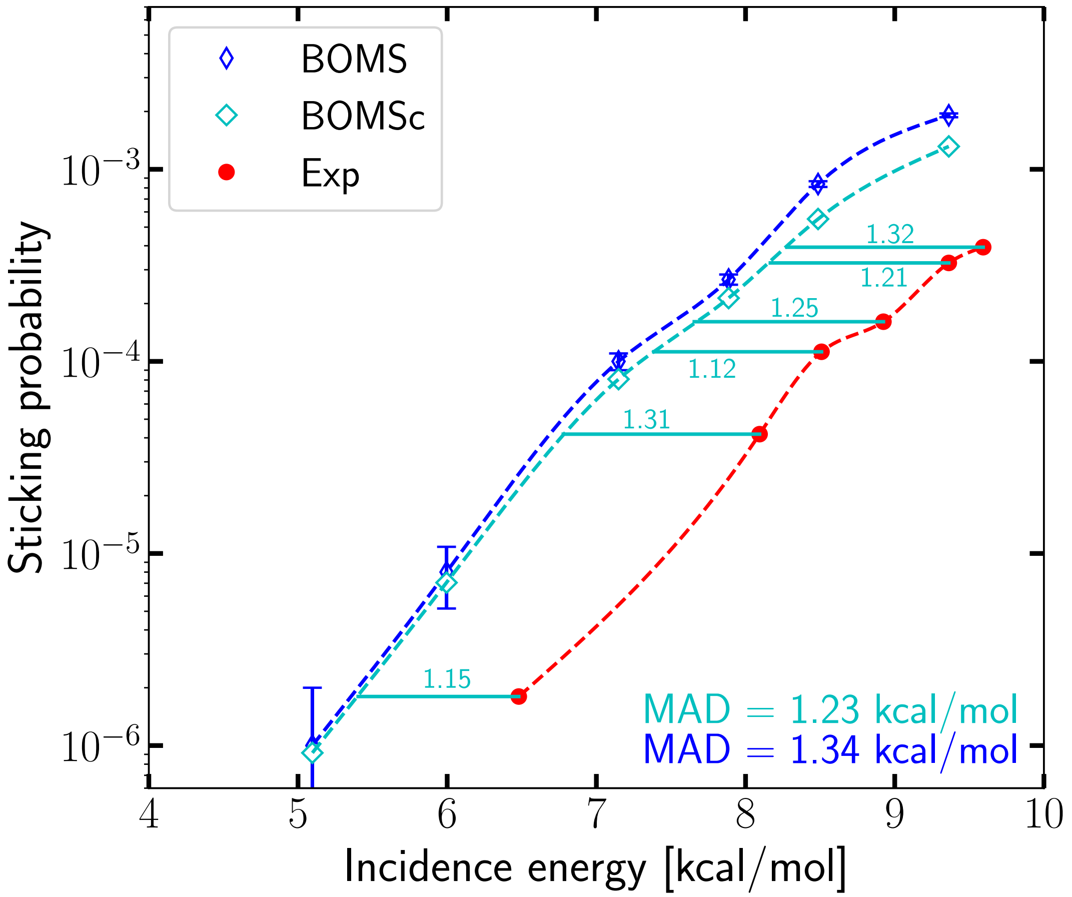

Figure S17. Sticking probability curve computed for H_2 + Al(110) using the HDNN PES with the BOMS model for T_s = 220 K (BOMS), and the same sticking probability curve corrected for the too low barrier of BG2 in the QMC-DFT energies and the HDNNP (BOMSc). The corrected sticking probabilities are given by $S_0^{cor}(E_i) = S_0(E_i) + S_0^{BG2}(E_i - 1.6 \text{ kcal/mol}) - S_0^{BG2}(E_i)$.

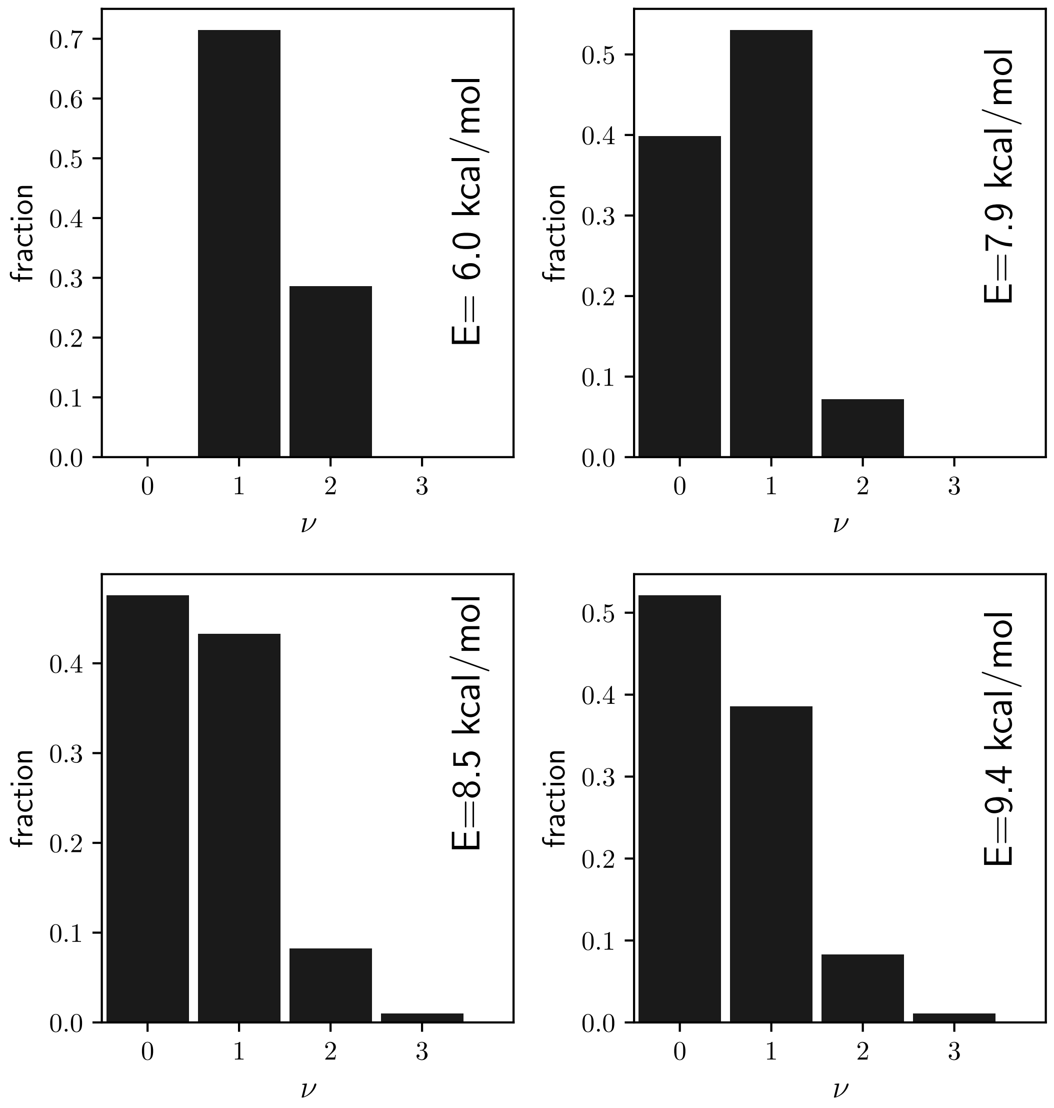

Figure S18. Of the molecules that react, shown are the fractions initially incident in the vibrational states *v*=0, 1, 2, and 3, at incidence energies of 6.0, 7.9, 8.5, and 9.4 kcal/mol.

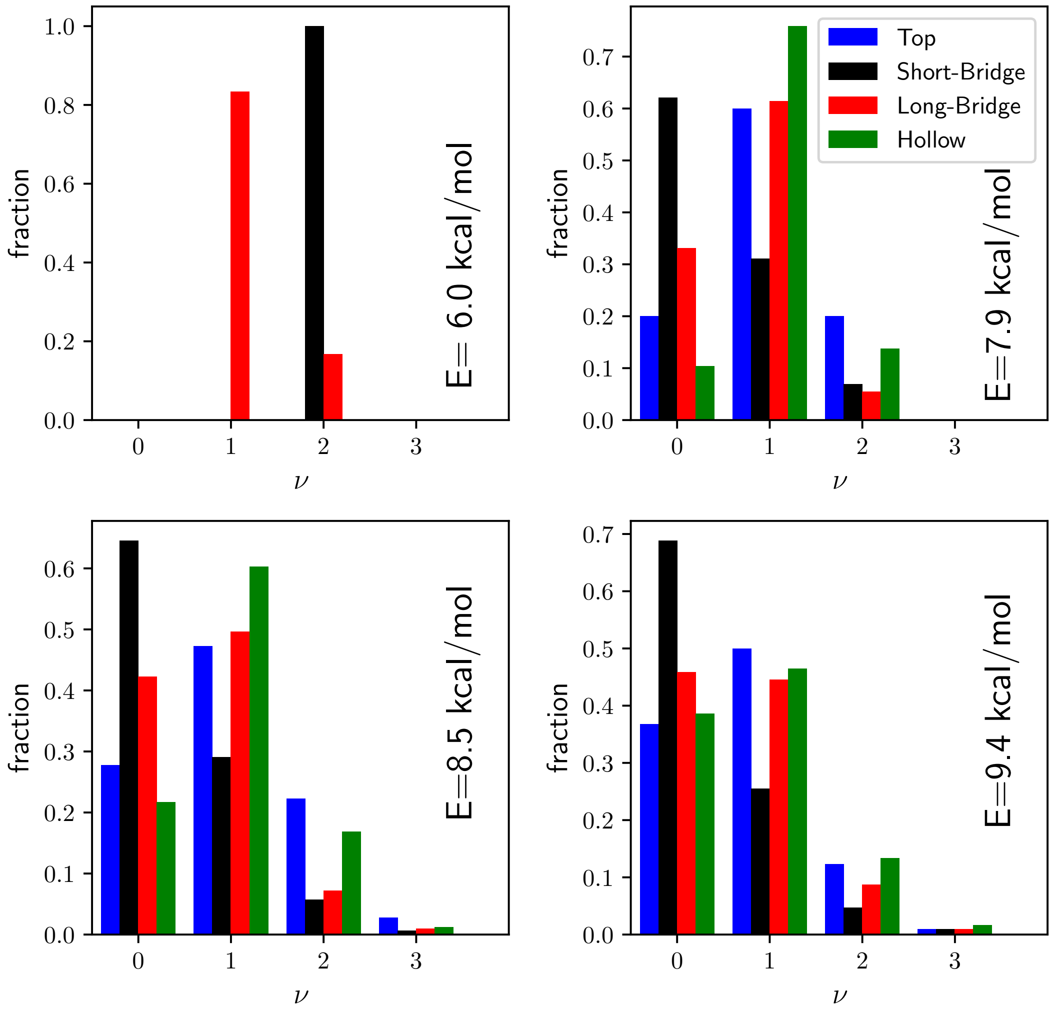

Fig.S19. Of the molecules that react at a specific impact site and incidence energy, at average incidence energies of 6.0, 7.9, 8.5, and 9.4 kcal/mol, shown are the fractions initially incident in the vibrational states v=0, 1, 2, and 3 on that site.

Supporting tables.

BG	Geometry	E_b^{DFT}, 220 K	E_b^{DFT}, 0 K
BG1	Long bridge, ϕ=0°	25.3 (-0.1)	25.4
BG2	Short bridge, ϕ=90°	24.8 (-0.3)	25.1
BG3	Hollow, ϕ=0°	37.5 (-0.1)	37.6
BG4	Top, ϕ=90°	37.8 (0.0)	37.8
BG5	Long bridge, ϕ=45°	34.8 (-0.1)	34.9
BG6	Short bridge, ϕ=0°	49.4 (-0.2)	49.6

Table S1. The energies computed with the QMC-DF at the DMC barrier geometries (E_b^{DFT}, in kcal mol) computed for the 220 K slab are compared with the QMC-DFT barrier heights computed for the slab that was relaxed with respect to vacuum ("0 K"). The difference between the 220 K and the 0 K energies is stated in brackets in the column showing QMC-DFT energies for 220 K.

Reference atom type	Neighboring atom type	η (bohr^{-2})
H	H	0
H	H	0.005
H	H	0.014
H	H	0.037
H	H	0.113
H	H	0.768
H	Al	0
H	Al	0.002
H	Al	0.005
H	Al	0.010
H	Al	0.017
H	Al	0.030
H	Al	0.052
H	Al	0.098
H	Al	0.221
H	Al	0.768

Table S2. The η-values for the H-H pair symmetry functions and the H-Al pair symmetry functions are provided (see also Eq. S4).

Reference atom type	Neighboring atom type	η (bohr^{-2})
Al	H	0
Al	H	0.002
Al	H	0.005
Al	H	0.010
Al	H	0.017
Al	H	0.030
Al	H	0.052
Al	H	0.098
Al	H	0.221
Al	H	0.768
Al	Al	0
Al	Al	0.003
Al	Al	0.009
Al	Al	0.019
Al	Al	0.041
Al	Al	0.098
Al	Al	0.334

Table S3. The η-values for the Al-H pair symmetry functions and the Al-Al pair symmetry functions are provided (see also Eq. S4).

Reference atom type	Neighboring atom type 1	Neighboring atom type 2	λ	ζ
H	H	Al	1	1
H	H	Al	1	1.5
H	H	Al	1	2.5
H	H	Al	1	5
H	H	Al	1	13.5
H	H	Al	1	70
H	H	Al	-1	1
H	H	Al	-1	1.7
H	H	Al	-1	3.2
H	H	Al	-1	6.2
H	H	Al	-1	16
H	Al	Al	1	1
H	Al	Al	1	1.5
H	Al	Al	1	2.5
H	Al	Al	1	5
H	Al	Al	1	13.5
H	Al	Al	1	70
H	Al	Al	-1	1
H	Al	Al	-1	1.6
H	Al	Al	-1	2.7
H	Al	Al	-1	5
H	Al	Al	-1	12

Table S4. The λ and ζ parameters for use in Eq.S5 are provided for the three-body symmetry functions, for the H-atom ANN.

Reference atom type	Neighboring atom type 1	Neighboring atom type 2	λ	ζ
Al	H	H	1	1
Al	H	H	1	1.5
Al	H	H	1	2.5
Al	H	H	1	5
Al	H	H	1	13.5
Al	H	H	1	70
Al	H	H	-1	1
Al	H	H	-1	1.7
Al	H	H	-1	3.2
Al	H	H	-1	6.2
Al	H	H	-1	16
Al	H	Al	1	1
Al	H	Al	1	1.5
Al	H	Al	1	2.5
Al	H	Al	1	5
Al	H	Al	1	13.5
Al	H	Al	1	70
Al	H	Al	-1	1
Al	H	Al	-1	1.6
Al	H	Al	-1	2.7
Al	H	Al	-1	5
Al	H	Al	-1	12
Al	Al	Al	1	1
Al	Al	Al	1	1.5
Al	Al	Al	1	2.5
Al	Al	Al	1	5
Al	Al	Al	1	13.5
Al	Al	Al	1	70
Al	Al	Al	-1	1
Al	Al	Al	-1	1.5
Al	Al	Al	-1	2.5
Al	Al	Al	-1	5
Al	Al	Al	-1	13.5
Al	Al	Al	-1	70

Table S5. The λ and ζ parameters for use in Eq.S5 are provided for the three-body symmetry functions, for the Al-atom ANN.

Stage	Type of surface	Part of the PES	Range Z (Å)	Range r (Å)	Nr. points
1	Static	Gas	2.5 - 8.5	0.4 - 1.4	≈ 1000
		Solid	0.0 - 2.5	0.4 - 2.5	≈ 4000
2	Moving	Gas	2.5 - 8.5	0.4 - 1.4	≈ 3000
		Solid	0.0 - 2.5	0.4 - 2.5	≈ 12,000
3	Moving	Gas + Solid	0.0 - 8.5	0.4 - 2.5	≈ 16,000

Table S6. Information on the training of the HDNNP in different stages. See the text for additional calculations.

Geometry	E_b^{CRP}	E_b^{HDNN}	E_b^{DFT}	E_b^{DMC}	Δ_{DFT}^{HDNN}	Δ_{DMC}^{HDNN}	Δ_{DMC}^{DFT}	Δ_{CRP}^{HDNN}	Δ_{DFT}^{CRP}
BG1	25.8	25.0	25.3	25.0	-0.3	0.0	0.3	-0.8	0.5
BG2	24.8	24.5	24.8	26.4	-0.3	-1.9	-1.6	-0.3	0.0
BG3	37.7	36.9	37.5	35.0	-0.6	1.9	2.5	-0.8	0.2
BG4	38.0	37.3	37.8	36.6	-0.5	0.7	1.2	-0.7	0.2
BG5	35.6	35.6	34.9	33.6	0.8	2.0	1.3	0.0	0.7
BG6	49.5	47.8	49.4	46.8	-1.6	1.0	2.6	-1.6	0.1
MSE	-	-	-	-	-0.4	0.6	1.1	-0.6	0.3
MAE	-	-	-	-	0.7	1.3	1.6	0.6	0.3

Table S7. Single point energies E_b computed for the DMC barrier geometries with DMC (E_b^{DMC}), QMC-DFT (E_b^{DFT}), and the HDNNP (E_b^{HDNN}) and the CRP (E_b^{CRP}) fits to the QMC-DFT data are compared for a surface temperature of 220 K. The DMC barrier heights have been adjusted for thermal effects as described in the text. Below, the notation Δ_A^B refers to the difference between the barrier heights computed with methods B and A, i.e., $\Delta_A^B = E_b^B - E_b^A$. All results are in kcal/mol.

Geometry	E_{sp}^{CRP}	E_{sp}^{HDNN}	E_{sp}^{DFT}	E_{b}^{DMC}	Δ_{DFT}^{HDNN}	Δ_{DMC}^{HDNN}	Δ_{DMC}^{DFT}	Δ_{CRP}^{HDNN}	Δ_{DFT}^{CRP}
BG1	25.4	25.0	25.4	25.0	-0.4	0.0	0.4	-0.4	0.0
BG2	24.8	24.5	24.7	26.4	-0.2	-1.9	-1.7	-0.3	0.1
BG3	37.7	37.0	37.4	35.0	-0.4	2.0	2.4	-0.7	0.3
BG4	38.0	37.5	37.8	36.6	-0.3	0.9	1.2	-0.5	0.2
BG5	35.6	35.7	34.9	33.6	0.8	2.1	1.3	0.1	0.7
BG6	49.4	47.8	49.3	46.8	-1.5	1.0	2.5	-1.6	0.1
MSE	-	-	-	-	-0.3	0.7	1.0	-0.6	0.2
MAE	-	-	-	-	0.6	1.3	1.6	0.6	0.2

Table S8. Saddle point energies E_b computed for the two-dimensional saddle point geometries with QMC-DFT (E_{sp}^{DFT}), and the HDNNP (E_{sp}^{HDNN}) and the CRP (E_{sp}^{CRP}) fits to the QMC-DFT data are compared to one another and to the DMC barrier heights (E_{b}^{DMC}) for a surface temperature of 220 K. The DMC barrier heights have been adjusted for thermal effects as described in the text. Below, the notation Δ_{A}^{B} refers to the difference between the energies computed with methods B and A, i.e., $\Delta_{A}^{B} = E_{b}^{B} - E_{b}^{A}$. All results are in kcal/mol.
