# Supplementary material for: Best-of-Both-Worlds Predictive Approach to Dissociative Chemisorption on Metals
Source: J Phys Chem Lett. 2024 Jan 3;15(1):307–15. doi: 10.1021/acs.jpclett.3c02972 (PMC10788952; doi:10.1021/acs.jpclett.3c02972)
Supplement: Supplementary file 2 — jz3c02972_si_002.pdf [file jz3c02972_si_002.pdf]

jz-2023-02972d.R1

Name: Peer Review Information for "Best-of-both-worlds Predictive Approach to Dissociative Chemisorption on Metals"

First Round of Reviewer Comments

Reviewer: 1

Comments to the Author

The authors in this manuscript propose an improved approach over standard DFT in constructing the ground state PES for describing dissociative chemisorption of molecules on metal surfaces. This so-called QMC-DFT approach relies on adjusting the exchange part of the GGA-based density functional to match the reaction barrier height predicted by the quantum Monte-Carlo method, which is then used in subsequent single point calculations for the PES construction. Molecular dynamics calculations show that such a QMC-DFT based potential for H2 dissociative chemisorption on Al(110) is able to reduce errors between calculated and experimentally measured sticking probabilities to ~1.4 kcal/mol. While this is a comprehensive study with respect to the tests on the performance of QMC-DFT in this specific system, I do not think that it demonstrates sufficient novelty and provides new physical insights to the system and the field. I cannot recommend this manuscript in its current form for publication to J. Phys. Chem. Lett. and would reconsider it as an article in J. Phys. Chem. C.

The most advance reported in the paper is using QMC as a benchmark to adjust GGA-DFT. But this idea is not new, as some of the authors have tried to adjust GGA-DFT in an empirical way to match experimental sticking probabilities in previous studies. The only difference is using the single point energy of QMC at the minimum transition state to do the adjustment. Indeed, this treatment is not well justified because the differences between QMC and DFT at different configurations are unlikely identical. This can be clearly seen in Table 1, after the correction, the QMC-DFT barrier heights differ from QMC values by at most 2.6 kcal/mol, which is twice larger than the translational energy difference between the calculated and experimental sticking coefficient curves. This simplified correction does not consider the realistic potential energy landscape at the QMC level, e.g. the width of the barrier, the site-specific reactivity, the anisotropy in the entrance channel, etc. A more reasonable way is probably using the DFT PES as a basis and add a limited number of data points at the QMC level that refines the PES to a higher-level. Overall, I think the aforementioned advance is just incremental.

Furthermore, I do not see an immediate significance of this advance. I find no comparison of the calculated sticking probability curves obtained by the DFT PES and the QMC-DFT PES, which should be

included in the paper, but I guess they only differ by some incidence energy shift. In other words, the new PES does not seem to change the physical behavior of the chemisorption of H_2 on Al(110) and provide new physical insights to the system.

Technical questions.

The authors made some tests on how surface motion and electronic friction affects the sticking probabilities, but their influences are already known to be very minor for H_2 dissociative chemisorption on metal surfaces in literature. In this regard, I think these numbers (MAE) in Figure 2 carry little valuable information. To be honest, there are several sources of error that may affect the MAE. The convergence of DFT energies with respect to cell size, number of layers and k-points can hardly reach 0.2 kcal/mol (or 20 meV) or smaller. The convergence of quantum Monte-Carlo calculations is not a trivial task either and may at least have the same magnitude of error. In addition, the neural network PES itself has a test error on the order of ~30 meV. Error accumulations and error cancellations may coexist, so why bother the slight MAE change (and effective digits lower to 0.01 kcal/mol) in Figure 2?

The authors mention in page 4 that Recent hybrid DFT calculations effectively using long range screening achieved good agreement with semi-empirical reference barriers, but these calculations erroneously used zero-point energy corrections and surface atom relaxation in the presence of the molecule.

Could the authors explain more about errors of using zero-point energy corrections and surface atom relaxation in the presence of the molecule?

Reviewer: 2

Comments to the Author

The paper addresses shortcomings of DFT methods for calculations of reaction barriers by combining it with QMC approach. In particular, the idea to construct DFT functional using QMC data with subsequent much more accurate DFT study was here to be explored - the authors did solid job in this direction. The results look solid and the paper is clearly written with details in Suppl. material.

I recommend publishing in JPCL.

Author's Response to Peer Review Comments:

Reviewer: 1 (Comments were not numbered; below we split up the paragraphs, where the letter indicates the paragraph in the report).

Reviewer comments in black, author responses in blue, new text in manuscript in red, text present already in previous manuscript important to rebuttal in green.

Recommendation: Reconsider as an article in The Journal of Physical Chemistry A/B/C.

We will argue below that our paper reports a **significant scientific advance** such that rapid publication is essential. For this reason it falls within the scope of JPCL, and it should be published in JPCL.

Comments:
The authors in this manuscript propose an improved approach over standard DFT in constructing the ground state PES for describing dissociative chemisorption of molecules on metal surfaces. This so-called QMC-DFT approach relies on adjusting the exchange part of the GGA-based density functional to match the reaction barrier height predicted by the quantum Monte-Carlo method, which is then used in subsequent single point calculations for the PES construction. Molecular dynamics calculations show that such a QMC-DFT based potential for H2 dissociative chemisorption on Al(110) is able to reduce errors between calculated and experimentally measured sticking probabilities to ~1.4 kcal/mol. While this is a comprehensive study with respect to the tests on the performance of QMC-DFT in this specific system, I do not think that it demonstrates sufficient novelty and provides new physical insights to the system and the field. I cannot recommend this manuscript in its current form for publication to J. Phys. Chem. Lett. and would reconsider it as an article in J. Phys. Chem. C.

We reiterate, and will argue below, that our paper reports a significant scientific advance, i.e., a novel and important computational approach, such that it deserves rapid publication in JPCL. If published in JPCC the paper would not receive sufficient attention, and the urgency of the paper might not be discerned by readers not paying close attention to our work.

A1. The most advance reported in the paper is using QMC as a benchmark to adjust GGA-DFT. But this idea is not new, as some of the authors have tried to adjust GGA-

DFT in an empirical way to match experimental sticking probabilities in previous studies.

The logic in the statement of the reviewer, which consists of two sentences, is flawed. To demonstrate this we will first paraphrase the reviewer's statement in a simplified manner: "The authors use method A to do C. Because they have done C using method B, their idea is not new."

Our point here is twofold: what we do is new because we use a different method (A instead of B) to "do C", and this is important because method A is much more powerful, method A being predictive because it is based on first principles, and method B being semi-empirical.

To make this argument more concrete: Adjusting a parameter in a parameterized functional to reproduce the **DMC energy for the minimum barrier geometry** is new. The fact that some of us previously adjusted a parameter in a functional to reproduce **a measured sticking probability** does not change that. What we do is **new** because it is **based on first principles, making the method predictive**. The previous work was **semi-empirical** in that it had to rely on **a pre-existing experiment for the same system to extract a barrier height for that system**.

A point of lesser importance: the first sentence of the reviewer is also not really correct concerning what we do ("C" above). More precisely, we use QMC (actually, Diffusion Monte-Carlo, acronym DMC) to adjust the exchange-part of a non-local exchange correlation functional, which consists of a GGA exchange part, and a non-local Chalmers-Rutgers van der Waals correlation part (**non-GGA**).

The novelty of now using a predictive first principles approach and the importance of using such an approach had already been emphasized in text in the originally submitted manuscript, now made green in the resubmitted draft to be used for editorial and review purposes to emphasize that the rebuttal to this point is actually already present in the originally submitted draft, in text appearing on pages 4 and 5:

In the present state-of-the-art chemically accurate dissociative chemisorption barriers are available for few (i.e., fourteen [5]) systems, which are characterized by limited charge transfer from the metal to the molecule [20]. These barriers had to be obtained using a semi-empirical DFT approach [12, 21] that requires well-documented experimental data [5, 12]. Tests employing a database with reference barriers for this

limited class of systems show that the standard DFs used in surface science, i.e., DFs using semi-local exchange, yield errors in $E_b \geq 2.4$ kcal/mol [5].

To go beyond the current state-of-the-art, we need a fully predictive – as opposed to semi-empirical – electronic structure approach that also works for systems with considerable charge transfer [20]. In such systems, in which the molecule usually has a high affinity for electrons (making these systems potentially relevant to sustainable chemistry, e.g. oxygen containing molecules), electronically non-adiabatic effects like electron-hole pair (ehp) excitation are likely to strongly affect the reaction dynamics [22]. The accuracy of theories for dealing with these non-adiabatic effects in dynamics calculations on reactive scattering has not yet been established [22-24]. Tuning a semi-empirical DF in an attempt to compensate for errors introduced by an inaccurate non-adiabatic approach would likely result in serious errors in the reaction barrier.

A predictive approach is also needed for systems for which experiments are not available, are not well-documented, or yield conflicting results [12]. Finally, a much more accurate approach than now available is needed if the field of computational surface reaction dynamics is ever to match the level of detail in the characterization of reaction mechanisms now available for gas phase reactions [25].

To emphasize the difference between the old semi-empirical and the new predictive first principles approach, a sentence has now been rewritten on page 5 as:

In its simplest version we construct a tunable DF (a quantum-Monte Carlo based density functional (QMC-DF)). Instead of adjusting a parameter in this DF semi-empirically as done earlier [12, 21] we now adjust it so that the DF reproduces the DMC energy at a point near the transition state. The new method is therefore predictive, and based on first principles.

A2. The only difference is using the single point energy of QMC at the minimum transition state to do the adjustment.

Yes, but this difference is **crucial**, as fitting the density functional to a Diffusion Monte-Carlo result makes our new approach **predictive**! We reiterate that the previous approach to which the reviewer refers was **semi-empirical**. This means that barrier heights could only be extracted for **systems for which well-documented experiments** are already available, and for which the charge transfer energy (the work function of the surface minus the electron affinity of the molecule) is greater than 7 eV. This means that the previous semi-empirical approach to extracting barrier heights cannot be applied to **most systems that are of interest to sustainable**

chemistry (which usually contain oxygen). We are not stating new facts: most of this information was already present in the introductory paragraphs of the previous version of the paper. The reason that our paper reports a **significant scientific advance justifying rapid communication in JPCL** is that our new QMC-DFT approach is not only **accurate** (to within about 1.5 kcal/mol) and **affordable** (because DFT can be used to map out the PES), but also **predictive** (in the sense that no new or existing experiment is required to extract the barrier height for a new system, the experiment is used here **only for validation purposes**).

All of the above was essentially already in the paper, i.e., in the green text appearing on pages 4 and 5 and reproduced in answer to comment A1. The relevance of our novel method to sustainable chemistry is now also made clear, in the new sentence appearing on page 4:

In such systems, in which the molecule usually has a high affinity for electrons (making these systems potentially relevant to sustainable chemistry, e.g. oxygen containing molecules), electronically non-adiabatic effects like electron-hole pair (ehp) excitation are likely to strongly affect the reaction dynamics [22].

We also note that the emphasis on the predictive character of our new approach was already present in the abstract of the previously submitted manuscript, in the following two sentences:

Here we present an approach in which we use diffusion Monte Carlo (DMC) to pin the minimum barrier height and construct a density functional that reproduces this value. This predictive approach allows constructing a potential energy surface at the cost of density functional theory while retaining near DMC accuracy.

A3. Indeed, this treatment is not well justified because the differences between QMC and DFT at different configurations are unlikely identical. This can be clearly seen in Table 1, after the correction, the QMC-DFT barrier heights differ from QMC values by at most 2.6 kcal/mol, which is twice larger than the translational energy difference between the calculated and experimental sticking coefficient curves.

For clarity: The reviewer means to say that the fitted barrier heights computed with QMC-DFT differ from the DMC **by up to** 2.6 kcal/mol. **Overall** these errors are much smaller, as should be clear from the MSE (mean signed error) and MUE (mean unsigned error) of only 1.0 and 1.6 kcal/mol, respectively.

What is relevant here is the extent to which the differences between QMC and **QMC-DFT** barrier heights are **small**. The differences do not need to be zero, but they need to be small enough. Most important in this context is that they are small enough to allow **validation** of the DMC transition state energy, and possibly of other DMC barrier heights (mostly in reduced dimensionality) by **comparison to the experiments available now**.

Our comparison to the available experiment shows that the large deviation noted by the reviewer for one specific geometry, which corresponds to the highest barrier (BG6), is not relevant to the validation now: As can be seen from Figs.2A and 2B the computed sticking probability curves are displaced from the measured one by a shift that is **reasonably constant**, varying by only small amounts. This would not be the case if the energy difference noted by the reviewer for BG6 would matter to the comparison with the experiment. Furthermore, the barrier geometry (BG6) for which the difference of 2.6 kcal/mol occurs is not relevant because the corresponding DMC barrier height exceeds the DMC minimum barrier height by more than 20 kcal/mol! The point made is now emphasized in new text appearing on pages 10 and 11 as:

A few points are worth emphasizing regarding Figs.2A and 2B. The first point is relevant to the accuracy with which the QMC-DF reproduces the DMC energies of BG3-BG6. The errors in BG3 and BG6 may appear as rather large (2.5 and 2.6 kcal/mol, respectively). Fig.2B suggests that the computed quantum corrected sticking probability is not sensitive to such discrepancies over the range of E_i for which experimental results are available for validation: the computed sticking curve appears shifted relative to the interpolated experimental curve by a reasonably constant energy shift, ranging between 1.25 and 1.63 kcal/mol. In this particular case this may well be because the DMC energies of BG3 and BG6 are higher than that of BG1 by $\geq$ 10 kcal/mol.

A4. This simplified correction does not consider the realistic potential energy landscape at the QMC level, e.g. the width of the barrier, the site-specific reactivity, the anisotropy in the entrance channel, etc.

Previous semi-empirical calculations show that our straightforward approach, which relies on DFT accurately describing the variation of barrier height with geometry, is already sufficient. This is now explained in the new manuscript by the following text fragment appearing on page 5:

We also use the finding that DFT is quite accurate for the *variation* of E_b with system geometry. This is demonstrated by the success achieved with the previously mentioned semi-empirical DFT method [5, 12]. Specifically, it was possible to reproduce measured sticking probability curves over large ranges of incidence energies E_i by adjusting only one parameter in the semi-empirical DF, with this parameter mainly affecting the minimum barrier height, and therefore the threshold of the sticking curve. The fact that also the shape (width, or conversely, the slope) of the curve was well reproduced for already fourteen systems [5, 12] must [26] mean that, in general, DFT is accurate for the variation of the barrier height with geometry, and of the PES in the vicinity of the transition state, once the minimum barrier height is pinned.

What we are saying here is: we would never have been able to obtain good overall agreement with measured sticking probability curves (which can be characterized by a threshold parameter and a parameter describing the width, or conversely, the slope of the curve) if DFT would not be good for the variation of the barrier height with geometry, and of the PES in the vicinity of the transition state. We say this because all we ever did was to adjust one parameter in a density functional, which mainly affects the minimum barrier height for the system.

Also, on page 6 a point previously already made in the first submitted manuscript is now emphasized even more:

A point worth noting from Table 1 is that comparison to DMC shows that the QMC-DF is rather good at describing the *variation* of barrier height with geometry for H_2 + Al(110), the deviations from the DMC values being much smaller than the energy range spanned by the DMC energies of BG1-BG6. This observation was already made for the eight standard DFs compared to DMC for H_2 + Al(110) earlier [27], and gives support to our earlier remark that the success of semi-empirical DFT for DC on metals is due to DFT being good at describing the variation of the barrier height with system geometry.

On page 12 of the previous manuscript we already discussed in the first submitted manuscript a multitude of measures that can be taken to get a QMC-DFT approach in which the DMC barrier heights are even better reproduced than they already were, which was sufficient for the system we here address, on page 14 (but see also our response to point A5 below for the new red text):

Ascending the rungs on Jacob's ladder of DFs might also further improve the DF's ability to reproduce the variation of E_b with system geometry beyond that already achieved using DFs with semi-local exchange here and elsewhere [12, 27]. If the DF remains inaccurate for this variation one can partition the PES into the molecule-surface interaction and the potential describing the solid [48]. Then, in the calculation of the molecule-surface interaction one can make the parameter in the QMC-DF dependent on X, Y, and ϕ using symmetry adapted functions [49-50]. This has been done earlier for potential expansion functions [50]. In the future it will probably be possible to derive a true DMC-quality PES by adding a high-dimensional neural network (HDNN) PES based on the difference between say a thousand DMC energies and a QMC-DFT PES as obtained here, in the spirit of the Δ-machine learning approach recently used to obtain a CCSD(T) level PES for MD simulations of liquid water [51]. We expect that with such systematic improvements the QMC-DFT approach can ultimately attain chemical accuracy (errors ≤ 1 kcal/mol) in the description of experiments of dissociative chemisorption on metals.

A5. A more reasonable way is probably using the DFT PES as a basis and add a limited number of data points at the QMC level that refines the PES to a higher-level. Overall, I think the aforementioned advance is just incremental.

The aforementioned approach is certainly not incremental! With our approach we achieve a much more accurate (and predictive!) calculation of the sticking probability (accurate to within an energy shift of about 1.4 kcal/mol) than achievable with the general purpose PBE DF used to prime the QMC calculations! See also our answer to comment B1 below. To emphasize this point we have added new text on page 11 stating:

One would then expect the sticking curve computed on the basis of a PBE PES to be shifted relative to experiment by about -7.5 kcal/mol. This is yet another illustration that standard GGA DFs cannot be expected to allow accurate predictions for sticking curves for DC on metal surfaces [12]. In contrast, our results suggest that parameterizing a DF on the basis of the DMC transition state energy, as done here, allows predictions for DC on metal surfaces of near-chemical accuracy.

At the same time the point of the reviewer, that it might be optimal to add a limited number of DMC points to make the PES even more accurate than it is already, is well taken. The reviewer would seem to suggest to start with a GGA DFT PES (say a PBE PES) and then to add a correction to the PES based on the DMC results. With such

tactics we would take a different approach and start with just a few DMC calculations to generate a QMC-DFT PES as the starting point for further DMC corrections. We think that in the not-to-distant-future it may well be possible to work with a true DMC quality PES in this way. However, to ensure a true DMC quality PES, one may well need a thousand or more extra DMC points, which was not possible to compute with the present resources. This may be possible in future with a Δ-machine learning approach, in the spirit in which it was done quite recently to obtain a CCSD(T) quality PES for liquid water. This is now stated on page 14 as follows:

In the future it will probably be possible to derive a true DMC-quality PES by adding a high-dimensional neural network (HDNN) PES based on the difference between say a thousand DMC energies and a QMC-DFT PES as obtained here, in the spirit of the Δ-machine learning approach recently used to obtain a CCSD(T) level PES for MD simulations of liquid water [51].

We note that while impressive agreement was reached with this approach for liquid water (errors smaller than 0.1 kcal/mol in the average binding energy of a pair of water molecules) we here quote the level of agreement achieved with experiment (about 1.5 kcal/mol) for a H_2 molecule interacting **with an entire Al slab** modeling the Al(100) surface. We also note that the cpu time needed for such a DMC type PES would be larger than now spent to compute 6 barrier geometries by at least a factor 160 if (and that is a big if) 1000 extra DMC points would be enough to achieve a true DMC quality PES. It will be a few years still before such a calculation can be done. (Computing 6 barrier energies with DMC required about 3 million cpu hours on the Dutch national supercomputer in 2018-2019; calculating 1000 points would have required about 500 million cpu hours. Marx and co-workers (of Ref.51) added 3100 CCSD(T) energies to 13,600 MP2 energies and forces to arrive at a PES for liquid water that they argue has CCSD(T) quality, according to an email exchange with Marx; this is how we arrive at our estimate that at least 1000 extra DMC points would be required to ensure a true DMC quality PES). Finally we note that the improvement in accuracy that might be achievable by going from the QMC-DFT PES to a true DMC quality PES in this way might actually be rather modest.

B1. Furthermore, I do not see an immediate significance of this advance. I find no comparison of the calculated sticking probability curves obtained by the DFT PES and the QMC-DFT PES, which should be included in the paper, but I guess they only differ by some incidence energy shift.

We did not completely understand this statement of the reviewer, because the only DFT PES making an appearance in our paper is the QMC-DFT PES. We think the reviewer wants to raise the following question: **Can the authors compare the sticking curve obtained with the QMC-DFT PES to the curve that would be obtained with a PES computed with the functional used to obtain the Kohn-Sham wave function input to the VMC and DMC calculations used to obtain DMC barrier heights?** That would then be a PBE PES, as we used the PBE DF to generate the initial wave function for the VMC calculations. The reviewer would therefore seem to ask for additional dynamics calculations using a PBE PES, and for showing a PBE sticking probability curve also in Figure 2, to illustrate the improvement achieved. We have done something simpler instead.

The question of the reviewer can also be addressed as follows. First, we have now added to Table 1 the PBE energies of barrier geometries BG1-BG6, on page 8:

Table 1. Geometries BG1-BG6 and energies associated with the DMC barriers and mean signed error (MSE) and mean absolute errors (MAE) in the QMC-DFT and PBE energies computed for the DMC barrier geometries. DMC barrier geometries, the DMC barrier heights E_b^{DMC}, the QMC-DFT energies E_b^{QMC-DF}, and the PBE energies E_b^{PBE} are provided. H_2 is always parallel to the surface (θ=90°). Differences between the QMC-DFT (PBE) and the DMC energies are provided in brackets in the column showing E_b^{QMC-DF} (E_b^{PBE}). All energies are in kcal/mol.

BG	Site	ϕ	r(Å)	Z(Å)	E_b^{DMC}	E_b^{QMC-DF}	E_b^{PBE}
BG1	Long bridge	0°	1.334	1.118	25.1±0.2	25.4 (0.3)	19.5 (-5.6)
BG2	Short bridge	90°	1.080	1.568	26.7±0.2	25.1 (-1.6)	19.7 (-7.0)
BG3	Hollow	0°	1.245	0.615	35.1±0.2	37.6 (2.5)	26.6 (-8.5)
BG4	Top	90°	1.368	1.564	36.6±0.2	37.8 (1.2)	29.5 (-7.1)
BG5	Long bridge	45°	1.361	0.786	33.7±0.2	34.9 (1.1)	26.2 (-7.5)
BG6	Short bridge	0°	1.154	1.175	47.0±0.2	49.6 (2.6)	38.1 (-8.9)
MSE						1.0	-7.4
MAE						1.6	7.4

As can be seen the PBE functional underestimates the DMC energy of the DMC transition state (for barrier geometry 1) by about 6 kcal/mol, and relative to the DMC energies the PBE DF systematically underestimates the DMC barrier energies by 7.4 kcal/mol (note that the absolute value of the negative MSE equals the MAE).

For a given dynamical model and method the computed PBE sticking probability curve would then be shifted to lower energies relative to the computed QMC-DFT sticking curve by an estimated 6 kcal/mol (which is of course more than just "some energy shift", which was the expectation voiced above by the reviewer), and by about 7.5 kcal/mol relative to the experimental curve. In other words: standard DFT with the general purpose PBE density functional can not be trusted to make a reliable (i.e., accurate) prediction for an activated H_2 + metal surface system, as suggested by the results for H_2 + Al(110). We have added the extra column with the PBE barriers to Table 1 on page 8 to clarify this point, which was perhaps not sufficiently stressed in our first version of the paper; if we had made this point before the reviewer probably would not have made the statement "I guess they (the two different sticking curves computed with PBE-DFT and QMC-DFT, we think, authors' note) only differ by some incidence energy shift"). To emphasize this point, we have also included new text with the discussion of Figure 2 on page 11:

The second point is that, according to Table 1, a dynamics calculation like the quantum corrected one now presented in Fig.2B but based on the PBE DF would have been of essentially no predictive value for H_2 + Al(110). The PBE DF underestimates the DMC energy of BG1 (by about 6 kcal/mol) and the QMC-DF sticking curve is shifted to lower energies relative to the experimental one by about -1.5 kcal/mol. One would then expect the sticking curve computed on the basis of a PBE PES to be shifted relative to experiment by about -7.5 kcal/mol. This is yet another illustration that standard GGA DFs cannot be expected to allow accurate predictions for sticking curves for DC on metal surfaces [12]. In contrast, our results suggest that parameterizing a DF on the basis of the DMC transition state energy, as done here, allows predictions for DC on metal surfaces of near-chemical accuracy.

In closing our response to point B1: of course we could have gone through an elaborate exercise to demonstrate by actual dynamics calculations that the use of a PBE PES would result in a curve shifted to lower energies relative to experiment by 7.5 kcal/mol. However, we have already done this type of work in 2009 (Kroes and co-workers, Science 326, 832, 2009) where we showed in Fig.1A of that paper (reproduced on the next page) that the PW91 DF yields a sticking probability curve shifted to lower energies by about 15 kJ/mol relative to the SRP-DFT result (the difference between the PW91 and the SRP-DFT barrier heights being 14 kJ/mol ≈3.3 kcal/mol), and that the RPBE DF yields a sticking probability curve shifted to lower energies by about 30 kJ/mol relative to the PW91 result (the difference between the

RPBE and the PW91 barrier heights being 32 kJ/mol ≈ 7.6 kcal/mol). It seemed to us that is not worth the effort to go through a similar exercise again.

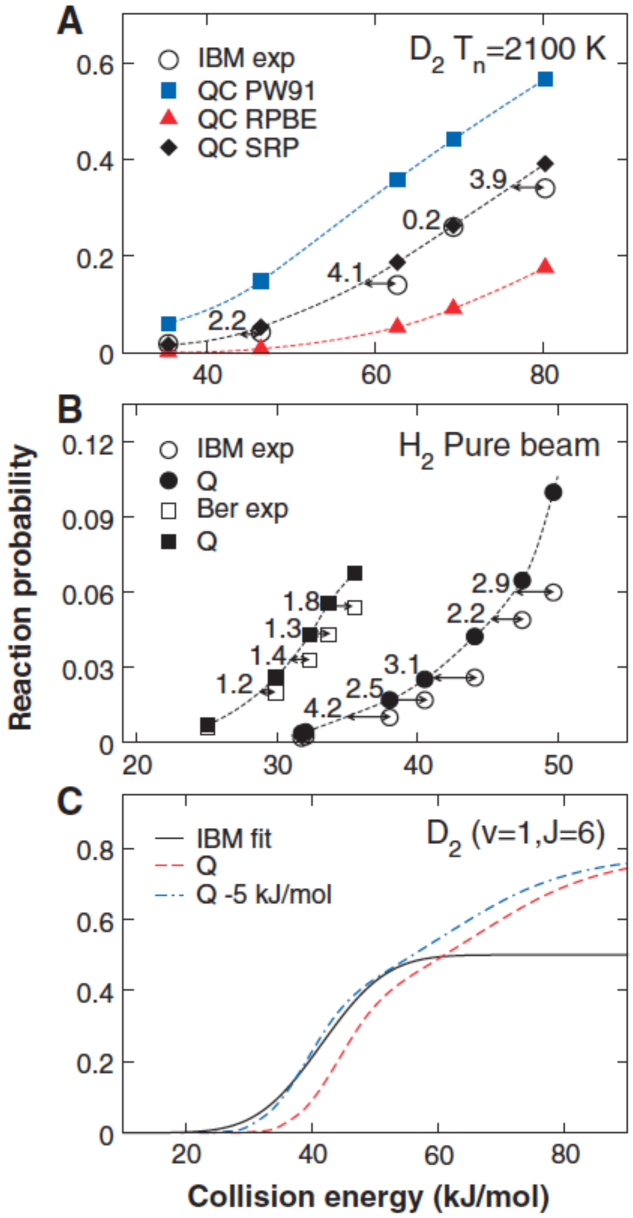

Fig. 1. Reaction probabilities computed with the quasi-classical trajectory (QC) method and with quantum dynamics (Q) using SRP-DFT are compared with experimentally measured values for D_2 (*18*) and H_2 (*17, 31*) + Cu(111). Experimental results from (*17, 18*) are labeled "IBM" and those from (*31*) "Ber". (**A**) Comparison to experiments on D_2 using a nozzle temperature of 2100 K. Reaction probabilities computed with the PW91 and the RPBE PES are also shown. The QC results exhibit statistical errors, but in all cases the SDs are ≤0.005. (**B**) Comparison to two experiments using

B2. In other words, the new PES does not seem to change the physical behavior of the chemisorption of H_2 on Al(110) and provide new physical insights to the system.

If the reviewer meant to say that the new (i.e QMC-DFT) PES does not yield a qualitatively different description of the dynamics of reactive scattering of H_2 from Al(110) than would be obtained with a PBE PES, then this statement is obviously wrong. The system described by a PBE PES is expected to become reactive at an energy that is 7.5 kcal/mol too low compared to experiment, while the difference with experiment is only 1.4 kcal/mol for the new predictive QMD-DFT approach. This new physical (or chemical, JPCL is a chemistry/physics journal) insight obtained here is phrased as follows on page 11:

One would then expect the sticking curve computed on the basis of a PBE PES to be shifted relative to experiment by about -7.5 kcal/mol. This is yet another illustration that standard GGA DFs cannot be expected to allow accurate predictions for sticking curves for DC on metal surfaces [12]. In contrast, our results suggest that parameterizing a DF on the basis of the DMC transition state energy, as done here, allows predictions for DC on metal surfaces of near-chemical accuracy.

Calculations with the PW91 and the QMC-DFs on scattering back to the gas phase would also provide very different physical observations. In previous calculations on H_2 + Cu(111) we have considered differences between probabilities for rotationally and diffractionally inelastic scattering computed on the basis of the PW91 and RPBE GGA functionals. These functionals yield values of the minimum barrier height that differ by 7.6 kcal/mol, which is not so different from the mentioned difference of 6 kcal/mol for H_2 + Al(110) between PBE and the QMC-DF. The work (C. Díaz et al., JPCC114, 11192, 2010) predicted very large differences between the mentioned scattering probabilities computed with PW91 and RPBE. For the same system calculations based on the PW91 and SRP functionals predicted radically different outcomes of experiments on vibrational excitation, even though the minimum barrier heights computed with these functionals only differ by 3.3 kcal/mol! (see Figures S2 and S3 of G.J. Kroes et al., PNAS 107, 20881, 2010). This is now stated in the text on page 11 as:

Furthermore, calculations on H_2 + Cu(111) using PESs calculated with different DFs and different minimum barrier heights [21] suggest that analogous calculations on H_2 + Al(110) with the PBE and the QMC DFs should yield qualitatively differing results

for rotationally and diffractive inelastic scattering [36] and very different predictions for experiments on vibrationally inelastic scattering [37].

Technical questions.

C1. The authors made some tests on how surface motion and electronic friction affects the sticking probabilities, but their influences are already known to be very minor for H2 dissociative chemisorption on metal surfaces in literature. In this regard, I think these numbers (MAE) in Figure 2 carry little valuable information. To be honest, there are several sources of error that may affect the MAE. The convergence of DFT energies with respect to cell size, number of layers and k-points can hardly reach 0.2 kcal/mol (or 20 meV) or smaller. The convergence of quantum Monte-Carlo calculations is not a trivial task either and may at least have the same magnitude of error. In addition, the neural network PES itself has a test error on the order of ~30 meV. Error accumulations and error cancellations may coexist, so why bother the slight MAE change (and effective digits lower to 0.01 kcal/mol) in Figure 2?

The reviewer appears to call into question the convergence of the QMC results and the DFT results. Note: 0.2 kcal/mol is about 9 meV, not 20 meV as suggested by the reviewer.

Regarding the convergence of the DMC results, we now state on page 5 of the SI:

As discussed in an exhaustive error analysis presented in Ref. [2] and the Supporting Information to Ref.[2], the statistical error in the DMC barrier heights is estimated to be 0.2 kcal/mol, while the systematic error in the DMC barrier heights is expected to fall well below 1 kcal/mol.

These numbers reflect the current state-of-the art of DMC for molecule-metal surface interactions.

Regarding the DFT calculations, on pages 7 and 8 of the new version of the SI, as in the earlier version, we state:

The input parameters to the QMC-DFT calculations of the molecule-surface interaction energies were based on convergence tests on DFT energies computed for the QMC BG1 and BG2 geometries of the molecule relative to the surface. A full

account of these tests and of the input parameters used in the QMC-DFT calculations is given in Section S2 of the SI to Ref.[8]. Based on these convergence tests, we expect the QMC-DFT calculations of the molecule-surface interaction energy to be converged to within better than 0.5 kcal/mol. The QMC-DFT barrier heights shown in Table 1 are for the 0 K set-up of the Al(110) surface.

The reviewer then mentions a test set error of about 30 meV in the NN fit of the PES, but the reviewer forgets to mention that the errors tend to be much smaller for smaller total energies, as shown by Figs. S1B and S1A. Another view of the quality of the NN PES may be obtained by how the HDNN energies of BG1-BG6 compare with the DMC energies for these barrier geometries. The answer is that they compare amazingly well, i.e., with an MSE of 0.6 kcal/mol and an MAE error of 1.3 kcal/mol (see Section S5.3 and Table S7). The conclusion of this analysis is that the convergence of the QMC-DFT calculations and the accuracy of the HDNN PES is good enough to reflect the quality of the DMC calculations.

The question of whether it is important to investigate the effects of phonons, ehp excitations, and tunneling can then be addressed as follows. The HDNN PES is based on converged DFT data, and the fit is accurate, and in any case all calculations are done with **one and the same** PES. Since we use **one and the same PES** the effects of allowing surface atom motion and ehp excitation can be addressed. The quantum tunneling correction itself was based on another fit to obtain the static surface H_2 + Al(110) PES, i.e., the corrugation reducing procedure (CRP), and on QD and QCT calculations on this PES. The CRP PES is based on DFT data with the same set up and convergence as the data used for fitting the HDNN PES. The CRP PES exhibits an MSE and an MAE of -0.6 kcal/mol and 0.6 kcal/mol with respect to the HDNN energies of the BG1-BG6 geometries, so this fit should also be accurate, and allow meaning full corrections regarding tunneling. Again this information is in the SI, in Section S8.1 and Table S7 of the Supporting Information.

The question whether one should consider the effects of surface motion, ehp excitation, and tunneling is then easily answered with reference to another H_2/metal surface system, i.e., H_2 + Cu(111). As may be seen in the below Table the H_2 + Al(110) system is characterized by a lower mass ratio of H_2 to the surface atom, a lower value of the charge transfer energy (the difference between the work function of the metal surface and the electron affinity of the molecule), and a much lower value

System	H_2 + Cu(111)	H_2 + Al(110)
Mass ratio H_2/metal surface atom	31.4	13.3
charge transfer energy (WF-EA)	8.1 eV	7.4 eV
max S_0 measured	10^{-1}	4×10^{-4}

of the maximum measured sticking probability (by more than two orders of magnitude) than the benchmark system H_2 + Cu(111), for which system the effects referred to are indeed rather small, as noted by the reviewer. The simple Baule model dictates that, due to the different mass ratio, more energy transfer should be expected to the surface atoms for H_2 + Al(110) than for H_2 + Cu(111) and for other H_2/metal surface systems for which this effect was investigated earlier. For this reason we investigate the effect of allowing surface atom motion, as was already stated on page 3 of the SI in its previous version:

Keeping the surface atoms fixed is usually a very good approximation for molecules sticking to cold transition metal surfaces [5]. However, according to the Baule model, the mass ratio of H_2 (mass 2) and Al (mass 27) is more conducive to energy transfer than the mass ratio of, for instance, H_2 and Cu (mass 64). For this reason, and because the measured sticking probabilies are as small as 2×10^{-6}, we also perform BOMS calculations on H_2 + Al(110).

Note that this text also already mentioned the low sticking probabilities observed for H_2 + Al(110).

Lower values of the charge transfer energy correlate with larger electronically non-adiabatic effects like electron-hole pair excitation, so these effects are likely more important for H_2 + Al(110) than for H_2 + Cu(111) (see again the above Table for the charge transfer energies). This was not yet mentioned in the paper or the SI. Finally, effects on the reaction probability of energy transfer to the surface atoms and to electron-hole pair excitation and of tunneling, which can be small in absolute value, may still have large effects in terms of relative values if the sticking probability is very small (smaller by more than 2 orders of magnitude) as seen for H_2 + Al(110). For the purpose of demonstrating the quality of a new electronic structure approach to computing the potential energy surface, and noting that Fig.2 compares computed and measured sticking probability curves using a log scale for probabilities, it is therefore imperative to show what the size of these effects are when comparing with experiment, as we did.

To emphasize these points now also in the paper we have added the following text to page 17:

We have investigated the effect of allowing surface atom motion because the mass ratio of H_2 to Al is more favorable to energy transfer than that of H_2 to Cu [59] in the much investigated H_2 + Cu(111) benchmark system (see e.g. Ref.[60] and also Section S1 of the SI). We have investigated the additional effect of ehp excitation with the NBOMS model because the value of the charge transfer energy (i.e., the work function of the surface minus the electron affinity of the molecule) is lower for H_2 + Al(110) (7.4 eV [20, 61]) than for H_2 + Cu(111) (8.1 eV [20]), for which the effect of ehp excitation has been investigated earlier (see e.g. Ref. [33]). The reason is that lower charge transfer energies have been found to correlate with greater electronically non-adiabatic effects [22]. Finally we have investigated the importance of tunneling because the measured maximum sticking probability for H_2 + Al(110) ($\approx 0.4 \times 10^{-4}$) [29] is lower than the maximum value measured for H_2 + Cu(111) (≈ 0.1 [62]) by more than two orders of magnitude. Relative effects of energy transfer to surface atom motion and related to ehp excitation are also likely to become more important for small S_0, which is another reason for investigating the effect of these dissipation channels on a theory-experiment comparison for H_2 + Al(110).

Finally we are happy to concede the point that the combined effects of the corrections for surface atom motion, ehp excitation and tunneling are small (albeit also due to a cancellation effect, and we maintain that this could not be foreseen and that the paper needs to make these points). For this reason we have added the following text on page 11:

The third and final point is that the effects of allowing surface atom motion and ehp excitation on the one hand and tunneling motion on the other hand are small and tend to cancel each other. As can be seen from Figs. 2A and 2B the MADs computed with the BOSS model and the BOMS model corrected for tunneling are 1.51 and 1.44 kcal/mol respectively, yielding a very similar conclusion regarding the quality of QMC-DFT for H_2 + Al(110).

C2. The authors mention in page 4 that Recent hybrid DFT calculations effectively using long range screening achieved good agreement with semi-empirical reference barriers, but these calculations erroneously used zero-point energy corrections and surface atom relaxation in the presence of the molecule.

Could the authors explain more about errors of using zero-point energy corrections and surface atom relaxation in the presence of the molecule?

System	zpe correction (kcal/mol)	$\Delta(E_{TS})$ by surface relaxation (kcal/mol)
H_2 + Cu(111)	-1.4	
H_2 + Cu(100)	-1.8	
H_2 + Pt(111)	0.0	
CH_4 + Ni(100)	-2.8	≈ -2.3
CH_4 + Ni(111)	-2.8	≈ -2.3

The reviewer is referring to the method used in Ref.17 of the previous manuscript. This paper used zero-point energy corrections, and a transition state calculation in which the surface was allowed to relax in the presence of the reacting molecule, while comparing to reference values of these barriers that were computed without zero-point energy corrections, and with a transition state calculation in which the surface was relaxed with respect to the vacuum. The size of the zpe corrections is easily given for the five systems addressed in the work to which the reviewer refers. Ref.17 (in both the present and the new version of the manuscript) took the values of the zero-point energy corrections from Nørskov and co-workers, JPCC121, 19807,2017, in which work they are listed in table 2. We have reproduced the relevant values in the above Table. These corrections amount to up to -2.8 kcal/mol for the two methane-metal surface systems investigated.

The contribution to the lowering of the transition state energy due to surface relaxation can be estimated from Nave and Jackson, JCP127, 224702, 2007. In this article they show that the motion of the surface atom below the dissociating CH_4 on Ni(111) is the main surface atom coordinate affecting the transition state energy. The lowest barrier is obtained for this surface atom puckering out of the surface by 0.23 Å (this is denoted as Q=0.23 in their paper, Q being the coordinate for the motion of the surface atom out of the surface plane). With respect to the distorted surface the barrier is already lowered by 0.2 eV for Q=0.20. Nave and Jackson report that this puckering motion increases the lattice energy by 0.1 eV. As a result, the energy of the full system is lowered by 0.1 eV, which then provides a good estimate of how the barrier height changes when the surface is allowed to relax for CH_4 + Ni(111). In the above Table we have simply assumed that the barrier height change with surface relaxation is the same for CH_4 + Ni(100) as for CH_4 + Ni(111). Values for the H_2 + metal systems are not known. As an aside we note that it makes more sense to compute reference values for barrier heights validated by comparison with supersonic

molecular beam experiments with the surface held fixed to that in contact with the vacuum also for the transition state calculation. The reason is that the surface simply does not have the time to relax in response to the fast incoming molecule, so that it is actually a good approximation to compute the sticking probability with a sudden approximation to the vibrations of the surface atoms (see e.g. B. Jackson and co-workers, Phys.Rev.Lett. 103, 253201, 2009).

An extra problem with the values reported by the authors concerned (of Ref. 17 of the previous manuscript) are that the errors they make will be roughly additive. With respect to the static vacuum surface their calculations should therefore underestimate the reference values (which, once again, are given without zero-point energy corrections, and excluding surface relaxation in the transition state) by values in the range 1.4 to 5.1 kcal/mol for the systems indicated in the above Table, but excluding H_2 + Pt(111), for which these effects will be very small. As a result, the comparison made in Ref.17 with experiments for barrier heights for dissociative chemisorption is not reliable.

To state this we have added the following short text to the paper on page 4:

For the worst case CH_4 + Ni(111) system in their small database consisting of five systems, the errors made amounted to -2.8 kcal/mol due to the zpe correction [18] and -2.3 kcal/mol due to allowing surface atom relaxation for the transition state calculation [19], yielding a total error of -5.1 kcal/mol.

E. Additional Questions:

Urgency: Moderate
Significance: Moderate
Novelty: Moderate
Scholarly Presentation: Moderate
Is the paper likely to interest a substantial number of physical chemists, not just specialists working in the authors' area of research?: No

We think that these qualifications are based on a previous incomplete understanding of our paper. We hope to have made our paper clearer, so that the reviewer will find reason to correct these qualifications in an upward manner.

Reviewer: 2

Recommendation: This paper represents a significant new contribution and should be published as is.

Comments:
The paper addresses shortcomings of DFT methods for calculations of reaction barriers by combining it with QMC approach. In particular, the idea to construct DFT functional using QMC data with subsequent much more accurate DFT study was here to be explored - the authors did solid job in this direction. The results look solid and the paper is clearly written with details in Suppl. material.

I recommend publishing in JPCL.

Additional Questions:

Urgency: High
Significance: High
Novelty: Moderate
Scholarly Presentation: High
Is the paper likely to interest a substantial number of physical chemists, not just specialists working in the authors' area of research?: Yes

We are of course glad with the recommendation provided by reviewer 2. At the same time we note that the Editor may have been negatively influenced by the brevity of the report: Reviewer 1 gives plenty of (we think incorrect) arguments for rejecting the paper, while reviewer 2 gives few arguments for why the paper should be accepted as is. We also think that reviewer 2 may have felt more positive about "Novelty" as now expressed in the answers to the "Additional questions". To us the verdict "moderate" for "novelty" seems inconsistent with the recommendation "Publish as is"; perhaps the reviewer felt that their verdict on this aspect was not important with the Recommendation provided.

jz-2023-02972d.R2

Name: Peer Review Information for "Best-of-both-worlds Predictive Approach to Dissociative Chemisorption on Metals"

Second Round of Reviewer Comments

Reviewer: 1

Comments to the Author

The authors write a good rebuttal. I appreciate the extensive work they have done to clearly explain the significant advance of this work. Overall, I am satisfied with the revised version.

Now, it becomes clear to me the acquirement of QMC data is extremely time-consuming and it is thus infeasible to do delta machine learning or transfer learning at present. The authors also make a strong argument that a single barrier height correction based on the QMC result is sufficiently accurate, at least for this system, because the barrier height variance is well captured by DFT. I have no problem on this issue any more.

Second, the authors argue that the improvement of the barrier height by this QMC-DFT adjustment will not only shift the sticking probability curve to some extent, which I think lacks of new physical insights, but also lead to radically different behaviors with respect to the vibrationally and rotationally inelastic scattering. Previous results on H_2+Cu(111) provide some evidence for supporting this argument. I concur with this argument and expect to see such calculations using this new PES in the future.
